# Imidazo[1,2-*c*]quinazolines as a novel and potent scaffold of *α*-glucosidase inhibitors: design, synthesis, biological evaluations, and in silico studies

**DOI:** 10.1038/s41598-023-42549-5

**Published:** 2023-09-21

**Authors:** Fariba Peytam, Faezeh sadat Hosseini, Malak Hekmati, Bahareh Bayati, Mahdis Sadeghi Moghadam, Zahra Emamgholipour, Loghman Firoozpour, Somayeh Mojtabavi, Mohammad Ali Faramarzi, Seyed Esmaeil Sadat-Ebrahimi, Maliheh Barazandeh Tehrani, Alireza Foroumadi

**Affiliations:** 1 Drug Design and Development Research Center, The Institute of Pharmaceutical Sciences (TIPS), Tehran University of Medical Sciences, Tehran, Iran; 2 Department of Organic Chemistry, Faculty of Pharmaceutical Chemistry, Tehran Medical Sciences, Islamic Azad University, Tehran, Iran; 3 Department of Medicinal Chemistry, Faculty of Pharmacy, Tehran University of Medical Sciences, Tehran, Iran; 4 Department of Pharmaceutical Biotechnology, Faculty of Pharmacy, Tehran University of Medical Sciences, Tehran, Iran

**Keywords:** Chemical libraries, Drug discovery and development

## Abstract

*α*-Glucosidase inhibition is an approved treatment for type 2 diabetes mellitus (T2DM). In an attempt to develop novel anti-*α*-glucosidase agents, two series of substituted imidazo[1,2-*c*]quinazolines, namely **6a–c** and **11a–o**, were synthesized using a simple, straightforward synthetic routes. These compounds were thoroughly characterized by IR, ^1^H and ^13^C NMR spectroscopy, as well as mass spectrometry and elemental analysis. Subsequently, the inhibitory activities of these compounds were evaluated against *Saccharomyces cerevisiae α*-glucosidase. In present study, acarbose was utilized as a positive control. These imidazoquinazolines exhibited excellent to great inhibitory potencies with IC_50_ values ranging from 12.44 ± 0.38 μM to 308.33 ± 0.06 μM, which were several times more potent than standard drug with IC_50_ value of 750.0 ± 1.5 μM. Representatively, compound **11j** showed remarkable anti-*α*-glucosidase potency with IC_50_ = 12.44 ± 0.38 μM, which was 60.3 times more potent than positive control acarbose. To explore the potential inhibition mechanism, further evaluations including kinetic analysis, circular dichroism, fluorescence spectroscopy, and thermodynamic profile were carried out for the most potent compound **11j**. Moreover, molecular docking studies and in silico ADME prediction for all imidazoquinazolines **6a–c** and **11a–o** were performed to reveal their important binding interactions, as well as their physicochemical and drug-likeness properties, respectively.

## Introduction

Diabetes mellitus (DM), mainly characterized as inadequate control of blood levels of glucose, has emerged as a remarkable health challenges over recent decades. Statics reveals that the rate of diabetes occurrence around the world was 536.6 million people in 2021, and this figure is predicted to reach 783.2 million people by 2045^1^. Diabetes is categorized into several subtypes with various etiologies, presentations, and treatments. Moreover, this chronic disease caused various health problems including cardiovascular diseases, hypertension, obesity, kidney diseases, and blindness. Consequently, a huge financial burden on the global health system has been imposed by this illness^2^. Considering the alarming rate of diabetes as well as complicated, severe issues associated with it, extensive efforts have already been made to manage this disease.

Diabetes mellitus is classified into several groups, including type 1, type 2, maturity-onset diabetes of the young (MODY), gestational diabetes, neonatal diabetes, and steroid-induced diabetes. Notably, the main subtypes are type 1 diabetes mellitus (T1DM) and type 2 diabetes mellitus (T2DM)^3^. These two subtypes have different pathophysiology, presentation, and management strategies. T1DM is characterized by defective insulin secretion, while T2DM involves an impaired response to insulin. However, they have a potential for hyperglycemia in common. Genetic background for both types is critical as a risk factor, but T1DM tends to occur in children, whereas T2DM is prevalent among middle-aged and older adults due to prolonged hyperglycemia resulting from their poor lifestyle and dietary choices. Statistics indicate that 1 in 11 adults suffers from diabetes mellitus, and 90% of patients have T2DM. Given this high prevalence, extensive research is currently being conducted to effectively manage this particular subtype^4,5^.

Since T2DM is mainly identified by a high level of glucose in blood (hyperglycemia), one of the pivotal strategies to control this disease is to interfere with the digestion of dietary carbohydrates. *α*-Glucosidase is an enzyme located in the brush border of the small intestine, and its role is the hydrolysis of this long chain sugar to monosaccharide units, which are subsequently released to the bloodstream. Therefore, one approved approach for the treatment of T2DM and its resultant postprandial hyperglycemia is the inhibition of *α*-glucosidase to slow down glucose absorption, thereby reducing postprandial glucose blood concentrations. Currently, there are three commercial drugs to control T2DM through the *α*-glucosidase inhibitory mechanism: acarbose, voglibose, and miglitol, among which acarbose is the most widely used and studied drug^6,7^.

**Figure 1:**
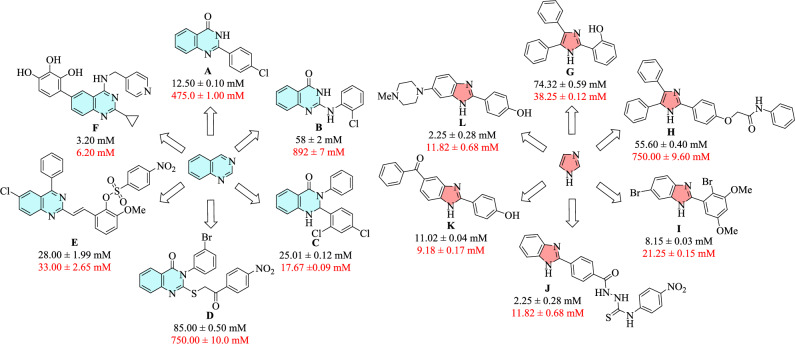
*α*-Glucosidase inhibitors bearing substituted quinazolines **A–F** and substituted imidazoles **G–L**. The IC_50_ values are written in black for inhibitors and red for acarbose.

A complex oligosaccharide, acarbose, competitively and reversibly binds to the oligosaccharide site of *α*-glucosidase in small intestine in a dose-dependent manner. This binding prevents the breakdown of disaccharide and oligosaccharide substrates into absorbable monosaccharides. Despite the efficacy of acarbose as an *α*-glucosidase inhibitor, it causes several undesirable side effects for patients, the most noticeable of which are diarrhea, abdominal discomfort, as well as bloating and flatulence^8^. Therefore, a great deal of effort over recent decade has been made to discover and develop more potent *α*-glucosidase inhibitors having improved safety and pharmacological profiles to replace acarbose. To this aim, numerous heterocyclic *α*-glucosidase inhibitors have been reported. Among them, two valuable nitrogen-containing pharmacophores, various functionalized quinazolines^9–24^ and imidazoles^25–34^ have exhibited great inhibitory potencies compared to acarbose as the standard drug. Figure 1 summarizes some structures and IC50 values of the most active compounds from these studies. Therefore, considering the proved potency of these pharmacophores, providing novel imidazole-quinazoline analog with the hope of finding further potent *α*-glucosidase inhibitors could be an interesting research topic in medicinal chemistry.

**Figure 2:**
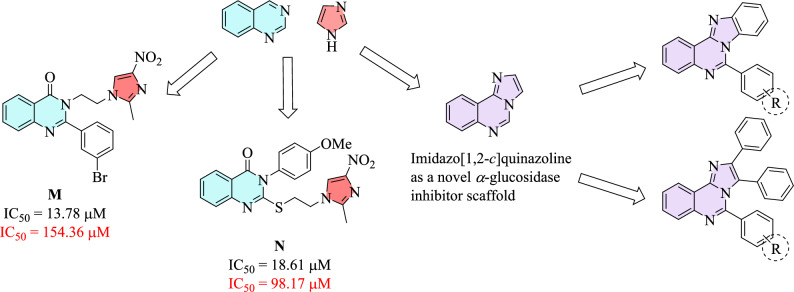
Design strategy toward two series substituted imidazo[1,2-*c*]quinazolines. The IC_50_ values are written in black for inhibitors and red for acarbose.

One well-stablished strategy for designing further novel and potent compounds is the hybridization of two scaffolds which have demonstrated promising inhibitory potencies. Various compounds bearing substituted quinazolines and imidazoles as potential *α*-glucosidase inhibitors have been already reported separately. However, there is limited investigation into the inhibitory activity of compounds containing both of these heterocycles. For example, Fig. 2 shows compounds **M** and **N** as the most potent derivatives from these studies, which were synthesized and evaluated against *α*-glucosidase, possessing noticeable inhibition in comparison with acarbose. Another strategy involves fusing two heterocycles to provide imidazoquinazoline backbone. Among several isomers of this skeleton, imidazo[1,2-*c*]quinazoline was selected for the present study. This scaffold has displayed anticancer^35^, antitubercular^36^, antifungal^37^, antimicrobial and antioxidant^38–40^ activities; however, its *α*-glucosidase inhibitory potency has yet to be explored. Therefore, this biological evaluation could be a fascinating study in medicinal chemistry (Fig. 2).

As part of our ongoing research to find potential *α*-glucosidase inhibitors^41–48^, imidazo[1,2-*c*]quinazoline was introduced as a novel inhibitor backbone in present study. To this aim, two facile, efficient synthetic protocols were employed to obtain substituted benzo[4,5]imidazo[1,2-*c*]quinazoline **6** and poly-substituted imidazo[1,2-*c*]quinazolines **11** and evaluate their in vitro potencies in comparison with acarbose as the standard drug. These compounds exhibited excellent to remarkable inhibitory activity. Subsequently, further assessments, including kinetic study, circular dichroism measurement, fluorescence quenching measurements, and thermodynamic analysis of binding to *α*‑glucosidase were carried out for the most active compound **11j**. Finally, computational investigations, including molecular docking and in silico ADME studies were performed for imidazoquinazolines **6** and **11** to investigate the mode of their interactions with the active site of *α*-glucosidase and predict the compounds’ druglike properties, respectively.

## Results and discussion

### Chemistry

**Scheme 1:**
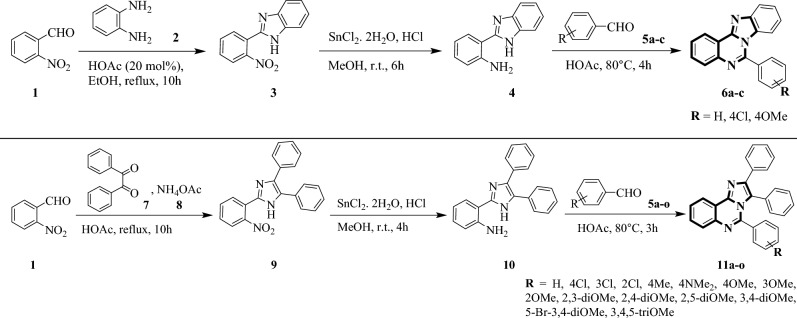
Synthesis of substituted imidazo[1,2-*c*]quinazolines **6a–c** and **11a–o**.

In present study, simple and efficient synthetic routes toward two series of substituted imidazoquinazolines **6** and **11** were performed. As illustrated in Scheme 1, the first step to obtain benzo[4,5]imidazo[1,2-*c*]quinazoline **6** was a cyclization reaction between 2-nitrobenzaldehyde **1** and benzene-1,2-diamine **2**. This reaction occurred in the presence of catalytic amount of glacial acetic acid in ethanol under the reflux conditions to afford 2-(2-Nitrophenyl)-1*H*-benzo[d]imidazole **3**. On the other hand, the protocol to obtain highly-substituted imidazo[1,2-*c*]quinazolines **11** was initiated through a cyclization reaction between 2-nitrobenzaldehyde **1**, benzil **7**, and ammonium acetate **8** under the reflux conditions in glacial acetic acid to produce 2-(2-Nitrophenyl)-4,5-diphenyl-1*H*-imidazole **9**. Subsequent steps in the synthesis were shared for both scaffolds.

The nitro functionality in compounds **3** and **9** went through the reduction reaction using stannous chloride dihydrate (SnCl_2_∙2H_2_O) and hydrochloric acid in methanol to afford the amine moiety **4** and **10**. Finally, a condensation-cyclization reaction between these adducts and substituted benzaldehydes **5** in glacial acetic acid at 80 °C occurred to obtain corresponding substituted imidazo[1,2-*c*]quinazolines **6a–c** and **11a–o** in great to excellent yields. The structures of the isolated compounds **3**, **4**, **6a–c**, **9**, **10**, and **11a–o** were deduced on the basis of their IR, ^1^H and ^13^C NMR spectroscopy, as well as mass spectrometry and elemental analysis. Partial assignments of these resonances are given in the Experimental Part.

### In vitro* α*-glucosidase inhibitory activity

The target substituted imidazo[1,2-*c*]quinazolines **6a–c** and **11a–o** were evaluated for their in vitro* Saccharomyces cerevisiae α*-glucosidase inhibitory activities to investigate the role of substituents on the imidazole moiety and the phenyl ring originated from benzaldehyde moieties. In this study, acarbose was utilized as a positive control. The obtained results were summarized in Tables 1 and 2. Our studies were initiated through the synthesis of derivatives **6a–c** as well as **11a**, **11b**, and **11g** to evaluate their potencies and reveal the role of substituents on the imidazole ring. Compounds from second series exhibited superior inhibitory activities in comparison with their analogues from the first series; therefore, we followed our studies by the synthesis and investigation of other 5-(substituted aryl)-2,3-diphenylimidazo[1,2-*c*]quinazolines **11c–f**, **h–o**.

**Table 1:** Substrate scope and in vitro α-glucosidase inhibitory activity of compounds **6a–c**.


Label	Ar	IC_50_ (µM)	Label	Ar	IC_50_ (µM)
**6a**		256.48 ± 0.14^b^	**6c**		124.28 ± 0.37^b^
**6b**		308.33 ± 0.06^b^	**Acarbose**		750.0 ± 1.5

^a^Values are the mean ± SD. All experiments were performed at least three times.

^b^p Value for all compounds was less than 0.001 in comparison with standard drug acarbose.

**Table 2:** Substrate scope and in vitro α-glucosidase inhibitory activity of compounds **11a–o**.


Label	Ar	IC_50_ (µM) ± SD	Label	Ar	IC_50_ (µM) ± SD
**11a**		209.15 ± 0.04^b^	**11i**		24.25 ± 0.13^b^
**11b**		273.28 ± 0.09^b^	**11j**		12.44 ± 0.38^b^
**11c**		246.49 ± 0.18^b^	**11k**		14.32 ± 0.05^b^
**11d**		253.08 ± 0.26^b^	**11l**		64.29 ± 0.54^b^
**11e**		124.47 ± 0.29^b^	**11m**		21.57 ± 0.32^b^
**11f**		168.36 ± 0.15^b^	**11n**		46.73 ± 0.07^b^
**11g**		82.64 ± 0.03^b^	**11o**		154.88 ± 0.36^b^
**11h**		47.92 ± 0.18^b^			
**Acarbose**		750.0 ± 1.5	**Acarbose**		750.0 ± 1.5

^a^Values are the mean ± SD. All experiments were performed at least three times.

^b^p value for all compounds was less than 0.05 in comparison with standard drug acarbose.

As illustrated in Table 2, imidazoquinazolines **11** demonstrated good to excellent *α*-glucosidase inhibitory potencies, ranging from 12.44 ± 0.38 μM to 273.28 ± 0.09 μM, in comparison with acarbose (IC_50_ = 750.0 ± 1.5 μM). The correlations between their structures and observed activities are explained comprehensively below:

To initiate, an unsubstituted phenyl ring showed moderate inhibitory potency (compound **11a**, IC_50_ = 209.15 ± 0.04 μM). Introducing a chlorine atom as an electron-withdrawing group at any position (compounds **11b**, **11c**, and **11d**) caused a detrimental effect on the *α*-glucosidase inhibitory potencies. However, replacing this atom with electron-donating groups including methyl (Me), *N*,*N*-dimethyl (N(Me)_2_), and methoxy (OMe) at C-4 position improved the inhibitory activity noticeably (compounds **11e**, **11f**, and **11g**), among which **11g** exhibited better results (IC_50_ = 82.64 ± 0.03 μM). This led us to investigate the role of this substituent at other position of phenyl ring or the presence of additional OMe group. With this in mind, other compounds were synthesized for further evaluation. Moving the OMe from C-4 to C-3 and 2 enhanced the inhibitory activity against α-glucosidase (compound **11h** with IC_50_ = 47.92 ± 0.18 μM and **11i** with IC_50_ = 24.25 ± 0.13 μM).

Considering the constructive role of OMe on the phenyl ring, particularly at C-2 position, additional OMe group was introduced. This strategy led to synthesis of two valuable compounds, namely **11j** and **11k**, which emerged as the most potent derivatives among all the synthesized imidazoquinazolines. Compound **11j** bearing two OMe groups at C-2 and 3 exhibited remarkable inhibitory potency against *α*-glucosidase IC_50_ = 12.44 ± 0.38 μM), which was 60.3 times more potent than standard inhibitor (IC_50_ = 750.0 ± 1.5 μM). Furthermore, compound **11k** with two OMe groups at C-2 and 4 ranked as the second most potent compound in this series (IC_50_ = 14.32 ± 0.05 μM). Additionally, the presence of this group at C-3 and 4 showed excellent inhibitory activity (compound **11m** with IC_50_ = 21.57 ± 0.32 μM).

Among the compounds bearing two OMe groups, **11l** showed comparatively less potency, which might be related to the deteriorative effect of C-5 position. This inference could be confirmed by comparing the results of compounds **11n** (IC_50_ = 46.73 ± 0.07 μM) and **11o** (IC_50_ = 154.88 ± 0.36 μM) with **11m** (IC_50_ = 21.57 ± 0.32 μM). It revealed that the presence of any substituent at C-5 position, whether electron-donating group like OMe or electron-withdrawing group like bromine, results in a moderate decrease in *α*-glucosidase inhibitory activity. Moreover, bromine as an electron-withdrawing group caused a detrimental effect on the inhibitory activity, which is in great agreement with earlier results in compounds **11b–d**.

A statistical analysis using the T-test was performed for both series **6a–c** and **11a–o**. All compounds indicated a significant statistical difference (p < 0.001) between the IC_50_ values of each compound in comparison with acarbose as standard drug.

Comparing the IC_50_ values of benzo[4,5]imidazo[1,2-*c*]quinazolines **6** with their corresponding analogs from 5-(substituted aryl)-2,3-diphenylimidazo[1,2-*c*]quinazolines **11** revealed the notable influence of substituents on the imidazole moiety on the *α*-glucosidase inhibitory activity, as the presence of two phenyl rings on this core improved the potency. Moreover, the aforementioned SAR analysis showed that electron-donating group, particularly OMe, improved the *α*-glucosidase inhibitory potency, while electron-withdrawing group like chlorine or bromine caused a noticeable detrimental inhibition effect. In conclusion, imidazoquinazolines bearing two OMe groups, particularly when positioned at C-2 and 3 as seen in compound **11j**, exhibited substantial inhibitory activities. Finally, compound **11j** emerged as the most potent derivative having remarkable activity against *α*-glucosidase. Consequently, it was chosen for further evaluations.

### Enzyme kinetic study

**Figure 3:**
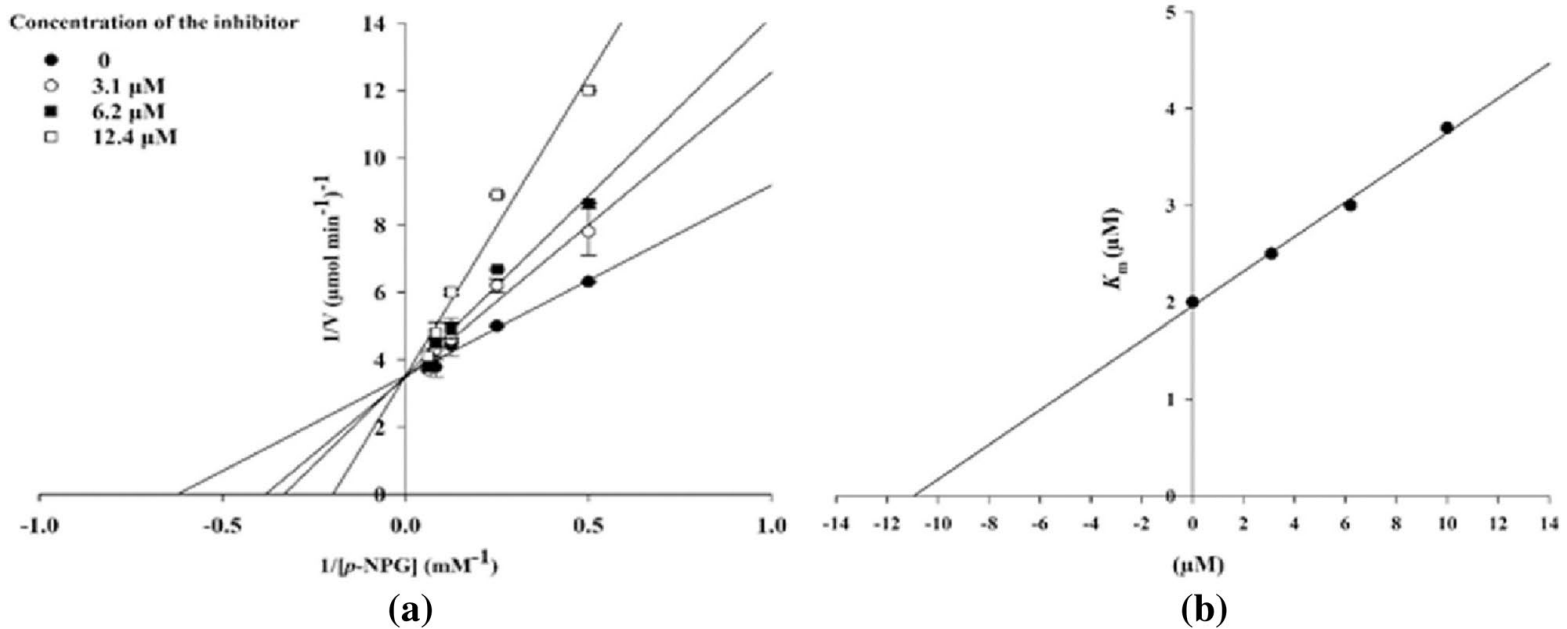
Kinetics of *α*-glucosidase inhibition by sample **11j**: (**A**) the Lineweaver–Burk plot in the absence and presence of different concentrations of sample **11j**; (**B**) the secondary plot between K_m_ and various concentrations of sample **11j**.

The enzyme kinetic study was performed to reveal the inhibition mode of imidazoquinazoline **11j**. There are two enzyme kinetic constants: Michaelis constant (K_m_) and maximum velocity of the reaction (V_max_) which are calculated using initial velocity measurements at different inhibitors concentrations (for example, 0, 3.1, 6.2, and 12.4 μM in present study). As illustrated in Fig. 3A, the Lineweaver–Burk plot exhibited the K_m_ value increased with increasing concentration of compound **11j**, while V_max_ did not change. The results indicated that this imidazoquinazoline bonded to the active site on the enzyme and competes with the substrate for binding to this region, indicating a competitive type of inhibition. Moreover, the plot of the K_m_ versus different concentrations of inhibitor gave an estimate of the inhibition constant as K_i_ value of 11.0 µM (Fig. 3B).

### Circular dichroism spectroscopy

The difference between the absorption of right and left circularly polarized light is measured in circular dichroism spectroscopy (CD) in order to reveal the chiral environment around amino acid residues. The CD spectrum in the far ultraviolet region ranged from 190 to 240 nm is mainly used to provide valuable information about the arrangement of protein bonds and secondary structure of the proteins in dilute solutions. There are several principal conformations like *α*-helix, extended *β* structure (or *β*-sheet), *β*-turn, and random coil (which are unordered structures). They are characterized as follow: *α*-helix structures by negative CD bands at 222 and 208 nm and a positive CD band at approximately 190 nm; *β*-sheet structures by a negative CD band in the region of 210–220 nm; *β*-turn structures by a negative CD band between 180 and 190 nm; and the spectra of random coil by a characteristic negative CD band in region of 200 nm^49,50^.

**Figure 4:**
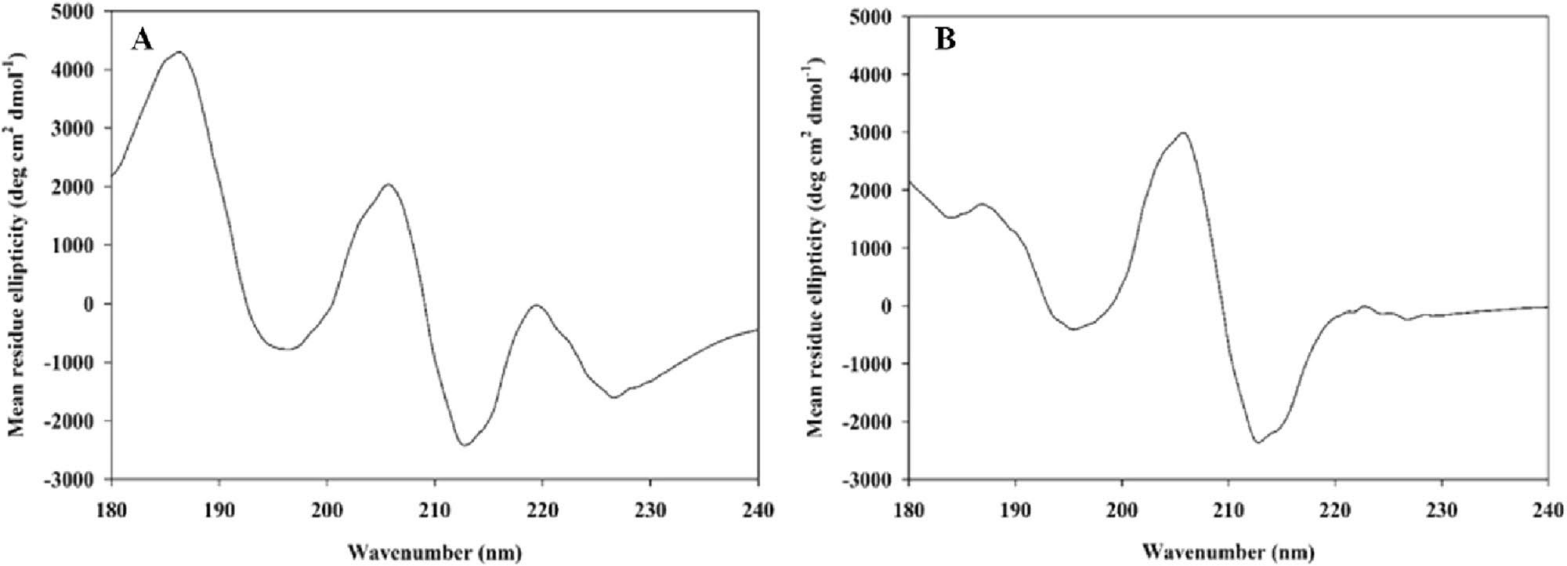
Circular dichroism (CD) spectra of the *α*-glucosidase: (**A**) in the absence of inhibitor (control); (**B**) in the presence of imidazoquinazoline **11j**.

To study the impact of imidazo[1,2-*c*]quinazoline **11j** on the secondary structure of *α*-glucosidase (Fig. 4b), the CD spectra (180–250 nm) was measured and analyzed using the CDNN software to be compared with the native enzyme (Fig. 4a). The percent of observed conformations are summarized in Table 3. As it can be seen, our inhibitor increased noticeably the figures for *α*-helix and *β*-turn; while random coils removed; therefore, this imidazo[1,2-*c*]quinazoline **11j** can determine the conformation of the enzyme and fix chiral side chains in orientations. Moreover, this compound can change the secondary structure of *α*-glucosidase, resulting to inhibit its performance.

**Table 3:** The secondary structure content of *α*-glucosidase.

Inhibitor	*α*-Helix (%)	*ꞵ*-Turn (%)	Random coil (%)
Control^a^	28.8	28.8	42.4
Imidazoquinazoline **11j**^b^	50.3	49.7	0

^a^Control is native enzyme in the absence of an inhibitor.

^b^The concentration of imidazoquinazoline **11j** was 12.4 μM.

### Fluorescence spectroscopy measurements

Fluorescence spectroscopy assay is a frequently used method to investigate the potential interactions between inhibitors and enzymes under physiological conditions, because binding of inhibitors changes the fluorescence characteristics and tertiary structure of the protein. Moreover, it can predict the tertiary structure of the enzyme and provide more accurate information about the binding constant, number of binding sites, and thermodynamic parameters of the studied interactions.

**Figure 5:**
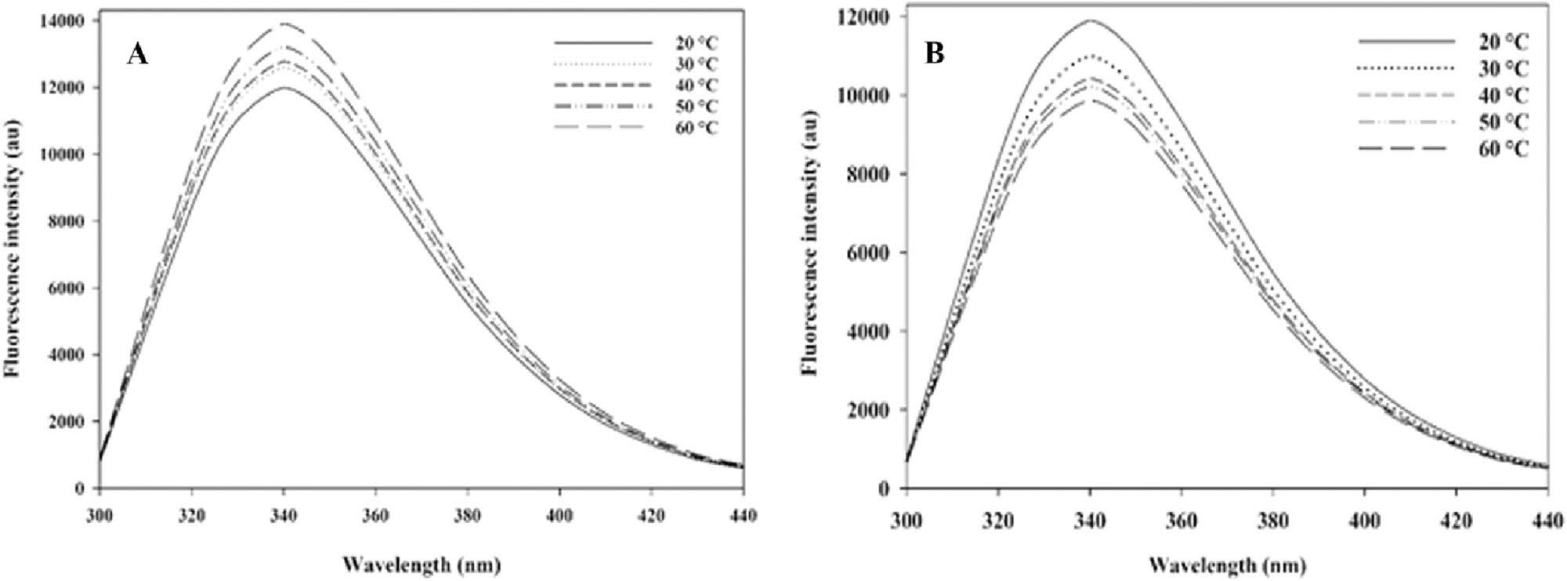
Fluorescence spectra of *α*-glucosidase: (**A**) in the absence of compound **11j** at 20–60 ℃, (**B**) in the presence of compound **11j** at inhibitory concentration (12.4 µM) at 20–60 ℃.

In present study, fluorescence spectroscopy measurement was performed between imidazo[1,2-*c*]quinazoline **11j** and the active site of enzyme using a Synergy HTX multi-mode reader (Biotek Instruments, Winooski, VT, USA) equipped with a quartz cuvette of 10 mm. The excitation wavelength was 280 nm, and the emission spectra were reported at five different temperatures in the range from 300 to 450 nm with 10 accumulations for each collection point. The emission spectrum was adjusted for the background fluorescence from the buffer solution and for the inner filter effect promoted by the inhibitors (Fig. 5).

The results obtained from this evaluation revealed that the fluorescence intensity of *α*-glucosidase increased to 340 nm and subsequently decreased (the λ_max_ was 340 nm). On the other hand, there are three amino acids—tryptophan, tyrosine, and phenylalanine—that play a role in the enzyme's intrinsic fluorescence property, referred to as fluorophores. Among them, the maximum intensity of tryptophan at 280 nm is about 340 nm; therefore, imidazo[1,2-*c*]quinazoline **11j** must be located proximity to the tryptophan residues within the *α*-glucosidase binding site when this inhibitor bound to the enzyme and changed the tertiary structure of the enzyme.

There are two types of fluorescence quenching: dynamic and static. Dynamic quenching arises from the collisional encounter between the fluorophore (tryptophan residues) and the quencher (inhibitor). Conversely, static quenching results from the formation a ground‐state complex between fluorophore and quencher. Results revealed that the combination of fluorophores (tryptophan residues) and quencher (imidazo[1,2-*c*]quinazoline **11j**) exhibited a static quenching mechanism. Therefore, the biding parameters can be determined as follow:

The reaction is determined as P + D → D_n_P in which P is the protein, D is the drug molecule (inhibitor), and D_n_P is the new complex molecule. The binding constant for this complex for this complex, denoted as K_A_, is calculated using Eq. (1). In the static quenching mechanism, the number of binding sites, which is named “n”, remains constant. Since the number of the binding site of protein and drug is n and 1, respectively, the equivalent concentration of the complex D_n_P is n[D_n_P]. Moreover, the equivalent concentration of the protein is n[P], and the equivalent concentration of the drug is [D]:

1 $${\text{K}}_{{\text{A}}} = \frac{{{\text{n}}\left[ {{\text{D}}_{{\text{n}}} {\text{P}}} \right]}}{{\left[ {\text{D}} \right]{\text{n}}\left[ {\text{P}} \right]}}$$

The total concentration of protein is [P_t_], and the total concentration of the drug is [D_t_]; therefore, [P_t_] = [P_f_] + [D_n_P] and [D_f_] = [D_t_]−n[D_n_P]. Since protein (P) is the only fluorescence in present study, thus:

2 $$\frac{{{\text{F}}_{0} }}{{\text{F}}} = \frac{{\left[ {{\text{P}}_{{\text{t}}} } \right]}}{{\left[ {{\text{P}}_{{\text{f}}} } \right]}}$$

F and F_0_ are the fluorescence intensity of protein in the presence and absence of inhibitor, respectively. Therefore, the relationship between the fluorescence intensity and the total concentration of the drug could be deduced:

3 $$\frac{{{\text{F}}_{0} }}{{\text{F}}} = \frac{{{\text{K}}_{{\text{A}}} \left[ {{\text{D}}_{{\text{t}}} } \right]{\text{F}}_{0} }}{{\left( {{\text{F}}_{0} { }{-}{\text{ F}}} \right){ }{-}{\text{ n K}}_{{\text{A}}} { }\left[ {{\text{P}}_{{\text{t}}} } \right]}}$$

**Figure 6:**
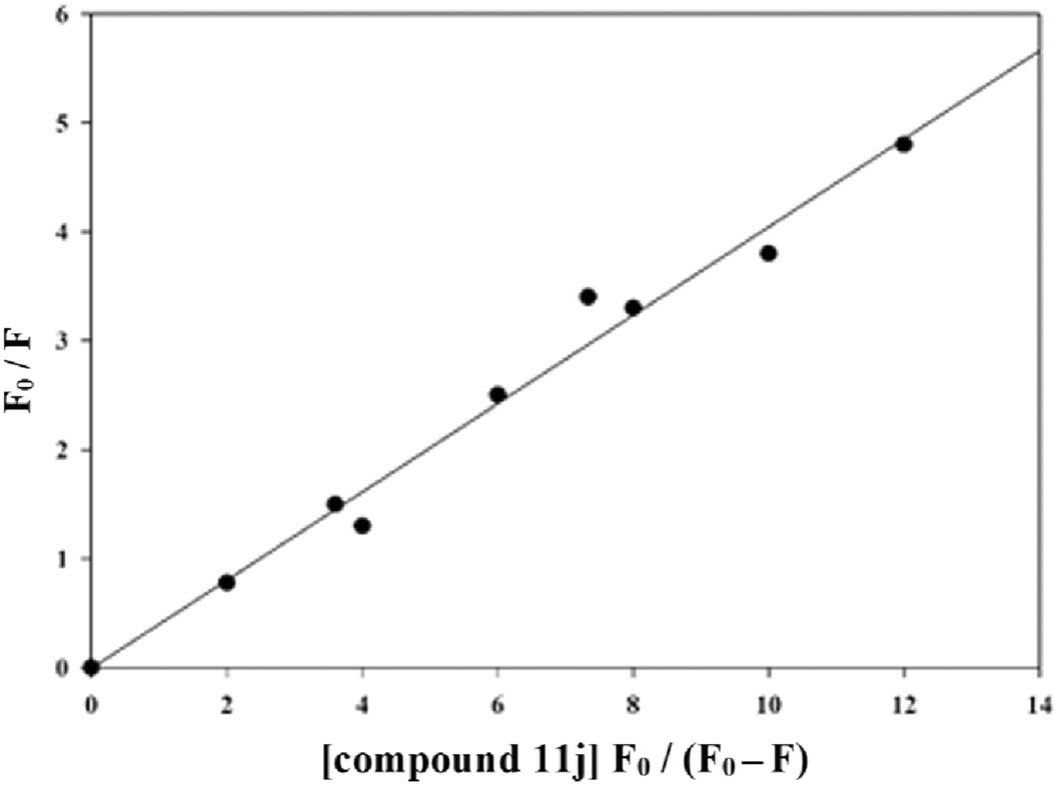
The plots F_0_/F Vs. function of [D_t_] F_0_/(F_0_−F) at 20 ℃ for imidazoquinazoline **11j**.

As the total concentration of protein was kept at a constant value (at 46 nM), while the total concentration of the drug was changed. Using the Eq. (3), a plot of F_0_/F Vs. [D_t_] F_0_/(F_0_−F) was obtained, as depicted in Fig. 6. K_A_, n, and r at 20 ℃ can also be calculated, as listed in Table 4:

**Table 4:** Binding constants and binding sites for imidazoquinazoline **11j**.

K_A_ (L mol^–1^ s^–1^)^a^	K_A_ (L mol^–1^ s^–1^)^b^	n^b^	r^b^
36.6 × 10^4^	40.5 × 10^4^	0.3	0.997

^a^Temperature is 60 ℃.

^b^Temperature is 20 ℃.

### Thermodynamic analysis of binding of imidazoquinazoline 11j to *α*‑glucosidase

This fluorescent intensity data was plotted as a function of temperature and binding constants; therefore, thermodynamic profile including ΔG (free energy change), ΔH (enthalpy change), and ΔS (entropy change) could be computed to determine the type of non-covalent forces between imidazo[1,2-*c*]quinazoline **11j** and binding site of *α*‑glucosidase. These forces between the protein and inhibitor can be categorized into four groups: hydrogen bond, van der Waals forces, electrostatic attraction, and hydrophobic interactions. To identify the type of interactions in present study, thermodynamic parameters must be calculated using following equations:

4 $${\text{ln}}\frac{{{\text{K}}_{{{\text{A}}2}} }}{{{\text{K}}_{{{\text{A}}1}} }} = \frac{{{\Delta H}}}{{\text{R}}}{ }\left( {\frac{1}{{{\text{T}}_{2} }} - { }\frac{1}{{{\text{T}}_{1} }}} \right)$$

5 $$\Delta {\text{G }} = \, {-}{\text{ RT ln K}}_{{\text{A}}} = \, \Delta {\text{H }}{-}{\text{ T}}\Delta {\text{S}}$$

Already, there was some information including binding constants (K_A2_ = 36.6 × 10^4^ and K_A1_ = 40.5 × 10^4^) as well as initial and final temperatures (T_1_ = 20 ℃ (293 K) and T_2_ = 60 ℃ (333 K)). Therefore, using Eq. (4), ΔH is obtained − 2.17 (kJ mol^–1^) and subsequently using Eq. (5), ΔG and ΔS values are calculated − 20.6 (kJ mol^–1^) and 62.9 (J mol^–1 ^K^–1^), respectively.

Considering the sign of these thermodynamic parameters, the type of non-covalent force could be determined as follow: (1) ΔH > 0, ΔS > 0, hydrophobic interactions; (2) ΔH < 0, ΔS > 0, van der Waals forces; (3) ΔH < 0, ΔS < 0, hydrogen bond and van der Waals interactions; and (4) ΔH < 0, ΔS > 0, electrostatic interactions. Therefore, the obtained results indicated that the acting force between imidazoquinazoline **11j** and *α*‑glucosidase was mainly determined as electrostatic forces^51,52^.

### Molecular docking studies

Molecular docking study was conducted using AutoDock4 and Auto Dock Tools (version 1.5.6) to explore the interaction patterns of substituted benzo[4,5]imidazo[1,2-*c*]quinazoline **6a–c** and imidazo[1,2-*c*]quinazolines **11a–o** within the active site of human acid-*α*-glucosidase (PDB ID: 5NN8). This PBD ID was also used in previous studies^53^. Observed interactions are listed in Table 5. The binding energy of imidazo[1,2-*c*]quinazolines **11a–o** were found to be in range of − 7.62 to − 8.59 kcal mol^−1^, which is noticeably better than that of acarbose (− 3.79 kcal mol^−1^). The docking protocol validation involved a redocking study using the crystallized ligand with PDB ID of 5NN8. This process resulted to a low RMSD value of 1.57, confirming the reliability of our docking studies.

**Table 5:** Interactions of compounds **11a–o** with crystal structure of human acid-*α*-glucosidase using BIOVIA Discovery Studio visualizer v21.1.0.20298 and PLIP online service.

Compound	Binding energy (Kcal mol^−1^)	Interactions^a^	Moiety	Residue
**11a**	− 8.07	Hydrophobic interactions		
**11b**	− 7.79	Hydrogen bond	Imidazoquinazoline	ARG600
		π-Cation Interaction	Phenyl	ARG600
**11c**	− 8.02	Hydrophobic interactions		
**11d**	− 8.01	Hydrophobic interactions		
**11e**	− 7.71	Hydrogen bond	Imidazoquinazoline	ARG600
		π-Cation Interaction	Phenyl	ARG600
**11f**	− 8.50	π-Stacking	Phenyl	PHE649
**11g**	− 8.05	Hydrogen bond	methoxy	ALA284
**11h**	− 7.62	Hydrogen bond	Methoxy	ALA284
**11i**	− 7.71	Hydrophobic interactions		
**11j**	− 8.50	Hydrogen bond	Methoxy	ALA284
**11k**	− 8.35	Hydrogen bond	Methoxy	ALA284
		π-Stacking	Phenyl	PHE649
**11l**	− 8.28	Hydrogen bond	Methoxy	ALA284
**11m**	− 7.65	Hydrogen bond	Imidazoquinazoline	ARG600
		π-Cation Interaction	Phenyl	ARG600
**11n**	− 8.07	Hydrogen bond	Methoxy	ALA284
**11o**	− 8.59	Hydrogen bond	Methoxy	ALA284

^a^Hydrophobic interactions for compounds with hydrogen bonds or other types of interactions were not mentioned.

**Figure 7:**
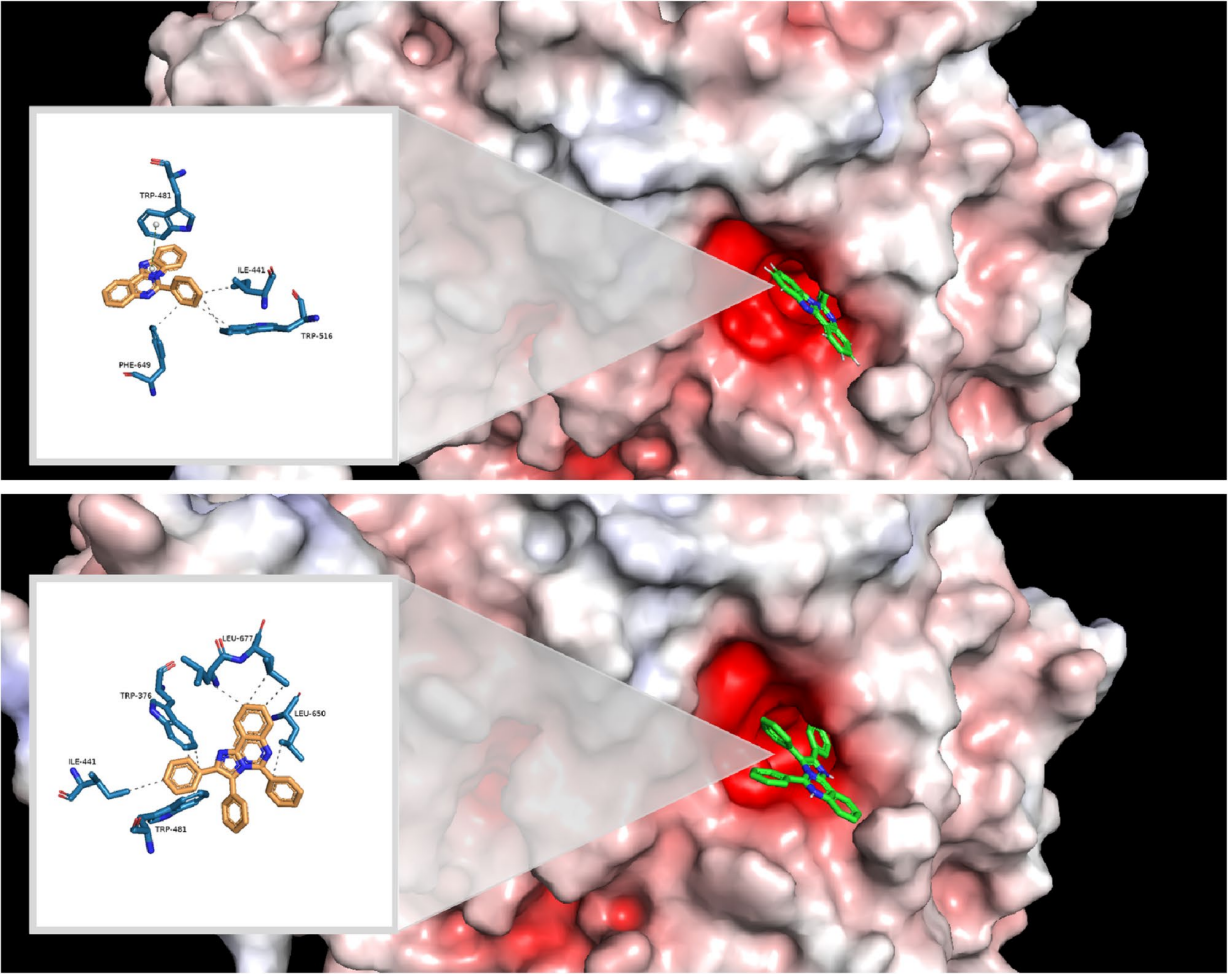
Interactions and structures of (**a**) compound **6a** and (**b**) compound **11a** in the binding pocket of human acid-*α*-glucosidase visualized using PyMOL 2.5.2 and PLIP online service.

Additionally, the similar computational process was conducted for benzo[4,5]imidazo[1,2-*c*]quinazolines **6a–c**, showing the binding energy scores between − 7.30 to − 7.58 kcal mol^−1^. These values were moderately lower than the binding energies in second series. These figures were found to be − 8.07, − 7.79, and − 8.05 kcal mol^−1^ for compounds **11a**, **11b**, and **11g**, respectively; however, they were − 7.30, − 7.58, and − 7.32 kcal mol^−1^ for compounds **6a**, **6b**, and **6c**, respectively. It might be related to its fewer interactions. This issue is illustrated by comparing compounds **6a** with **11a** as shown in Fig. 7. It must be noted no hydrogen bond formation was observed, and hydrophobic interactions are shown by dashed lines.

As previously described in SAR analysis, derivatives bearing electron-donating groups, particularly OMe (compounds **11e–o**), exhibited the excellent to great inhibitory activities. Herein, molecular docking studies (as presented in Table 6) revealed that these compounds **11e-o**, except **11i**, created a hydrogen bond with OMe moiety or imidazoquinazoline backbone, which might be responsible for their significant inhibitory potencies.

**Table 6:** The prediction of pharmacokinetic’s parameters of imidazoquinazolines 11a**–**o and 6a**–**c by SWISSADME.

Compound	MW	Consensus Log P	GI absorption	Bioavailability Score	Rotatable bonds	H-bond acceptors	H-bond donors
**11a**	397.47	3.78	Low	0.55	3	2	–
**11b**	431.92	4.06	Low	0.55	3	2	–
**11c**	431.92	3.92	Low	0.55	3	2	–
**11d**	431.92	3.93	Low	0.55	3	2	–
**11e**	411.50	3.96	Low	0.55	3	2	–
**11f**	440.54	4.08	Low	0.55	4	3	–
**11g**	427.50	3.91	Low	0.55	4	3	–
**11h**	427.50	4.2	Low	0.55	5	4	–
**11i**	427.50	3.9	Low	0.55	4	2	–
**11j**	457.52	4.15	Low	0.55	5	4	–
**11k**	457.52	4.11	Low	0.55	5	4	–
**11l**	457.52	4.09	Low	0.55	6	5	–
**11m**	457.52	4.22	Low	0.55	5	4	–
**11n**	487.55	4.02	Low	0.55	4	3	–
**11o**	536.42	4.05	Low	0.17	5	4	–
**6a**	295.34	4.11	High	0.55	2	0	–
**6b**	329.78	4.69	High	0.55	2	0	–
**6c**	325.36	4.15	High	0.55	3	0	–

**Figure 8:**
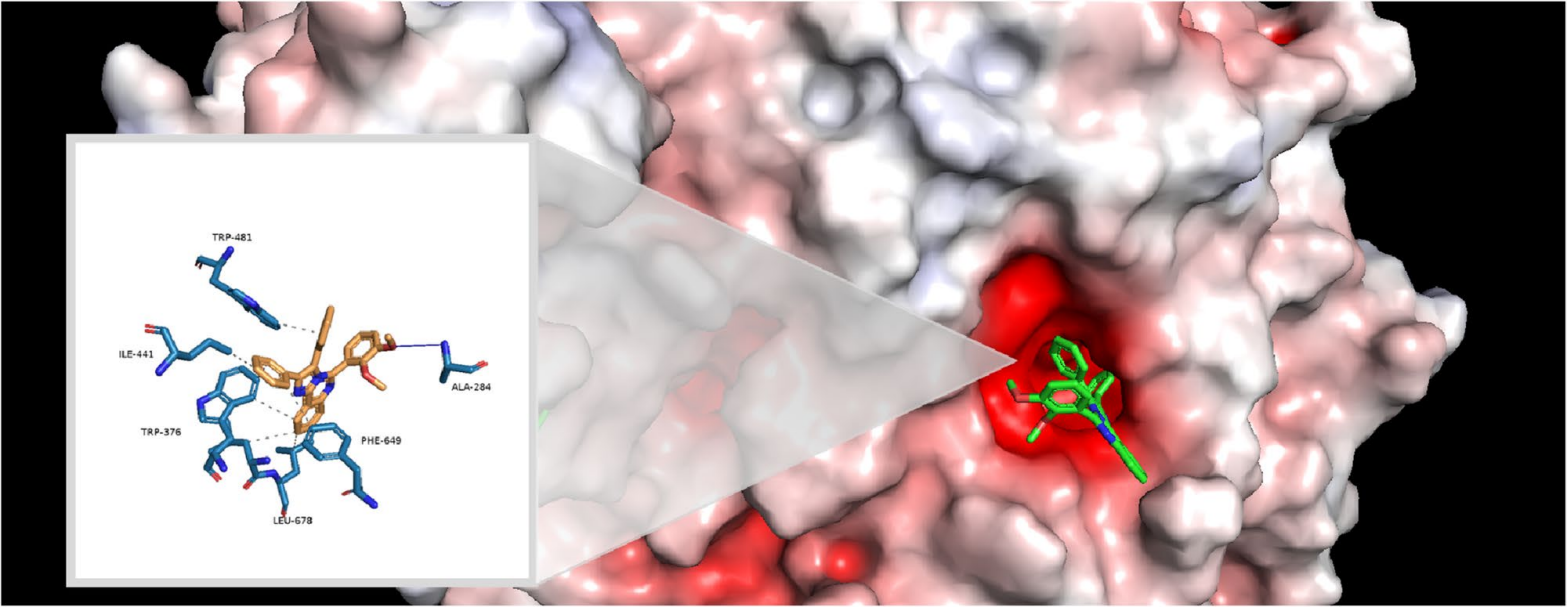
The interactions and structure of compound **11j** in the binding pocket of human acid-*α*-glucosidase (it must be noted that the hydrogen bond and hydrophobic interactions are displayed in blue color and dashed lines, respectively).

**Figure 9:**
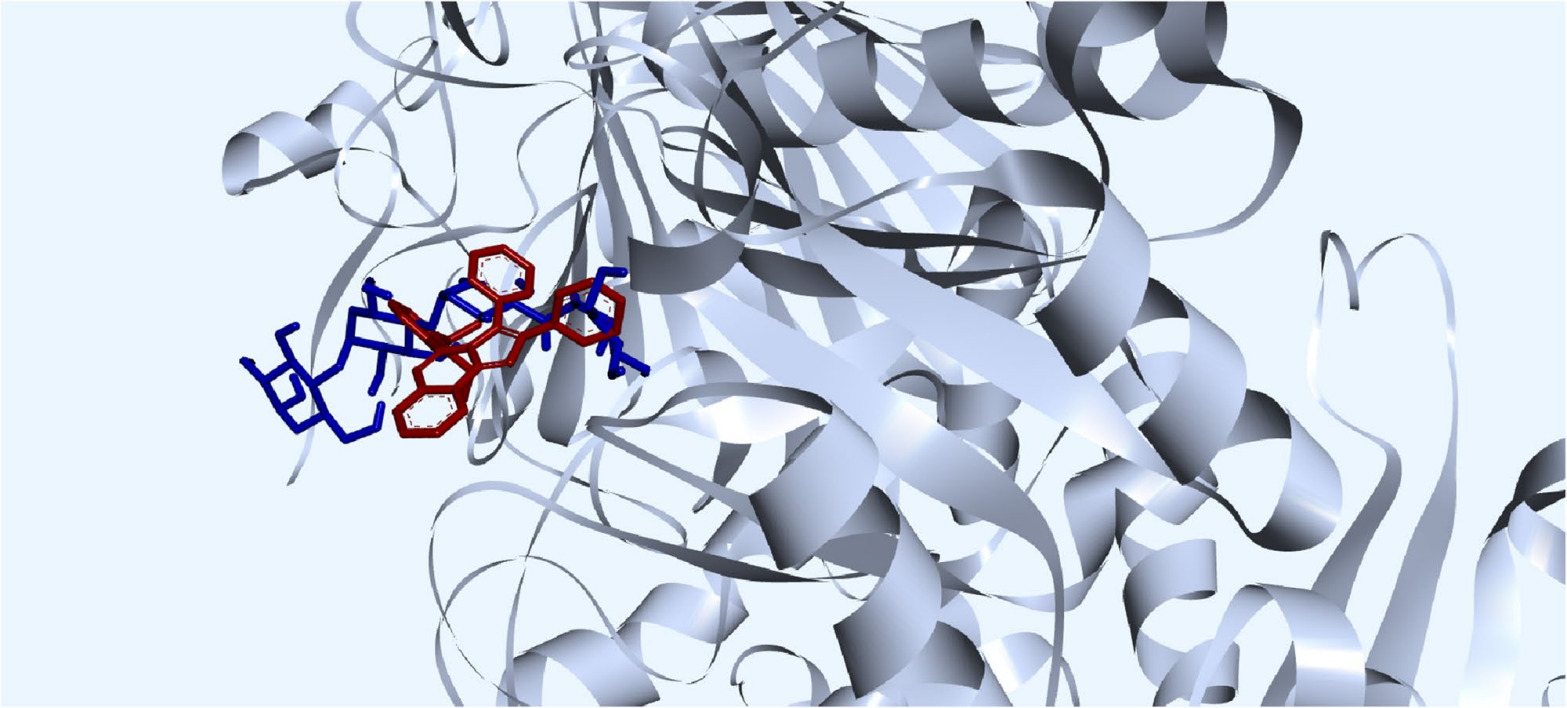
Superimposition of acarbose and compound **11j** in the binding pocket of human acid-α-glucosidase. Acarbose is colored in blue, and compound **11j** is colored in red.

Imidazo[1,2-*c*]quinazoline **11j**, the most potent compound in present study with the best IC_50_ value (12.44 ± 0.38 μM), exhibited a noticeable binding energy of − 8.50 kcal mol^−1^. This affinity could be attributed to the formation of a hydrogen bond between the OMe moiety at C-3 position and ALA-284 residue within the receptor. This similar hydrogen bond was observed in the complex of acarbose and receptor in the crystal structure. Additionally, compound **11j** formed several hydrophobic interactions with different residues including TRP-481, TRP-376, LEU-678, PHE-649, and ILE-441 within the active site of *α*-glucosidase (Fig. 8). Similar hydrophobic interactions between acarbose and some residues in the binding site of the receptor (like TRP-481 and TRP-376) were observed in the redocking results. The presence of these interactions with tryptophan residues is consistent with the results of fluorescence spectroscopy measurements. Overall, similar interactions between both compound **11j** and acarbose with active site of* α*-glucosidase, as well as their superimposition as depicted in Fig. 9, can confirm the accuracy of the docking procedure and the validity of results.

### In silico ADME

The ADME parameters of benzo[4,5]imidazo[1,2-*c*]quinazolines **6a–c** and imidazo[1,2-*c*]quinazolines **11a–o** were calculated using SwissADME online server^54^. The results are summarized in Table 6. All compounds possessed favorable drug-likeliness, and they were mostly consistent with Lipinski’s Rule of 5. FDA-approved *α*-glucosidase inhibitors, particularly acarbose, possess low oral bioavailability and act as a competitive, reversible inhibitor of membrane-bound intestinal enzyme. Considering the presence of *α*-glucosidase in the lumen and its mechanism, it was assumed that low Human intestinal absorption (HIA) of imidazoquinazolines **11a–o** would be a promising factor to observe minimum systemic adverse effects, while being sufficiently effective in the lumen environment^55,56^. However, second series, including compounds **6a–c** were predicted to have high HIA, indicating their potentially lower pharmacological activities.

**Figure 10:**
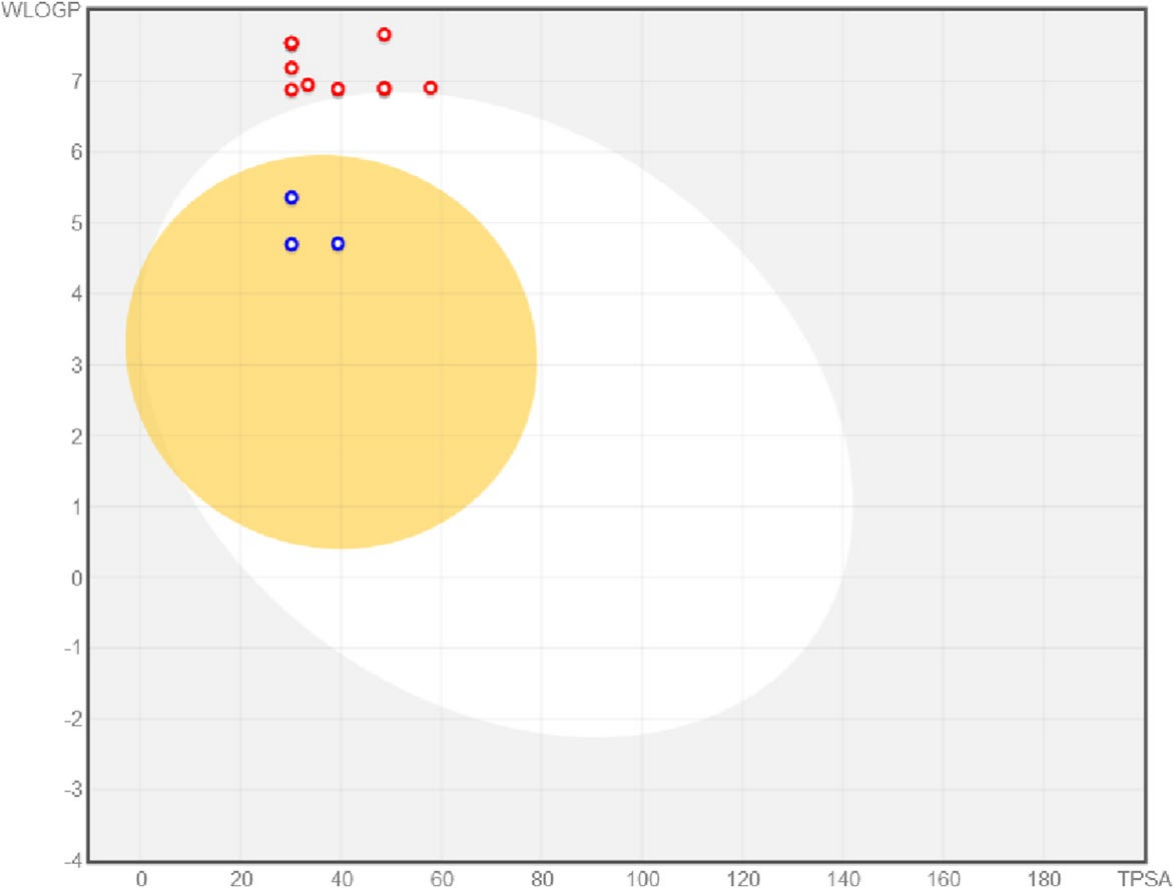
Compounds **6a–c** and **11a–o** were examined by the boiled-egg method available on SWISS ADME.

Since acarbose acts in the gastrointestinal tract, its low systemic absorption (below 2% of the administered dose) is crucial for optimal therapeutic efficacy^57^. In Fig. 10, passive gastro-intestinal absorption (HIA) and blood–brain barrier (BBB) permeation were predicted by the BOILED-Egg model^58^. In this figure, benzo[4,5]imidazo[1,2-*c*]quinazolines **6a–c** were shown by blue dots. Imidazo[1,2-*c*]quinazolines **11a–o** were shown by red dots, and some of them were overlapped by each other. Compounds which are located in the yellow region are predicted to have BBB permeability. Benzo[4,5]imidazo[1,2-*c*]quinazolines **6a–c** were predicted to be BBB permeable, which is a negative feature for using these compounds as *α*‑glucosidase inhibitors. However, none of imidazo[1,2-*c*]quinazolines **11a–o** were anticipated to be BBB-permeable, which is ideal for *α*‑glucosidase inhibitor safety profile.

Moreover, compounds which are located in the white area are predicted to have good absorption. As previously discussed, considering the enzyme’s site of action (lumen environment), high bioavailability may cause side effects without any improvement on the efficacy. Overall, benzo[4,5]imidazo[1,2-*c*]quinazolines **6a–c** possessed higher bioavailability, which is not favorable for *α*‑glucosidase inhibitory activity in the gastrointestinal tract, while imidazo[1,2-*c*]quinazolines **11a–o** were able to act locally in the lumen without getting into the bloodstream. Therefore, compounds **11a–o** with low HIA and no BBB-permeation could be potential candidates for further studies.

## Conclusion

In attempt to find novel and potent *α*‑glucosidase inhibitors, efficient synthetic approaches were performed to synthesize substituted imidazo[1,2-*c*]quinazolines **6** and **11**. Their inhibitory potencies were evaluated, showing excellent to great potencies (ranged from 12.44 ± 0.38 μM to 308.33 ± 0.06 μM) in comparison with acarbose (IC_50_ = 750.0 ± 1.5 μM). Notably, compound **11j** exhibited the most potent inhibitory activity, therefore, it was selected for further evaluations including kinetic analysis, circular dichroism, fluorescence spectroscopy, and thermodynamic profile. It was observed that imidazoquinazoline **11j** compete with the substrate for binding to the binding site of *α*‑glucosidase. Moreover, circular dichroism and fluorescence spectroscopy measurements confirmed that this binding led to change the secondary and tertiary structure of enzyme and inhibit its performance. Calculation of thermodynamic parameters including ΔG (free energy change), ΔH (enthalpy change), and ΔS (entropy change) values revealed the construction of spontaneous, electrostatic forces between imidazoquinazoline **11j** and *α*‑glucosidase. The importance of the presence of electron-donating groups such as OMe was verified during the docking procedure, since an important hydrogen bond was formed in the mentioned compounds. Also, the superiority of compounds **11** over **6** was confirmed by the low HIA figures of imidazoquinazoline **11** in ADME studies. Overall, these results showed that our target imidazoquinazolines could be considered as a promising hit for further development of *α*‑glucosidase inhibitors as a well-stablished diabetes treatment approach.

## Experimental

All chemicals were purchased from Merck (Germany) and were used without further purification. Melting points were measured on an Electrothermal 9100 apparatus. Elemental analyses for C, H and N were performed using a Heraeus CHN-O-Rapid analyzer. Mass spectra were recorded on an Agilent Technologies (HP) 5973 mass spectrometer operating at an ionization potential of 20 eV. IR spectra were recorded on a Shimadzu IR-460 spectrometer. ^1^H and ^13^C NMR spectra were measured (DMSO-*d*_6_ solution) with Bruker DRX-300 (at 300.1 and 75.5 MHz) and Bruker DRX-500 AVANCE (at 500.1 and 125.8 MHz) instruments.

### General synthetic procedures

#### General procedure for the preparation of 2-(2-Nitrophenyl)-1*H*-benzo[*d*]imidazole 3:

A mixture of 2-nitrobenzaldehyde **1** (8.0 mmol, 1.208 g), benzene-1,2-diamine **2** (6.7 mmol, 0.723 g), and glacial acetic acid (20 mol%, 1.34 mmol, 0.076 ml) in EtOH (15 ml) was heated reflux conditions within 10 h. As the completion of compound **3** was confirmed by TLC analysis, the reaction mixture was quenched by water, and the resulting precipitation was filtered and washed completely. Afterwards, it was recrystallized by EtOAc and *n*-Hexane (within the proportion of 3:1) to afford the desirable compound 3 as a pure orange solid in 78% yield.

2-(2-Nitrophenyl)-1*H*-benzo[*d*]imidazole **3**: Orange solid, mp 264–267 °C, yield: 78%. IR (KBr) (ν_max_/cm^-1^): 3329 (NH), 1622, 1553, 1488, 1403, 1396, 1348, 1285, 1233, 1177, 1093, 1061, 949, 913, 880, 858, 746, 693. ^1^H NMR (500.1 MHz, DMSO-*d*_6_): *δ* 10.78 (br s., 1H, NH), 8.03 (d, *J* = 8.2 Hz, 1H, CH), 7.99 (d, *J* = 7.7 Hz, 1H, CH), 7.86 (t, *J* = 7.6 Hz, 1H, CH), 7.74 (t, *J* = 7.3 Hz, 1H, CH), 7.71–7.60 (m, 2H, 2CH), 7.30–7.20 (m, 2H, 2CH). ^13^C NMR (125.8 MHz, DMSO-*d*_6_): *δ* 150.55, 147.43, 143.38, 135.49, 133.24, 131.92, 130.98, 124.78, 123.12, 120.13, 112.78. ESI–MS m/z: 240.48 [M + 1]^+^. Anal. Calcd. for C_13_H_9_N_3_O_2_: C, 65.27; H, 3.79; N, 17.56.; found: C, 65.08; H, 4.05; N, 17.78%.

#### General procedure for the preparation of 2-(2-Nitrophenyl)-4,5-diphenyl-1*H*-imidazole 9

A mixture of 2-nitrobenzaldehyde **1** (36 mmol, 5.436 g), benzil **7** (30 mmol, 6.302 g), and ammonium acetate **8** (300 mmol, 23.125 g) in glacial acetic acid (75 ml) were heated under the reflux conditions for 10 h. After completion of the reaction, which was monitored by TLC, the mixture was cooled down to room temperature, and it was gradually poured into crushed ice. The yellow solid residue got to precipitate, filtered, and washed with water. Finally, it was recrystallized from EtOH (40 ml) to obtain the pure adduct **9** as yellow powder in 84% yield.

2-(2-Nitrophenyl)-4,5-diphenyl-1*H*-imidazole **9**: Yellow solid, mp 236–240 °C, yield: 84%. IR (KBr) (ν_max_/cm^–1^): 3348 (NH), 1592, 1549, 1487, 1436, 1396, 1343, 1296, 1247, 1168, 1108, 1089, 974, 903, 849, 826, 737, 640. ^1^H NMR (500.1 MHz, DMSO-*d*_6_): *δ* 10.23 (br s., 1H, NH), 8.29 (d, *J* = 8.0 Hz, 1H, CH), 7.98 (d, *J* = 7.8 Hz, 1H, CH), 7.70 (t, *J* = 7.7 Hz, 1H, CH), 7.62 (t, *J* = 7.5 Hz, 1H, CH), 7.38–7.20 (m, 10H, 10CH). ^13^C NMR (125.8 MHz, DMSO-*d*_6_): *δ* 148.98, 146.38, 141.09, 134.75, 133.30, 132.21, 130.68, 128.45, 127.74, 125.91, 124.68. ESI–MS m/z: 342.84 [M + 1]^+^. Anal. Calcd. for C_21_H_15_N_3_O_2_: C, 73.89; H, 4.43; N, 12.31.; found: C, 74.12; H, 4.68; N, 12.56%.

#### General procedure for the reduction of nitro functionality to amine moiety (compounds 4 and 10):

The procedure was common for both series: to a mixture of 2-(2-Nitrophenyl)-1*H*-benzo[*d*]imidazole **4** (5.20 mmol, 1.243 g) in hydrochloric acid (12.48 ml) and MeOH (5 ml) in ice bath at 0 °C, stannous chloride dihydrate SnCl_2_.2H_2_O (17.16 mmol, 3.878 g) was added gradually within 1 h. Afterwards, the mixture was stirred at room temperature for almost 6 h till the yellow color of nitro moiety got disappeared. As compound **3** was completely used, and it was confirmed by TLC analysis, the reaction mixture was basified by a solution of NaOH (2N) to pH 8. Then, water (20 ml) was added to the mixture and extracted three times with EtOAc (3 × 45 ml). The combined organic extracts were washed with brine, dried over Na_2_SO_4_, and then concentrated. The precipitate was filtered and washed with Et_2_O to afford pure product **4** as white powder in 58% yield.

2-(1*H*-benzo[*d*]imidazol-2-yl)aniline **4**: White solid, mp 208–211 °C, yield: 58%. IR (KBr) (ν_max_/cm^–1^): 3184, 3058, and 3024 (3NH), 1611, 1519, 1476, 1429, 1279, 1178, 1056, 908, 882, 795, 723, 689, 633. ^1^H NMR (500.1 MHz, DMSO-*d*_6_): *δ* 7.81 (d, *J* = 7.8 Hz, 1H, CH), 7.76–7.69 (m, 2H, 2CH), 7.62–7.54 (m, 2H, 2CH), 7.49 (t, *J* = 7.8 Hz, 1H, CH), 7.10 (t, *J* = 7.5 Hz, 1H, CH), 6.68 (dd, *J* = 7.3 and 0.9 Hz, 1H, CH), 3.84–3.42 (br. s, 3H, NH and NH_2_). ^13^C NMR (125.8 MHz, DMSO-*d*_6_): *δ* 153.78, 147.86, 138.56, 131.08, 127.98, 122.76, 117.66, 115.38, 114.93, 110.38. ESI–MS m/z: 209.96 [M]^+^. Anal. Calcd. for C_13_H_11_N_3_: C, 74.62; H, 5.30; N, 20.08.; found: C, 74.49; H, 5.08; N, 20.23%.

In a similar procedure, 2-(2-Nitrophenyl)-4,5-diphenyl-1*H*-imidazole **4** (25.2 mmol, 8.593 g) in hydrochloric acid (60.48 ml) and MeOH (30 ml) in ice bath at 0 °C, stannous chloride dihydrate SnCl_2_.2H_2_O (83.16 mmol, 18.794 g) was added slowly within 1 h. The reaction took 4 h to get finish. After workup, pure white powder compound **10** was obtained in 63% yield.

2-(4,5-diphenyl-1*H*-imidazol-2-yl)aniline **10**: White solid, mp 218–220 °C, yield: 63%. IR (KBr) (ν_max_/cm^–1^): 3408, 3369, and 3238 (3NH), 1622, 1467, 1417, 1297, 1233, 1177, 1093, 1061, 949, 913, 869, 844, 758, 673. ^1^H NMR (500.1 MHz, DMSO-*d*_6_): *δ* 10.24 (s, 1H, NH), 7.96 (d, *J* = 7.8 Hz, 1H, CH), 7.53–7.20 (m, 11H, 11CH), 6.94 (t, *J* = 7.6 Hz, 1H, CH), 6.68 (d, *J* = 7.4 Hz, 1H, CH), 5.23 (s, 2H, NH_2_). ^13^C NMR (125.8 MHz, DMSO-*d*_6_): *δ* 147.36, 146.98, 135.87, 132.45, 129.65, 128.78, 127.65, 126.24, 116.84, 115.53, 111.64. ESI–MS m/z: 311.78 [M]^+^. Anal. Calcd. for C_21_H_17_N_3_: C, 81.00; H, 5.50; N, 13.49.; found: C, 80.82; H, 5.39; N, 13.75%.

#### General procedure for the preparation of poly-substituted imidazo[1,2-*c*]quinazolines 6a-c and 11a-o

A mixture of synthesized 2-(1*H*-benzo[d]imidazol-2-yl)aniline **4** or 2-(4,5-diphenyl-1*H*-imidazol-2-yl)aniline **10** (1 mmol, g) with corresponding substituted benzaldehyde **5** (1.5 mmol) in glacial acetic acid (5 ml) was magnetically stirred at 80 °C for almost 3 to 4 h. After completion of reaction confirmed by TLC analysis, the mixture was cooled to the room temperature and poured into water. The precipitate was filtered, washed with water, and recrystallized from EtOAc to obtain the desired imidazoquinazolines **6a–c** and **11a–o** as pure powder in good to excellent yields.

6-phenylbenzo[4,5]imidazo[1,2-*c*]quinazoline **6a**: Yellow solid, mp 156–158 °C, yield: 86%. IR (KBr) (ν_max_/cm^–1^): 1596, 1498, 1433, 1378, 1294, 1263, 1182, 1123, 1082, 993, 825, 755, 685, 634. ^1^H NMR (500.1 MHz, DMSO-*d*_6_): *δ* 8.23 (d, *J* = 7.8 Hz, 1H, CH), 8.16 (d, *J* = 7.7 Hz, 1H, CH), 7.74 (d, *J* = 8.0 Hz, 1H, CH), 7.40–7.16 (m, 8H, 8CH), 6.95 (d, *J* = 7.9 Hz, 1H, CH), 6.86 (t, *J* = 7.4 Hz, 1H, CH). ^13^C NMR (125.8 MHz, DMSO-*d*_6_): *δ* 145.47, 144.13, 139.07, 136.66, 134.20, 130.87, 129.48, 129.05, 128.98, 126.20, 125.79, 124.57, 124.11, 118.43, 115.87, 115.22, 111.83, 107.42. ESI–MS m/z: 295.87 [M]^+^. Anal. Calcd. for C_20_H_13_N_3_: C, 81.34; H, 4.44; N, 14.23.; found: C, 81.66; H, 4.28; N, 13.99%.

6-(4-chlorophenyl)benzo[4,5]imidazo[1,2-*c*]quinazoline **6b**: Yellow solid, mp 173–175 °C, yield: 92%. IR (KBr) (ν_max_/cm^–1^): 1526, 1487, 1423, 1385, 1348, 1343, 1288, 1225, 1173, 1074, 1042, 1013, 911, 845, 827, 780, 769, 697. ^1^H NMR (500.1 MHz, DMSO-*d*_6_): *δ* 7.94 (d, *J* = 7.6 Hz, 1H, CH), 7.63 (d, *J* = 7.9 Hz, 1H, CH), 7.52 (d, *J* = 7.4 Hz, 1H, CH), 7.30–6.92 (m, 7H, 7CH), 6.84 (d, *J* = 7.8 Hz, 1H, CH), 6.81 (t, *J* = 7.2 Hz, 1H, CH). ^13^C NMR (125.8 MHz, DMSO-*d*_6_): *δ* 146.91, 143.55, 143.24, 138.42, 137.44, 132.77, 129.59, 129.26, 127.41, 126.28, 124.66, 122.24, 122.08, 118.52, 118.15, 114.81, 111.70, 110.60. ESI–MS m/z: 329.43 [M]^+^. Anal. Calcd. for C_20_H_12_ClN_3_: C, 72.84; H, 3.67; N, 12.74.; found: C, 73.06; H, 3.42; N, 12.96%.

6-(4-methoxyphenyl)benzo[4,5]imidazo[1,2-*c*]quinazoline **6c**: Yellow solid, mp 198–201 °C, yield: 79%. IR (KBr) (ν_max_/cm^–1^): 1592, 1498, 1403, 1387, 1292, 1226, 1198, 1132, 1068, 1015, 937, 847, 785, 769, 684, 623. ^1^H NMR (500.1 MHz, DMSO-*d*_6_): *δ* 7.95 (d, *J* = 7.6 Hz, 1H, CH), 7.63 (d, *J* = 7.9 Hz, 1H, CH), 7.48 (d, *J* = 7.8 Hz, 1H, CH), 7.32–6.90 (m, 6H, 6CH), 6.88 (d, *J* = 8.2 Hz, 2H, 2CH), 6.81 (t, *J* = 7.8 Hz, 1H, CH), 3.68 (s, 3H, OCH_3_). ^13^C NMR (125.8 MHz, DMSO-*d*_6_): *δ* 159.72, 146.97, 143.62, 143.39, 132.80, 132.34, 131.69, 130.42, 127.52, 124.67, 122.18, 122.03, 118.52, 118.14, 114.80, 114.09, 111.72, 110.66, 55.15. ESI–MS m/z: 310.74 [M + 1]^+^. Anal. Calcd. for C_21_H_15_N_3_O: C, 84.53; H, 4.89; N, 13.58.; found: C, 84.76; H, 5.14; N, 13.84%.

2,3,5-triphenylimidazo[1,2-*c*]quinazoline **11a**: Milky solid, mp 133–137 °C, yield: 78%. IR (KBr) (ν_max_/cm^–1^): 1598, 1496, 1456, 1412, 1368, 1289, 1263, 1148, 1129, 1067, 1026, 989, 965, 897, 822, 752, 689, 636. ^1^H NMR (500.1 MHz, DMSO-*d*_6_): *δ* 7.88 (d, *J* = 7.5 Hz, 1H, CH), 7.50 (d, *J* = 7.2 Hz, 2H, 2CH), 7.45–7.00 (m, 14H, 14CH), 6.84 (d, *J* = 7.1 Hz, 1H, CH), 6.82 (t, *J* = 6.9 Hz, 1H, CH). ^13^C NMR (125.8 MHz, DMSO-*d*_6_): *δ* 141.37, 140.93, 137.50, 133.94, 130.32, 130.13, 129.38, 129.05, 128.91, 128.54, 128.37, 128.18, 126.42, 125.10, 124.86, 123.19, 122.82, 113.24. ESI–MS m/z: 397.26 [M]^+^. Anal. Calcd. for C_28_H_19_N_3_: C, 84.61; H, 4.82; N, 10.57.; found: C, 84.93; H, 4.62; N, 10.78%.

5-(4-chlorophenyl)-2,3-diphenylimidazo[1,2-*c*]quinazoline **11b**: Milky solid, mp 178–181 °C, yield: 94%. IR (KBr) (ν_max_/cm^–1^): 1589, 1488, 1463, 1399, 1348, 1294, 1231, 1168, 1129, 1087, 996, 929, 886, 795, 756, 718, 675, 638. ^1^H NMR (500.1 MHz, DMSO-*d*_6_): *δ* 7.90 (d, *J* = 7.2 Hz, 1H, CH), 7.50 (d, *J* = 7.8 Hz, 2H, 2CH), 7.42–7.00 (m, 13H, 13CH), 6.96 (d, *J* = 7.9 Hz, 1H, CH), 6.85 (t, *J* = 7.6 Hz, 1H, CH). ^13^C NMR (125.8 MHz, DMSO-*d*_6_): *δ* 141.13, 140.80, 139.66, 137.17, 133.42, 133.02, 130.35, 129.10, 129.02, 128.57, 128.20, 127.10, 126.84, 126.60, 126.38, 123.33, 122.97, 112.73. ESI–MS m/z: 431.86 [M]^+^. Anal. Calcd. for C_28_H_18_ClN_3_: C, 77.86; H, 4.20; N, 9.73.; found: C, 78.04; H, 4.06; N, 9.53%.

5-(3-chlorophenyl)-2,3-diphenylimidazo[1,2-*c*]quinazoline **11c**: Milky solid, mp 149–151 °C, yield: 83%. IR (KBr) (ν_max_/cm^–1^): 1586, 1509, 1447, 1367, 1320, 1286, 1184, 1129, 1068, 1023, 967, 834, 754, 685, 622. ^1^H NMR (300.1 MHz, DMSO-*d*_6_): *δ* 7.97 (d, *J* = 7.7 Hz, 1H, CH), 7.76 (d, *J* = 7.9 Hz, 2H, 2CH), 7.60–7.17 (m, 11H, 11CH), 7.11 (d, *J* = 8.0 Hz, 2H, 2CH), 6.91 (d, *J* = 7.2 Hz, 1H, CH), 6.79 (t, *J* = 7.6 Hz, 1H, CH). ^13^C NMR (75.1 MHz, DMSO-*d*_6_): *δ* 141.81, 141.10, 135.39, 131.27, 131.06, 130.80, 129.62, 129.55, 129.50, 129.10, 128.99, 128.76, 128.69, 127.15, 127.02, 126.95, 125.42, 123.67, 119.06, 115.63. ESI–MS m/z: 432.29 [M + 1]^+^. Anal. Calcd. for C_28_H_18_ClN_3_: C, 77.86; H, 4.20; N, 9.73.; found: C, 77.94; H, 4.13; N, 9.89%.

5-(2-chlorophenyl)-2,3-diphenylimidazo[1,2-*c*]quinazoline **11d**: Milky solid, mp 164–167 °C, yield: 72%. IR (KBr) (ν_max_/cm^–1^): 1599, 1498, 1438, 1399, 1358, 1294, 1256, 1162, 1129, 1089, 1012, 991, 932, 899, 836, 763, 729, 687, 624. ^1^H NMR (500.1 MHz, DMSO-*d*_6_): *δ* 8.04 (d, *J* = 8.0 Hz, 1H, CH), 7.87 (dd, *J* = 7.2, 0.9 Hz, 1H, CH), 7.74 (t, *J* = 7.7 Hz, 2H, 2CH), 7.68–7.17 (m, 13H, 13CH), 6.96 (d, *J* = 7.4 Hz, 1H, CH), 6.83 (t, *J* = 7.8 Hz, 1H, CH). ^13^C NMR (125.8 MHz, DMSO-*d*_6_): *δ* 147.90, 144.90, 138.14, 137.79, 136.29, 135.96, 133.42, 131.58, 130.82, 130.11, 129.81, 129.10, 128.30, 127.74, 127.25, 126.35, 124.84, 123.57, 119.94, 113.92. ESI–MS m/z: 432.56 [M]^+^. Anal. Calcd. for C_28_H_18_ClN_3_: C, 77.86; H, 4.20; N, 9.73.; found: C, 78.06; H, 4.54; N, 9.48%.

5-(4-methylphenyl)-2,3-diphenylimidazo[1,2-*c*]quinazoline **11e**: Milky solid, mp 155–157 °C, yield: 87%. IR (KBr) (ν_max_/cm^–1^): 1595, 1489, 1402, 1387, 1285, 1216, 1194, 1143, 1098, 1034, 995, 865, 746, 658, 624. ^1^H NMR (500.1 MHz, DMSO-*d*_6_): *δ* 7.96 (d, *J* = 7.8 Hz, 1H, CH), 7.84 (d, *J* = 7.8 Hz, 1H, CH), 7.78 (s, 1H, CH), 7.70–7.00 (m, 13H, 13CH), 6.96 (d, *J* = 7.8 Hz, 1H, CH), 6.82 (t, *J* = 7.4 Hz, 1H, CH), 2.15 (s, 3H, CH_3_). ^13^C NMR (125.8 MHz, DMSO-*d*_6_): *δ* 141.42, 141.04, 138.06, 137.64, 137.52, 134.05, 130.27, 130.02, 129.45, 129.05, 128.15, 126.55, 126.35, 125.04, 124.74, 123.13, 122.76, 113.47, 20.57. ESI–MS m/z: 412.37 [M + 1]^+^. Anal. Calcd. for C_29_H_21_N_3_: C, 84.64; H, 5.14; N, 10.21.; found: C, 84.78; H, 4.98; N, 10.38%.

4-(2,3-diphenylimidazo[1,2-*c*]quinazolin-5-yl)-*N*,*N*-dimethylaniline **11f**: Milky solid, mp 208–210 °C, yield: 76%. IR (KBr) (ν_max_/cm^–1^): 1594, 1487, 1438, 1422, 1368, 1276, 1239, 1188, 1052, 1028, 985, 913, 852, 758, 695. ^1^H NMR (500.1 MHz, DMSO-*d*_6_): *δ* 8.12 (d, *J* = 8.1 Hz, 2H, 2CH), 7.91 (d, *J* = 7.8 Hz, 1H, CH), 7.53 (d, *J* = 7.5 Hz, 2H, 2CH), 7.48–7.00 (m, 9H, 9CH), 6.92 (d, *J* = 7.7 Hz, 1H, CH), 6.80 (t, *J* = 7.4 Hz, 1H, CH), 6.53 (d, *J* = 8.1 Hz, 2H, 2CH), 2.81 (s, 6H, 2 NCH_3_). ^13^C NMR (125.8 MHz, DMSO-*d*_6_): *δ* 154.65, 141.92, 141.32, 134.15, 130.07, 129.18, 129.09, 128.97, 128.60, 128.26, 127.20, 126.81, 126.74, 126.35, 124.96, 123.07, 112.28, 111.52, 31.44. ESI–MS m/z: 441.69 [M + 1]^+^. Anal. Calcd. for C_30_H_24_N_4_: C, 81.79; H, 5.49; N, 12.72.; found: C, 82.02; H, 5.72; N, 12.48%.

5-(4-methoxyphenyl)-2,3-diphenylimidazo[1,2-*c*]quinazoline **11g**: Milky solid, mp 189–192 °C, yield: 79%. IR (KBr) (ν_max_/cm^–1^): 1599, 1496, 1428, 1392, 1348, 1294, 1223, 1188, 1138, 1094, 1046, 991, 952, 889, 836, 756, 733, 678, 633. ^1^H NMR (500.1 MHz, DMSO-*d*_6_): *δ* 7.97 (d, *J* = 7.4 Hz, 1H, CH), 7.70–7.06 (m, 13H, 13CH), 6.94 (d, *J* = 7.8 Hz, 1H, CH), 6.85 (t, *J* = 7.5 Hz, 1H, CH), 6.76 (d, *J* = 7.8 Hz, 2H, 2CH), 3.65 (s, 3H, OCH_3_). ^13^C NMR (125.8 MHz, DMSO-*d*_6_): *δ* 158.91, 148.21, 144.89, 138.18, 136.99, 136.79, 136.41, 130.27, 129.85, 129.66, 129.22, 128.64, 127.94, 127.35, 124.86, 120.02, 113.90, 111.51, 55.11. ESI–MS m/z: 427.83 [M]^+^. Anal. Calcd. for C_29_H_21_N_3_O: C, 81.48; H, 4.95; N, 9.83.; found: C, 81.34; H, 5.12; N, 10.04%.

5-(3-methoxyphenyl)-2,3-diphenylimidazo[1,2-*c*]quinazoline **11h**: Milky solid, mp 174–176 °C, yield: 87%. IR (KBr) (ν_max_/cm^–1^): 1595, 1498, 1422, 1377, 1287, 1253, 1163, 1109, 1096, 1034, 987, 857, 763, 749, 684, 633. ^1^H NMR (300.1 MHz, DMSO-*d*_6_): *δ* 7.86 (d, *J* = 7.4 Hz, 1H, CH), 7.50 (d, *J* = 7.5 Hz, 2H, 2CH), 7.45–7.00 (m, 10H, 10CH), 6.90–6.70 (m, 3H, 3CH), 6.41 (d, *J* = 7.6 Hz, 1H, CH), 6.35 (s, 1H, CH), 3.58 (s, 3H, OCH_3_). ^13^C NMR (75.1 MHz, DMSO-*d*_6_): *δ* 159.12, 142.52, 141.44, 141.07, 137.56, 134.01, 130.30, 130.10, 129.79, 129.41, 129.09, 128.90, 128.18, 126.61, 126.41, 123.15, 122.79, 113.36, 111.52, 110.86, 54.94. ESI–MS m/z: 428.44 [M + 1]^+^. Anal. Calcd. for C_29_H_21_N_3_O: C, 81.48; H, 4.95; N, 9.83.; found: C, 81.62; H, 5.23; N, 9.68%.

5-(2-methoxyphenyl)-2,3-diphenylimidazo[1,2-*c*]quinazoline **11i**: Milky solid, mp 148–152 °C, yield: 69%. IR (KBr) (ν_max_/cm^–1^): 1593, 1472, 1436, 1399, 1358, 1276, 1212, 1196, 1153, 1090, 1041, 1023, 975, 856, 795, 769, 713, 642, 625. ^1^H NMR (300.1 MHz, DMSO-*d*_6_): *δ* 7.88 (d, *J* = 7.1 Hz, 1H, CH), 7.50 (d, *J* = 6.9 Hz, 2H, 2CH), 7.45–6.98 (m, 10H, 10CH), 6.95–6.85 (m, 2H, 2CH), 6.78 (t, *J* = 7.3 Hz, 1H, CH), 6.71 (d, *J* = 7.6 Hz, 1H, CH), 6.37 (d, *J* = 7.3 Hz, 1H, CH), 3.64 (s, 3H, OCH_3_). ^13^C NMR (75.1 MHz, DMSO-*d*_6_): *δ* 154.87, 141.90, 141.00, 137.85, 134.34, 130.30, 130.11, 129.88, 129.58, 128.85, 128.70, 128.30, 128.12, 126.42, 126.18, 122.93, 122.58, 113.32, 111.46, 111.22, 55.54. ESI–MS m/z: 428.44 [M + 1]^+^. Anal. Calcd. for C_29_H_21_N_3_O: C, 81.48; H, 4.95; N, 9.83.; found: C, 81.34; H, 4.78; N, 10.04%.

5-(2,3-dimethoxyphenyl)-2,3-diphenylimidazo[1,2-*c*]quinazoline **11j**: Milky solid, mp 221–224 °C, yield: 63%. IR (KBr) (ν_max_/cm^–1^): 1597, 1502, 1437, 1411, 1379, 1292, 1229, 1143, 1077, 1027, 991, 943, 899, 847, 788, 753, 706, 686, 652. ^1^H NMR (500.1 MHz, DMSO-*d*_6_): *δ* 8.06 (d, *J* = 8.5 Hz, 1H, CH), 7.51 (d, *J* = 7.4 Hz, 2H, 2CH), 7.48–7.10 (m, 10H, 10CH), 6.95 (d, *J* = 8.2 Hz, 1H, CH), 6.87 (d, *J* = 7.9 Hz, 1H, CH), 6.82 (t, *J* = 8.3 Hz, 1H, CH), 6.05 (d, *J* = 7.8 Hz, 1H, CH), 3.90 and 3.75 (2s, 6H, 2OCH_3_). ^13^C NMR (125.8 MHz, DMSO-*d*_6_): *δ* 152.70, 151.86, 144.72, 141.90, 141.51, 133.58, 132.15, 131.02, 129.73, 129.52, 128.78, 127.10, 126.75, 124.50, 123.90, 118.76, 117.24, 115.69, 113.90, 108.38, 60.20, 56.19. ESI–MS m/z: 457.96 [M]^+^. Anal. Calcd. for C_30_H_23_N_3_O_2_: C, 78.75; H, 5.07; N, 9.18.; found: C, 78.99; H, 5.23; N, 9.36%.

5-(2,4-dimethoxyphenyl)-2,3-diphenylimidazo[1,2-*c*]quinazoline **11k**: Milky solid, mp 197–200 °C, yield: 71%. IR (KBr) (ν_max_/cm^–1^): 1589, 1456, 1398, 1361, 1273, 1237, 1198, 1104, 1036, 1021, 998, 943, 836, 759, 724, 639. ^1^H NMR (500.1 MHz, DMSO-*d*_6_): *δ* 8.06 (d, *J* = 8.5 Hz, 1H, CH), 7.53–7.00 (m, 11H, 11CH), 6.95–6.65 (m, 4H, 4CH), 6.05 (s, 1H, CH), 3.54 and 3.51 (2s, 6H, 2OCH_3_). ^13^C NMR (125.8 MHz, DMSO-*d*_6_): *δ* 153.14, 151.88, 149.77, 142.12, 141.61, 137.31, 130.84, 129.77, 129.39, 128.92, 128.60, 128.20, 127.44, 126.88, 124.11, 118.81, 115.57, 114.12, 113.12, 112.89, 56.48, 55.65. ESI–MS m/z: 457.53 [M]^+^. Anal. Calcd. for C_30_H_23_N_3_O_2_: C, 78.75; H, 5.07; N, 9.18.; found: C, 78.58; H, 4.96; N, 8.92%.

5-(2,5-dimethoxyphenyl)-2,3-diphenylimidazo[1,2-*c*]quinazoline **11l**: Milky solid, mp 183–186 °C, yield: 67%. IR (KBr) (ν_max_/cm^–1^): 1597, 1512, 1489, 1423, 1395, 1335, 1295, 1258, 1183, 1124, 1075, 1031, 992, 968, 899, 831, 796, 740, 685, 673, 642. ^1^H NMR (500.1 MHz, DMSO-*d*_6_): *δ* 7.88 (d, *J* = 7.6 Hz, 1H, CH), 7.60–7.00 (m, 11H, 11CH), 6.05 (s, 1H, CH), 6.94 (d, *J* = 7.8 Hz, 1H, CH), 6.80 (t, *J* = 8.0 Hz, 1H, CH), 6.48 (s, 1H, CH), 6.33 (d, *J* = 7.5 Hz, 1H, CH), 6.29 (d, *J* = 7.5 Hz, 1H, CH), 3.54 and 3.51 (2s, 6H, 2OCH_3_). ^13^C NMR (125.8 MHz, DMSO-*d*_6_): *δ* 161.11, 156.52, 142.30, 141.66, 138.00, 132.84, 130.63, 130.30, 129.33, 129.20, 128.62, 126.81, 126.41, 123.28, 121.32, 118.75, 117.01, 115.63, 105.05, 99.04, 56.11, 55.62. ESI–MS m/z: 458.64 [M + 1]^+^. Anal. Calcd. for C_30_H_23_N_3_O_2_: C, 78.75; H, 5.07; N, 9.18.; found: C, 78.93; H, 5.26; N, 9.33%.

5-(3,4-dimethoxyphenyl)-2,3-diphenylimidazo[1,2-*c*]quinazoline **11m**: Milky solid, mp 234–237 °C, yield: 84%. IR (KBr) (ν_max_/cm^–1^): 1595, 1522, 1424, 1386, 1296, 1259, 1178, 1154, 1078, 1014, 997, 947, 923, 805, 768, 743, 695, 629. ^1^H NMR (300.1 MHz, DMSO-*d*_6_): *δ* 8.04 (d, *J* = 7.2 Hz, 1H, CH), 7.65–7.15 (m, 11H, 11CH), 6.91 (d, *J* = 6.9 Hz, 1H, CH), 6.85 (t, *J* = 7.5 Hz, 1H, CH), 6.77 (d, *J* = 8.1 Hz, 1H, CH), 6.64 (s, 2H, 2CH), 6.33 (d, *J* = 8.1 Hz, 1H, CH), 3.64 and 3.57 (2s, 6H, 2OCH_3_). ^13^C NMR (75.8 MHz, DMSO-*d*_6_): *δ* 156.31, 155.22, 149.29, 148.94, 142.30, 141.30, 133.04, 131.80, 130.88, 129.66, 129.31, 129.01, 128.36, 128.04, 124.26, 119.09, 117.71, 115.73, 111.84, 109.70, 55.84, 55.82. ESI–MS m/z: 458.32 [M + 1]^+^. Anal. Calcd. for C_30_H_23_N_3_O_2_: C, 78.75; H, 5.07; N, 9.18.; found: C, 78.92; H, 4.87; N, 9.36%.

2,3-diphenyl-5-(3,4,5-trimethoxyphenyl)imidazo[1,2-*c*]quinazoline **11n**: Milky solid, mp 256–260 °C, yield: 78%. IR (KBr) (ν_max_/cm^–1^): 1595, 1539, 1426, 1399, 1358, 1289, 1243, 1167, 1143, 1089, 1045, 987, 935, 829, 778, 685, 645. ^1^H NMR (300.1 MHz, DMSO-*d*_6_): *δ* 8.07 (d, *J* = 7.0 Hz, 1H, CH), 7.60–7.12 (m, 11H, 11CH), 6.97 (d, *J* = 8.1 Hz, 1H, CH), 6.87 (t, *J* = 7.4 Hz, 1H, CH), 6.23 (s, 2H, 2CH), 3.56 and 3.53 (2s, 9H, 3OCH_3_). ^13^C NMR (75.1 MHz, DMSO-*d*_6_): *δ* 156.79, 155.21, 146.36, 141.69, 139.71, 138.88, 137.07, 134.82, 130.89, 130.63, 130.39, 129.78, 129.36, 128.89, 128.42, 124.83, 120.04, 115.26, 58.36, 56.72. ESI–MS m/z: 488.26 [M + 1]^+^. Anal. Calcd. for C_31_H_25_N_3_O_3_: C, 76.37; H, 5.17; N, 8.62.; found: C, 76.18; H, 4.98; N, 8.48%.

5-(3-bromo-4,5-dimethoxyphenyl)-2,3-diphenylimidazo[1,2-*c*]quinazoline **11o**: Milky solid, mp 287–289 °C, yield: 90%. IR (KBr) (ν_max_/cm^–1^): 1623, 1584, 1532, 1465, 1354, 1298, 1253, 1198, 1134, 1083, 1016, 994, 949, 896, 843, 721, 692, 658, 624. ^1^H NMR (300.1 MHz, DMSO-*d*_6_): *δ* 8.02 (d, *J* = 7.2 Hz, 1H, CH), 7.60–7.10 (m, 11H, 11CH), 6.94 (d, *J* = 7.9 Hz, 1H, CH), 6.87 (t, *J* = 7.5 Hz, 1H, CH), 6.67 (s, 1H, CH), 3.63 and 3.61 (2s, 6H, 2OCH_3_). ^13^C NMR (75.1 MHz, DMSO-*d*_6_): *δ* 153.61, 152.45, 146.11, 141.90, 141.31, 137.99, 131.70, 131.01, 129.66, 129.58, 129.04, 128.77, 127.84, 127.41, 124.15, 121.54, 119.26, 116.94, 115.58, 110.52, 60.42, 56.43. ESI–MS m/z: 536.09 [M]^+^. Anal. Calcd. for C_30_H_22_BrN_3_O_2_: C, 67.17; H, 4.13; N, 7.83.; found: C, 67.28; H, 4.29; N, 8.05%.

### *α*-Glucosidase inhibition assay

*α*-Glucosidase enzyme ((EC3.2.1.20, Saccharomyces cerevisiae, 20 U mg^−1^) and substrate (p-nitrophenyl glucopyranoside) were purchased from Sigma-Aldrich. Enzyme was prepared in potassium phosphate buffer (pH 6.8, 50 mM), as well as substituted benzo[4,5]imidazo[1,2-*c*]quinazoline **6a-c** and highly-substituted imidazo[1,2-*c*]quinazolines **11a–o** were dissolved in DMSO (10% final concentration). The various concentrations of these compounds (20 ml), enzyme solution (20 ml) and potassium phosphate buffer (135 ml) were added in the 96-well plate and incubated at 37 °C for 10 min. Afterwards, the substrate (25 ml, 4 mM) was added to the mentioned mixture and allowed to incubate at 37 °C for 20 min. Finally, the change in absorbance was measured at 405 nm by using spectrophotometer (Gen5, Power wave xs2, BioTek, America). The percentage of enzyme inhibition was calculated using Eq. (6) and IC_50_ values were obtained from non-linear regression curve using the Logit method.

6 $$\% {\text{ Inhibition }} = \, \left[ {\left( {{\text{Abs}}_{{{\text{control}}}} - {\text{ Abs}}_{{{\text{sample}}}} } \right) \, /{\text{ Abs}}_{{{\text{control}}}} } \right] \, \times {1}00$$

### Kinetic studies

The kinetic analysis was performed for the most potent derivative **11j** to reveal the inhibition mode against *α*-glucosidase. The 20 ml of enzyme solution (1U ml^−1^) was incubated with different concentrations (0, 3.1, 6.2, and 12.4 µM) of this compound for 15 min at 30 °C. Afterwards, various concentrations of substrate (p-nitrophenyl glucopyranoside, 1–10 mM) was added to measure the change of absorbance for 20 min at 405 nm by using spectrophotometer (Gen5, Power wave xs2, BioTek, America).

In the presence of a competitive inhibitor, K_m_ increases while V_max_ does not change. Michaelis–Menten saturation curve for an enzyme reaction shows the relation between the substrate concentration and reaction rate as bellow:

7 $$\frac{{\text{v}}}{{V_{{{\text{max}}}} }} = \frac{\left[ S \right]}{{K{\text{m}}_{app} + \left[ S \right]}}$$

According to Michaelis–Menten graph, Km_app_ is also defined as:

8 $$K{\text{m}}_{app} = \left( {{1} + \frac{{\left[ {\text{I}} \right]}}{{K_{{\text{I}}} }}} \right)$$

[I] is the concentration of inhibitor.

Lineweaver Burk plot that provides a useful graphical method for analysis of the Michaelis–Menten is represented as:

9 $$\frac{1}{{V_{m} }} = \frac{{K_{m} }}{{V_{max} }}\left( {{1} + \frac{{\left[ {\text{I}} \right]}}{{K_{{\text{I}}} }}} \right)\frac{1}{\left[ S \right]} + \frac{1}{{{\text{V}}_{{{\text{max}}}} }}$$

Therefore, the slope of Lineweaver Burk plot is equal to:

10 $${\text{Slope }} = \frac{{K_{m} }}{{V_{max} }}\left( {{1} + \frac{{\left[ {\text{I}} \right]}}{{K_{{\text{I}}} }}} \right)$$

The Km_app_ value is calculated by Eq. (6):

11 $$K{\text{m}}_{app} = K_{m} \left( {{1} + \frac{{\left[ {\text{I}} \right]}}{{K_{{\text{I}}} }}} \right)$$

Therefore, from replot of Km_app_ Vs. [I], Eq. (7) can be used for the calculation of K_I_^59,60^:

12 $$Km_{app} = K_{m} + \frac{{K_{m} }}{{K_{{\text{I}}} }}\left[ {\text{I}} \right]$$

### Fluorescence spectroscopy measurements

This assay was carried out for the most potent derivative **11j** to measure the fluorescence intensity. To this aim, different solutions containing different concentrations (0 to 1.0 µM) of the inhibitor and α-glucosidase (3 ml, 0.1 U ml^−1^) were held for 10 min to equilibrate before measurements. Moreover, the fluorescence of the buffer containing compound **11j** in the absence of the enzyme were subtracted as the background fluorescence. Afterwards, at the excitation wavelength of 280 nm, the fluorescence emission spectra were measured from 300 to 450 nm using a Synergy HTX multi-mode reader (Biotek Instruments, Winooski, VT, USA) equipped with a 1.0 cm quartz cell holder^61^.

### Molecular docking studies

Molecular docking study using AutoDock4 and Auto Dock Tools (version 1.5.6) was performed on compounds **11a–o** and **6a–c** to elucidate the patterns of their interactions in the active site of the human acid-alpha-glucosidase (PDB ID: 5NN8). Receptor was prepared by removing water molecules and computing Kollman charges with BIOVIA Discovery Studio visualizer and Auto Dock Tools. To validate the docking procedure, redocking process was performed with acarbose as standard ligand, and RMSD value of 1.57 was achieved. The redocked ligand identified similar binding pose to original co-crystalized position downloaded from RCSB database (5NN8). Acarbose was extracted from the PDB file using BIOVIA Discovery Studio visualizer and saved as a separate PDB file. A possible grid box was determined using Auto Dock Tools (version 1.5.6). Furthermore, genetic algorithm was selected as the searching parameter. This procedure was carried out for different potential grid coordinates. Finally, the best grid coordinates were determined by comparing RMSD values.

Afterwards, ligands **11** and **6** were prepared by adding Gasteiger Charges using Auto Dock Tools, and the docking procedure was conducted with 100 genetic algorithm runs using AutoDock4 and AutoGrid4. The interactions were visualized by PLIP online service^62^ and PyMOL Molecular Graphics System, Version 2.5.2 Schrödinger, LLC.

### Statistical analysis

Statistical analysis for all compounds **6a–c** and **11a–o** was performed using SigmaPlot version 14 (Systat-Software, USA). The experiments were replicated three times under the same conditions. Data for each compound was used as mean ± SD in T test method. A p-value lower than 0.05 was regarded as indicative of statistical significance.

### Supplementary Information

Supplementary Information.

## Data Availability

The authors confirm that the data supporting the finding of this study are available within the manuscript and supplementary file.
