# Supplementary material for: Imidazo[1,2-c]quinazolines as a novel and potent scaffold of α-glucosidase inhibitors: design, synthesis, biological evaluations, and in silico studies
Source: Sci Rep. 2023 Sep 21;13:15672. doi: 10.1038/s41598-023-42549-5 (PMC10514295; doi:10.1038/s41598-023-42549-5)

Imidazo[1,2-*c*]quinazolines as a novel and potent scaffold of *α*-glucosidase inhibitors: design, synthesis, biological evaluations, and *in silico* studies

Fariba Peytam [1], Faezeh sadat Hosseini [2], Malak Hekmati [2], Bahareh Bayati [3], Mahdis Sadeghi Moghadam [3], Zahra Emamgholipour [3], Loghman Firoozpour [3], Somayeh Mojtabavi [4], Mohammad Ali Faramarzi [4], Seyed Esmaeil Sadat-Ebrahimi [3], Maliheh Barazandeh Tehrani [3], Alireza Foroumadi [1,3,*]

[1] Drug Design and Development Research Center, The Institute of Pharmaceutical Sciences (TIPS), Tehran University of Medical Sciences, Tehran, Iran.

[2] Department of Organic Chemistry, Faculty of Pharmaceutical Chemistry, Tehran Medical Sciences, Islamic Azad University, Tehran, Iran.

[3] Department of Medicinal Chemistry, Faculty of Pharmacy, Tehran University of Medical Sciences, Tehran, Iran.

[4] Department of Pharmaceutical Biotechnology, Faculty of Pharmacy, Tehran University of Medical Sciences, Tehran, Iran.

Corresponding author: Prof. Alireza Foroumadi (aforoumadi@yahoo.com)

^{1}H NMR spectrum of 6-phenylbenzo[4,5]imidazo[1,2-*c*]quinazoline **6a**

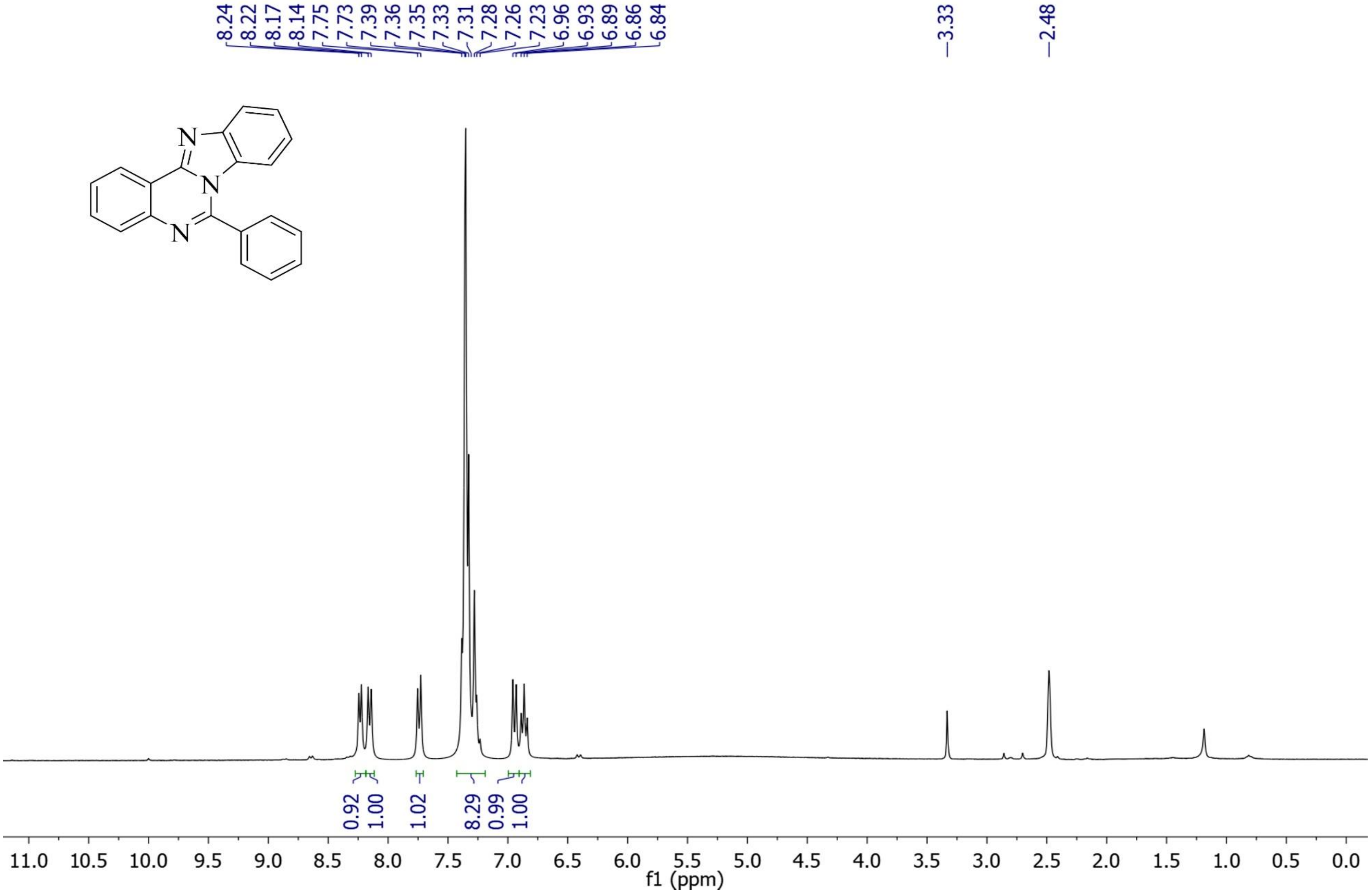

^{13}C NMR spectrum of 6-phenylbenzo[4,5]imidazo[1,2-*c*]quinazoline **6a**

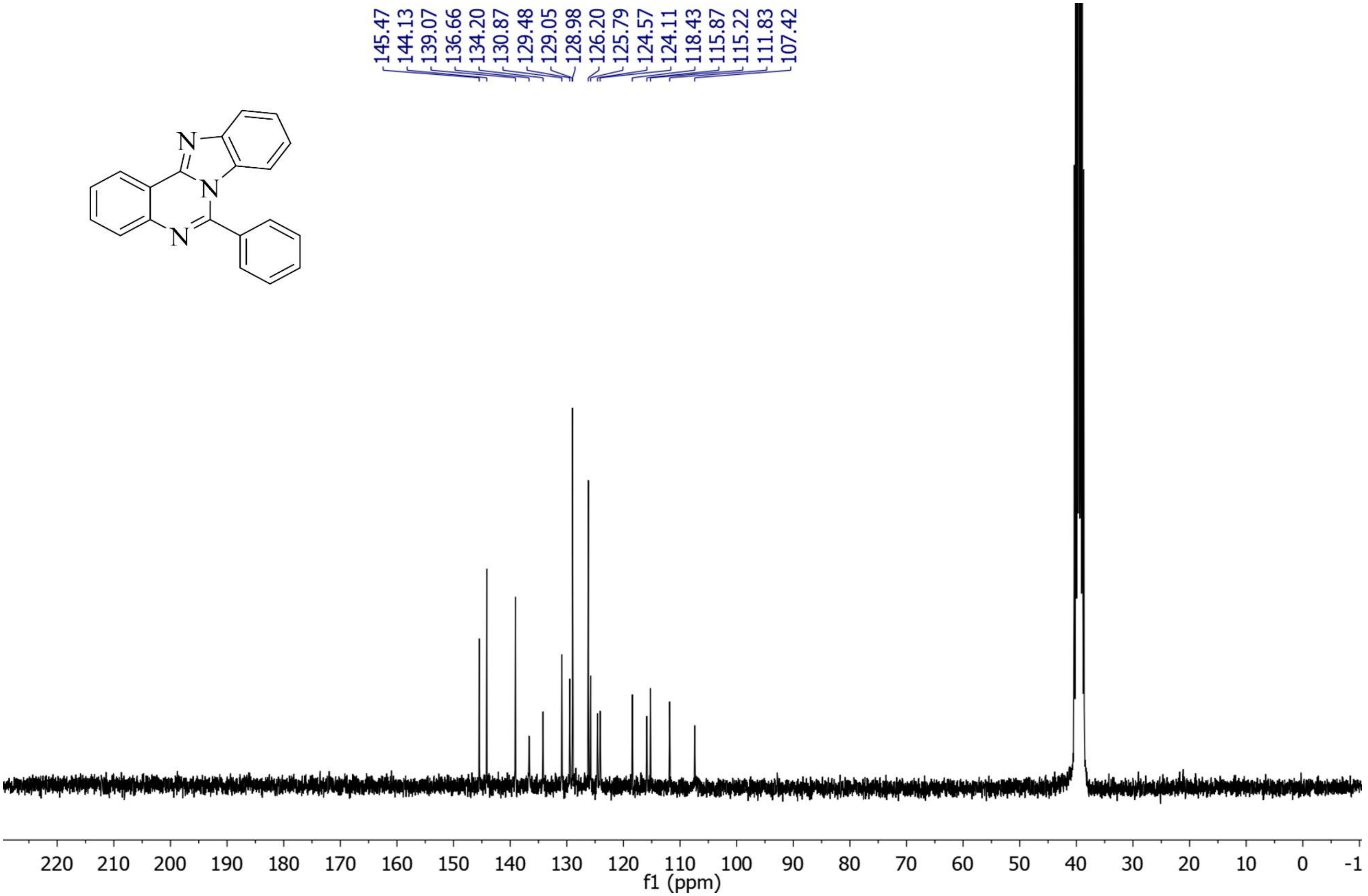

^{1}H NMR spectrum of 6-(4-chlorophenyl)benzo[4,5]imidazo[1,2-*c*]quinazoline **6b**

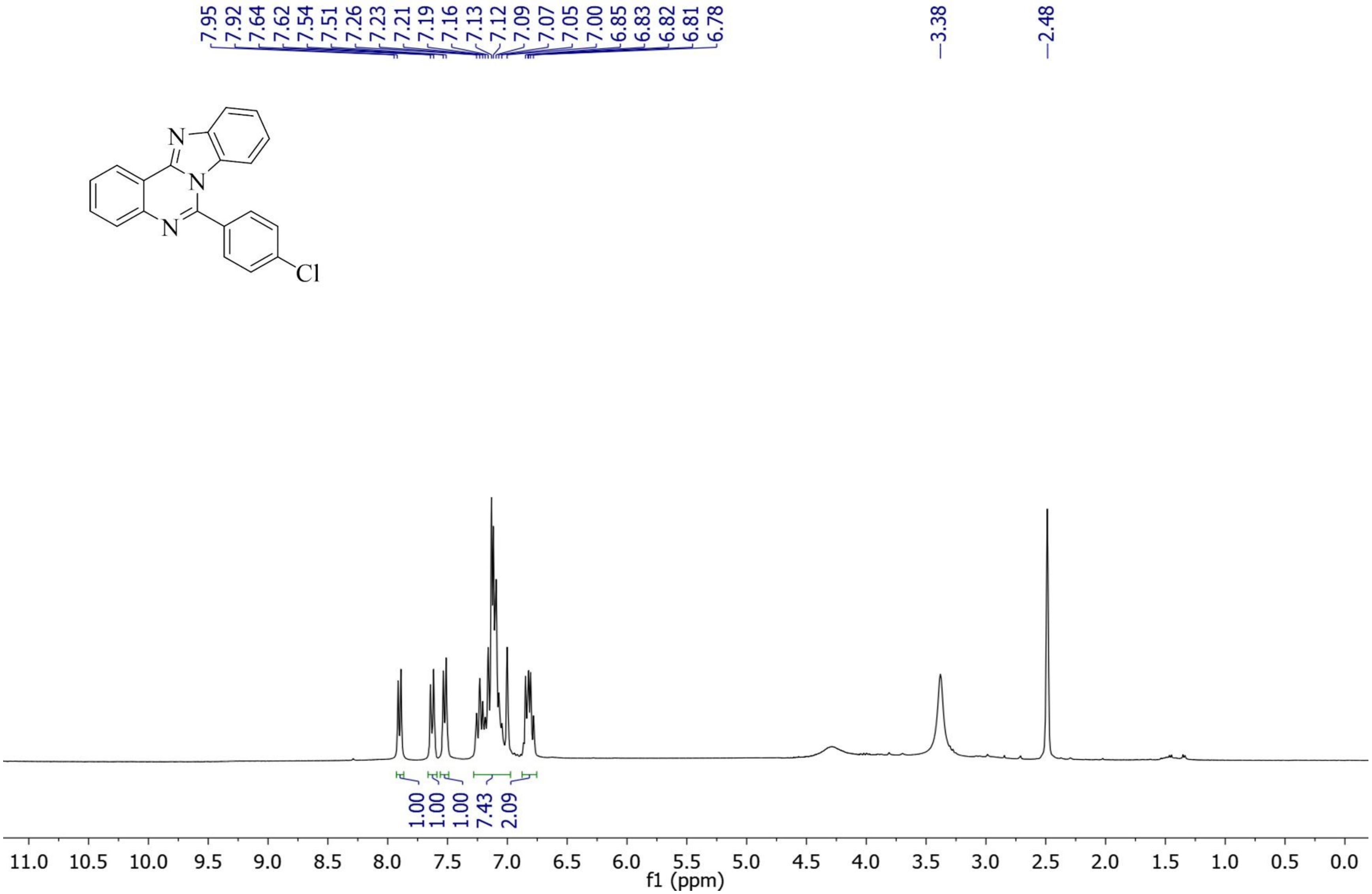

^{13}C NMR spectrum of 6-(4-chlorophenyl)benzo[4,5]imidazo[1,2-*c*]quinazoline **6b**

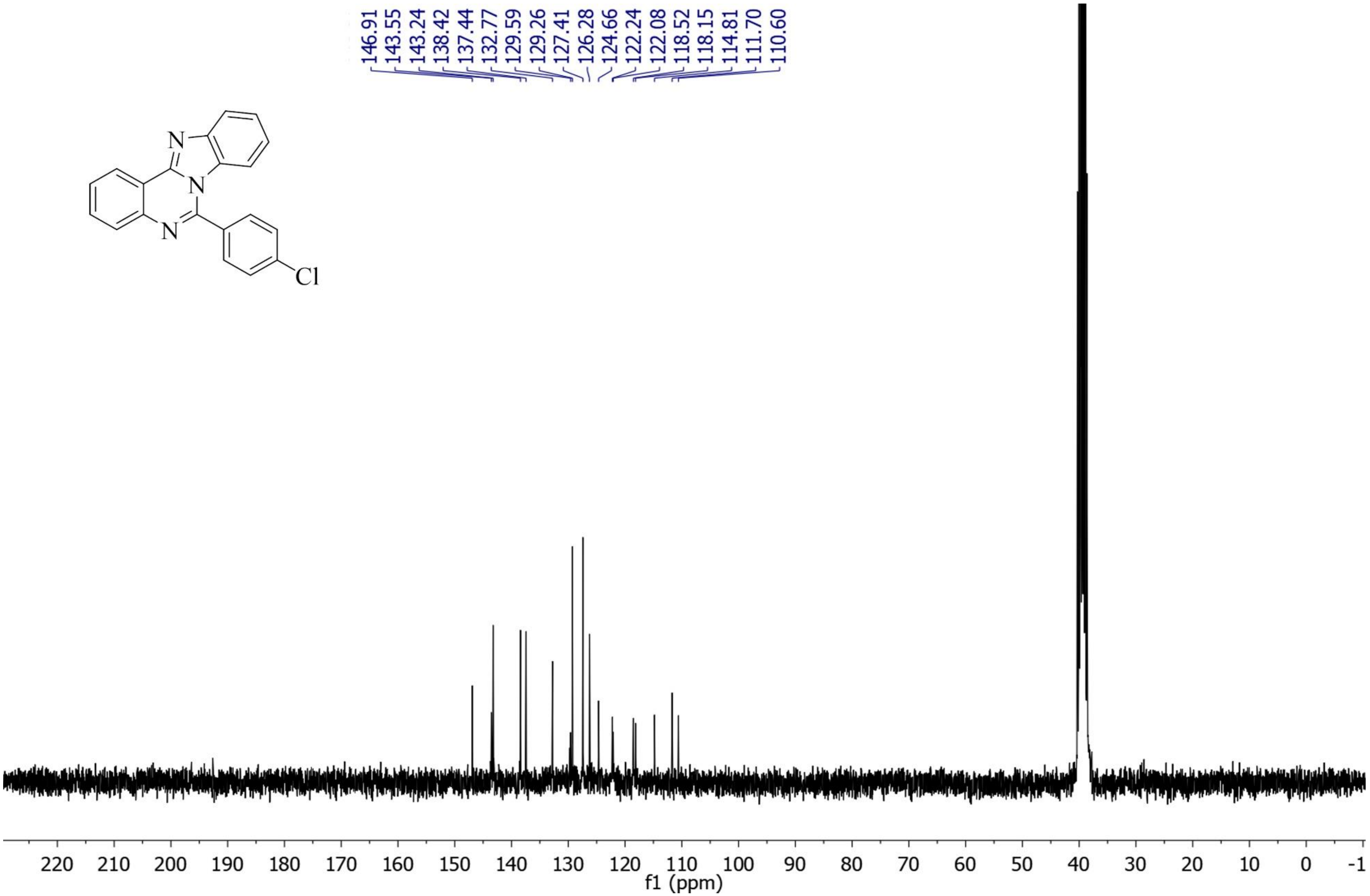

^{1}H NMR spectrum of 6-(4-methoxyphenyl)benzo[4,5]imidazo[1,2-*c*]quinazoline **6c**

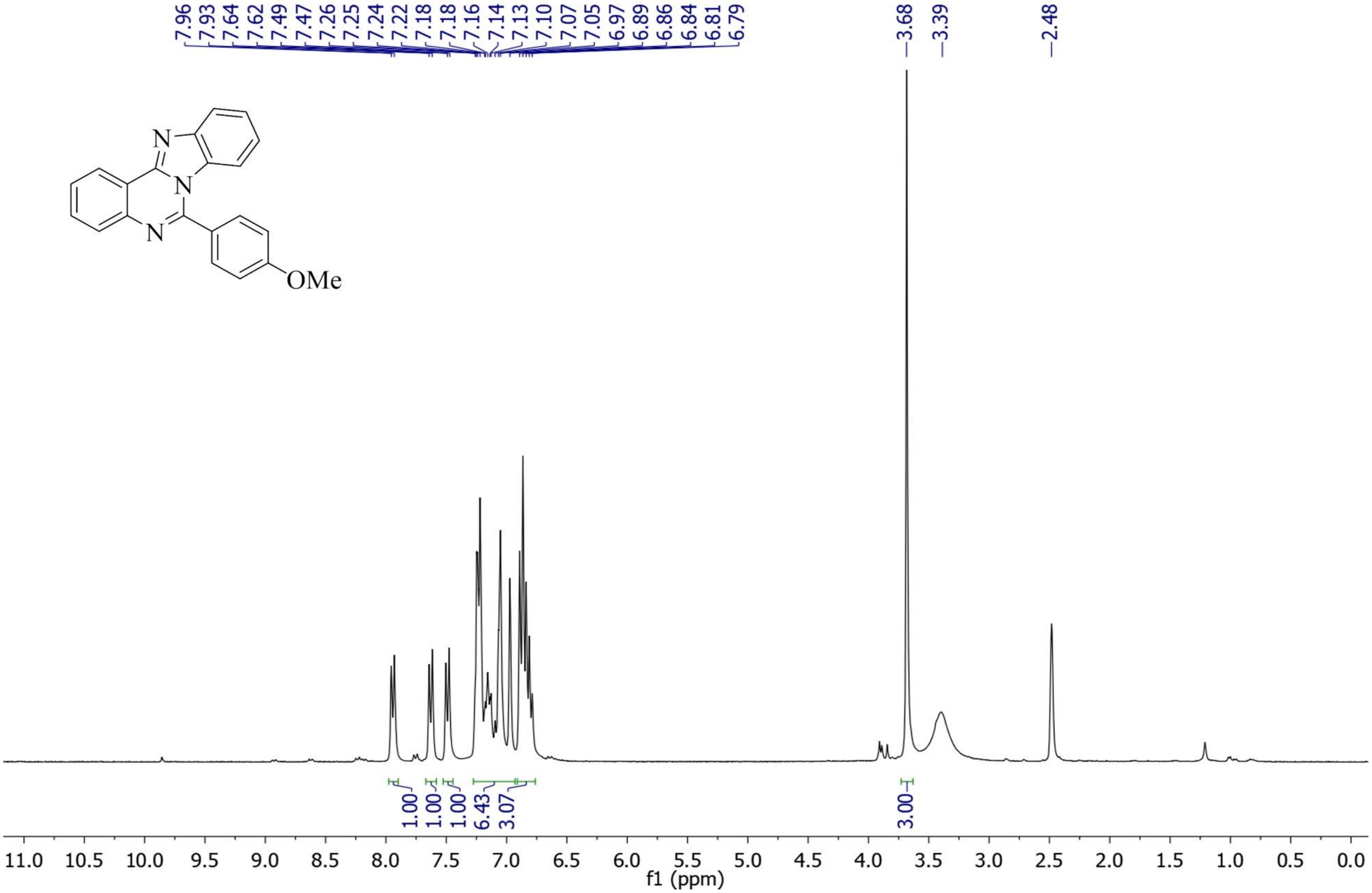

^{13}C NMR spectrum of 6-(4-methoxyphenyl)benzo[4,5]imidazo[1,2-*c*]quinazoline **6c**

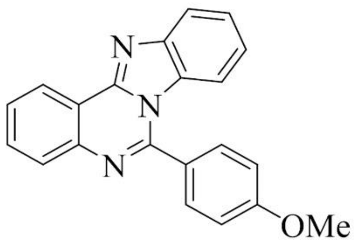

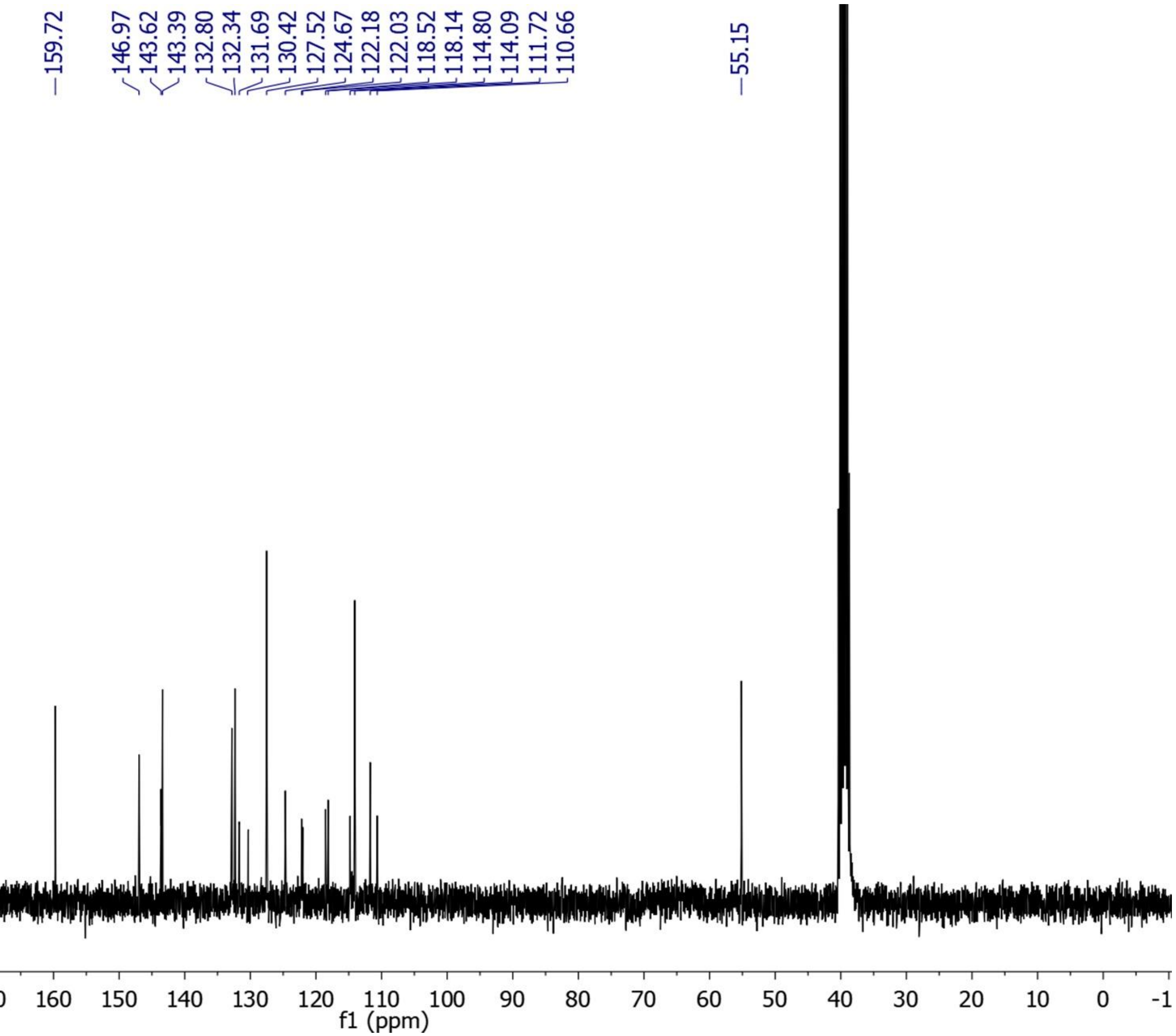

^{1}H NMR spectrum of 2,3,5-triphenylimidazo[1,2-*c*]quinazoline **11a**

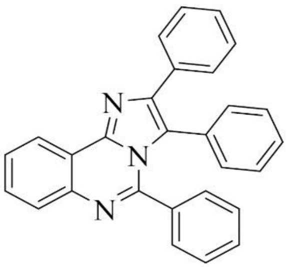

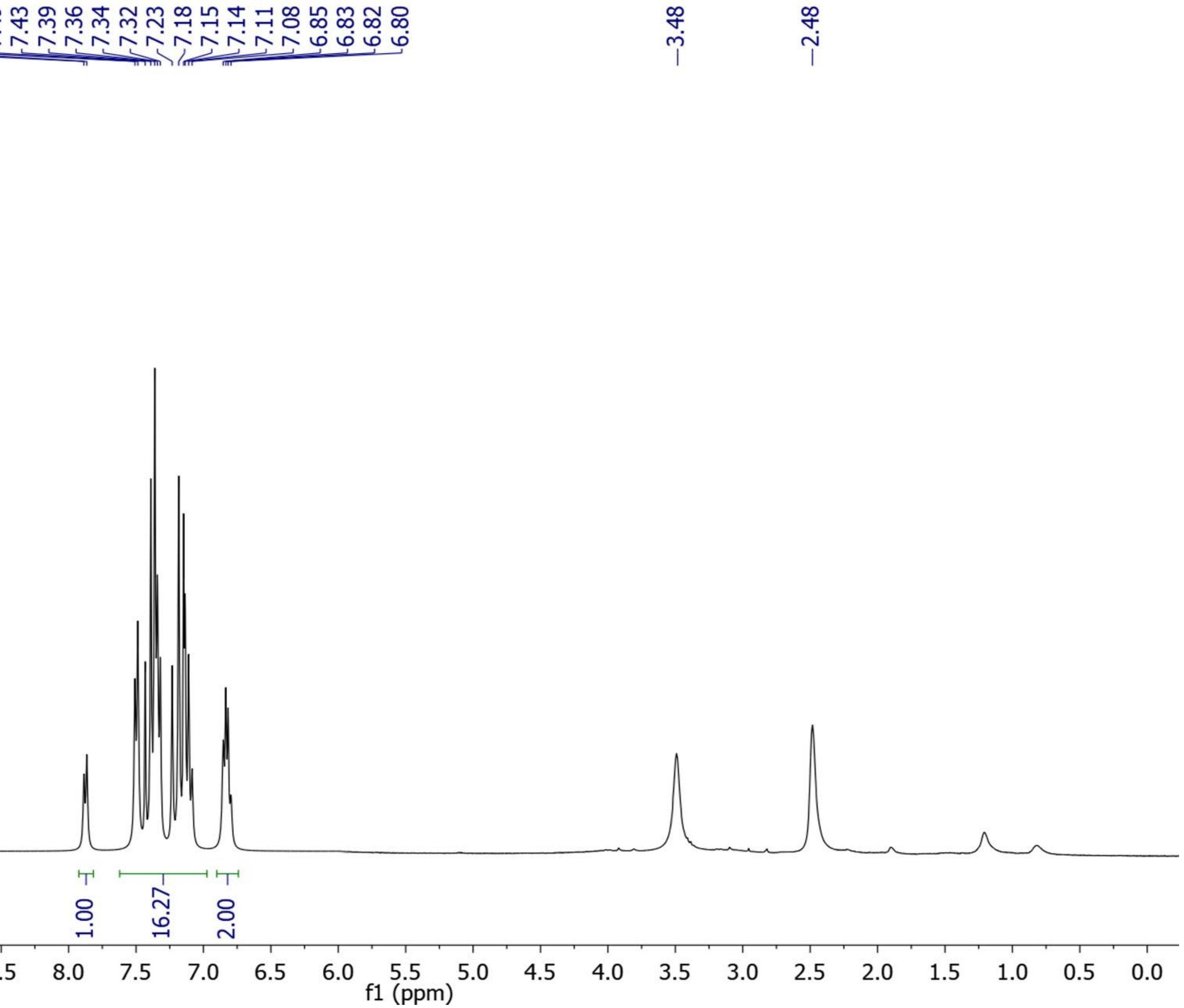

^{13}C NMR spectrum of 2,3,5-triphenylimidazo[1,2-*c*]quinazoline **11a**

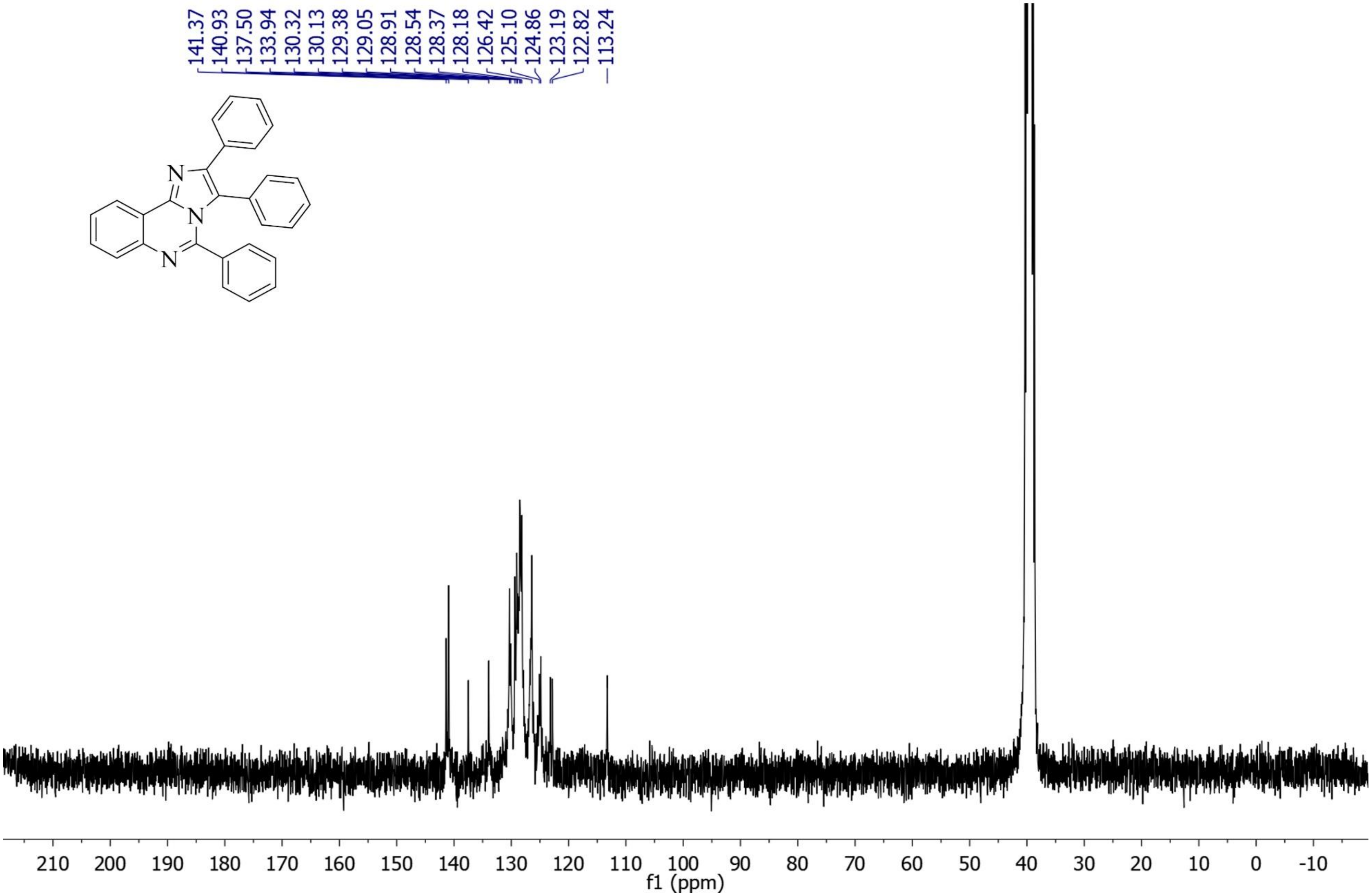

^{1}H NMR spectrum of 5-(4-chlorophenyl)-2,3-diphenylimidazo[1,2-*c*]quinazoline **11b**

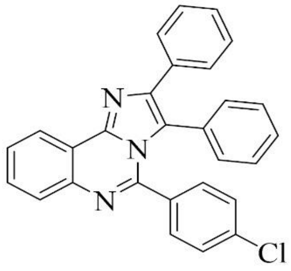

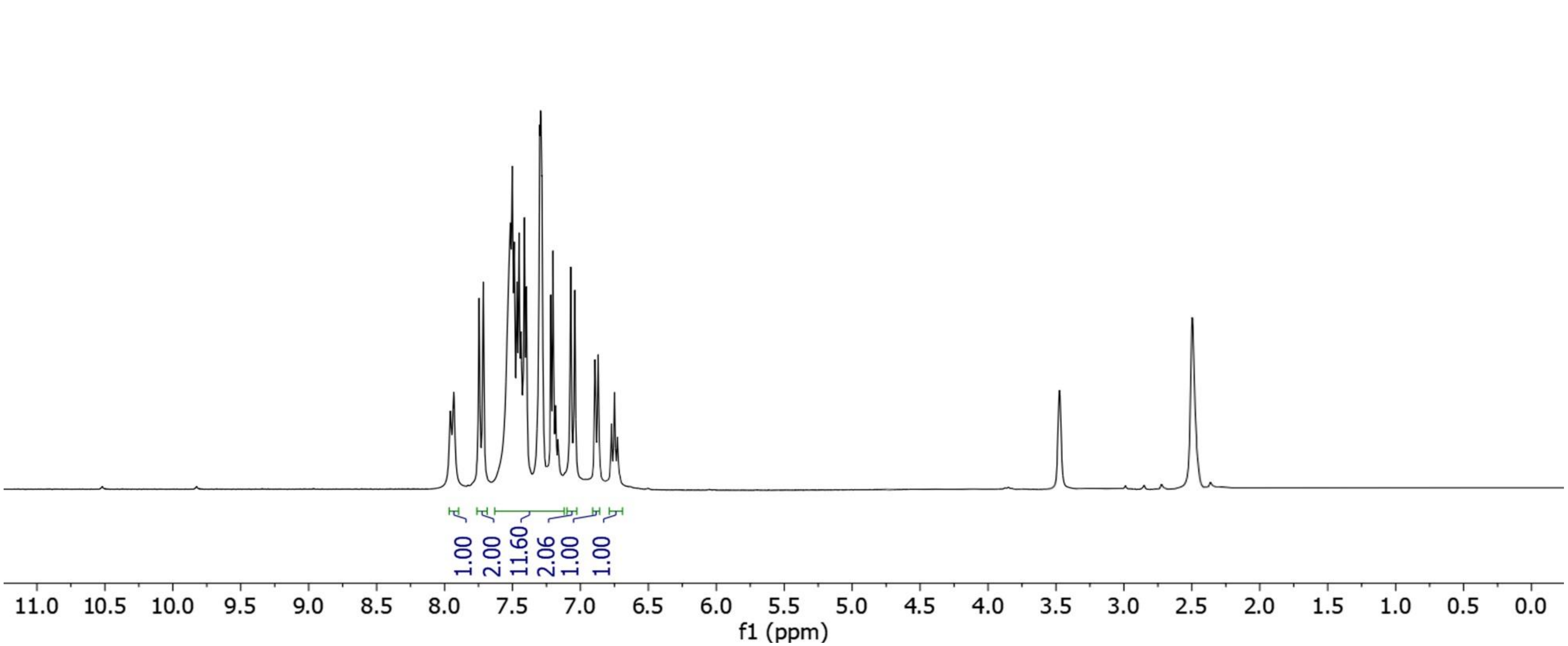

^{13}C NMR spectrum of 5-(4-chlorophenyl)-2,3-diphenylimidazo[1,2-*c*]quinazoline **11b**

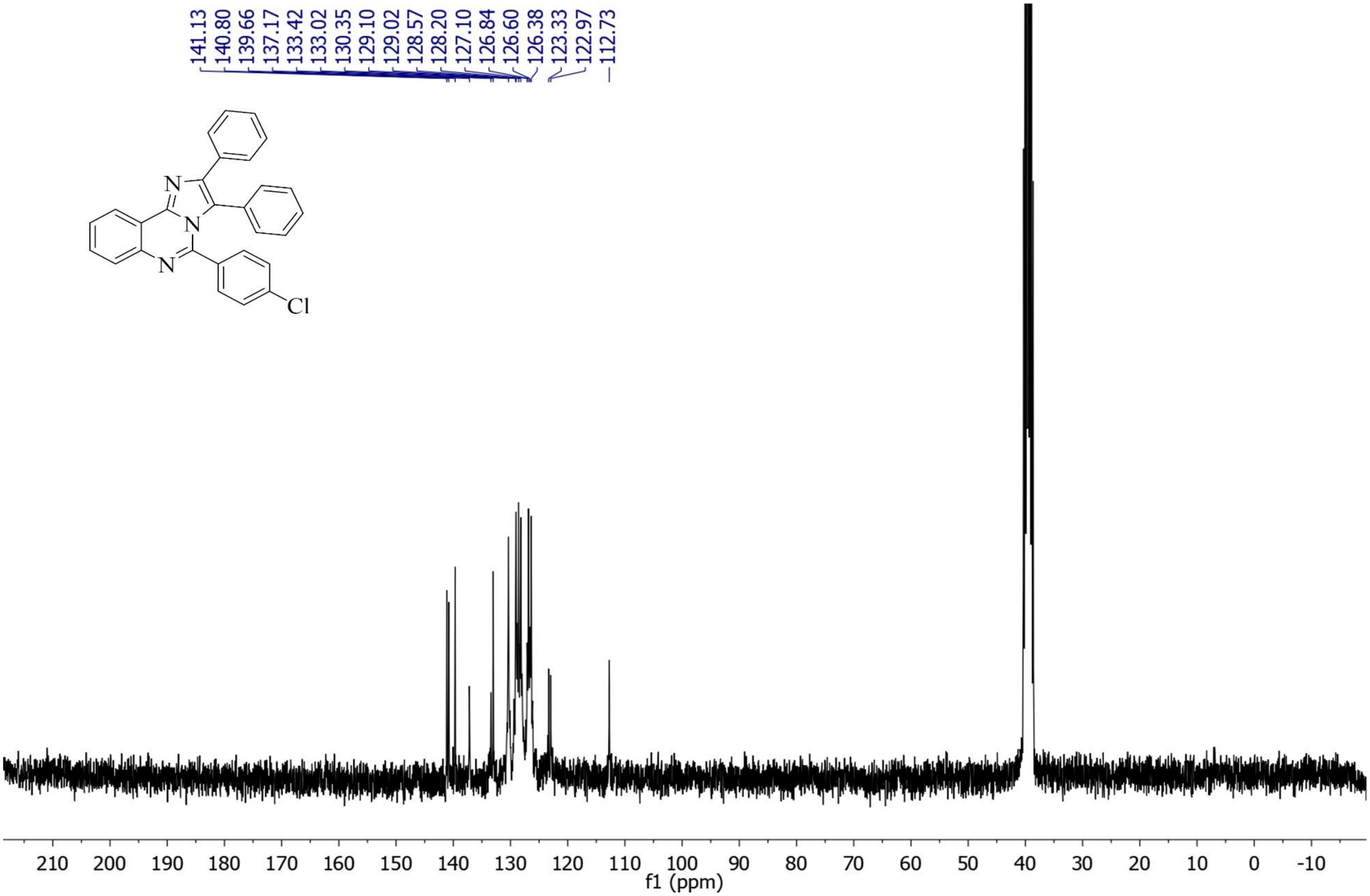

^{1}H NMR spectrum of 5-(3-chlorophenyl)-2,3-diphenylimidazo[1,2-*c*]quinazoline **11c**

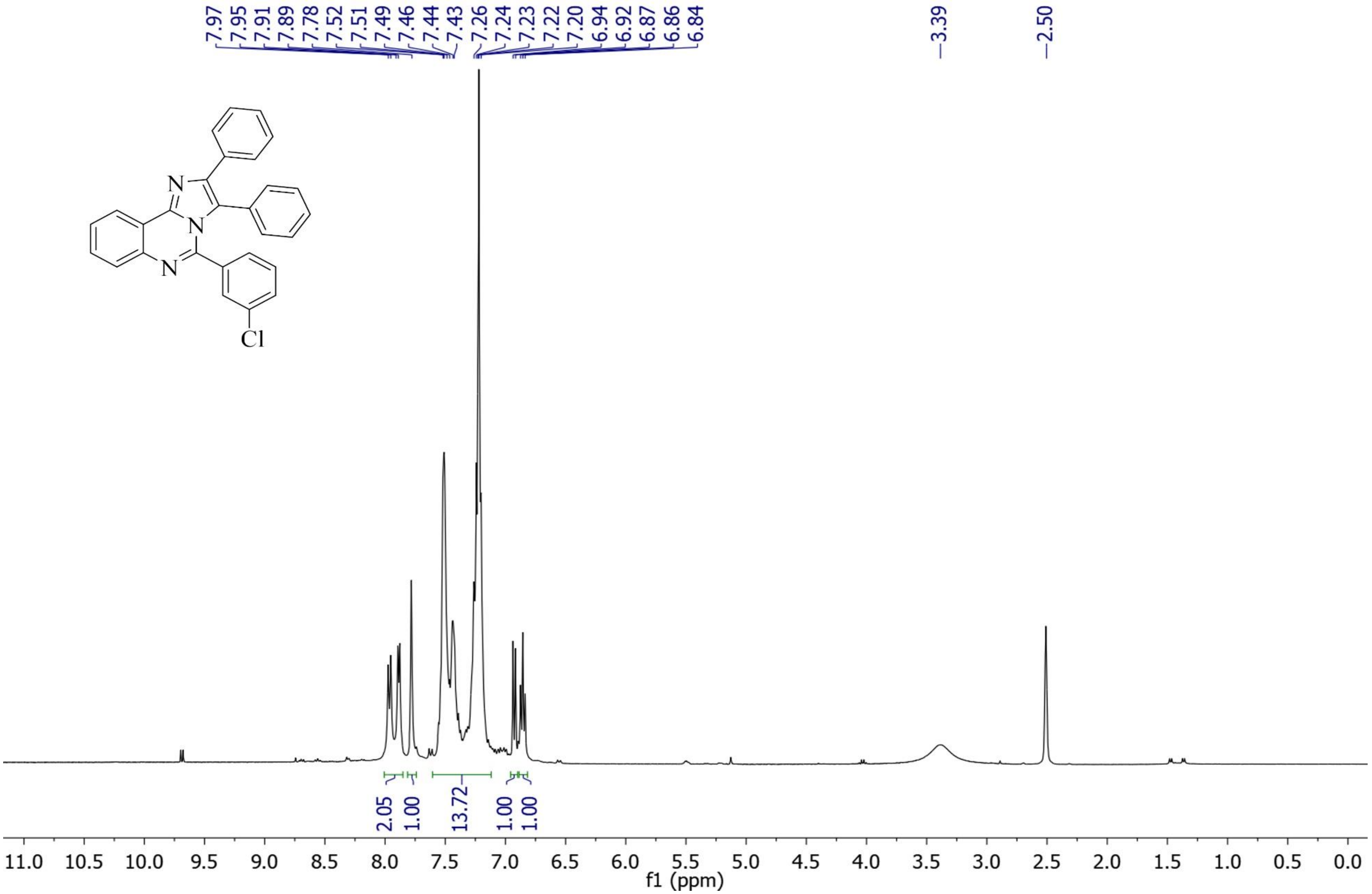

^{13}C NMR spectrum of 5-(3-chlorophenyl)-2,3-diphenylimidazo[1,2-*c*]quinazoline **11c**

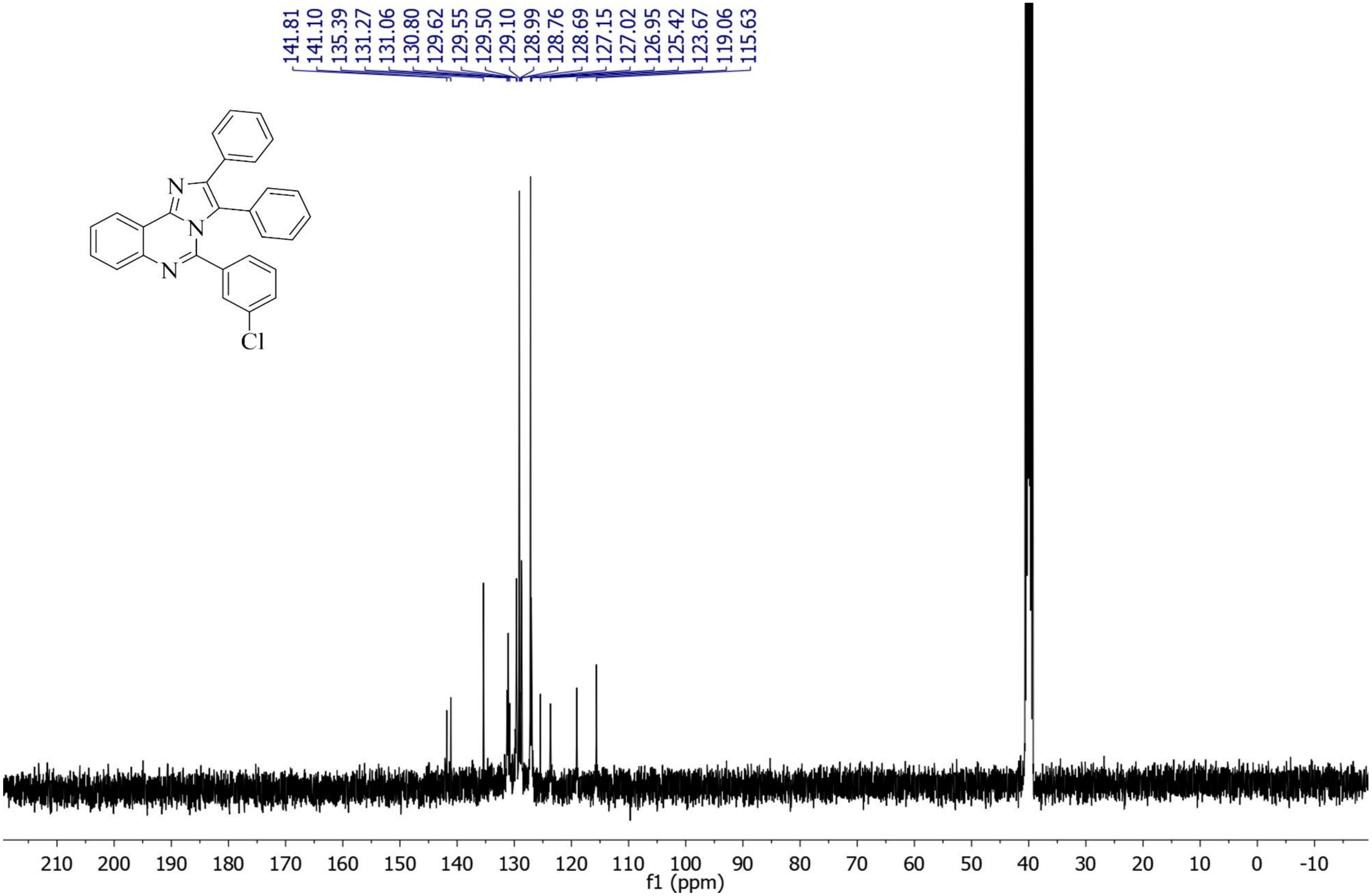

^{1}H NMR spectrum of 5-(2-chlorophenyl)-2,3-diphenylimidazo[1,2-*c*]quinazoline **11d**

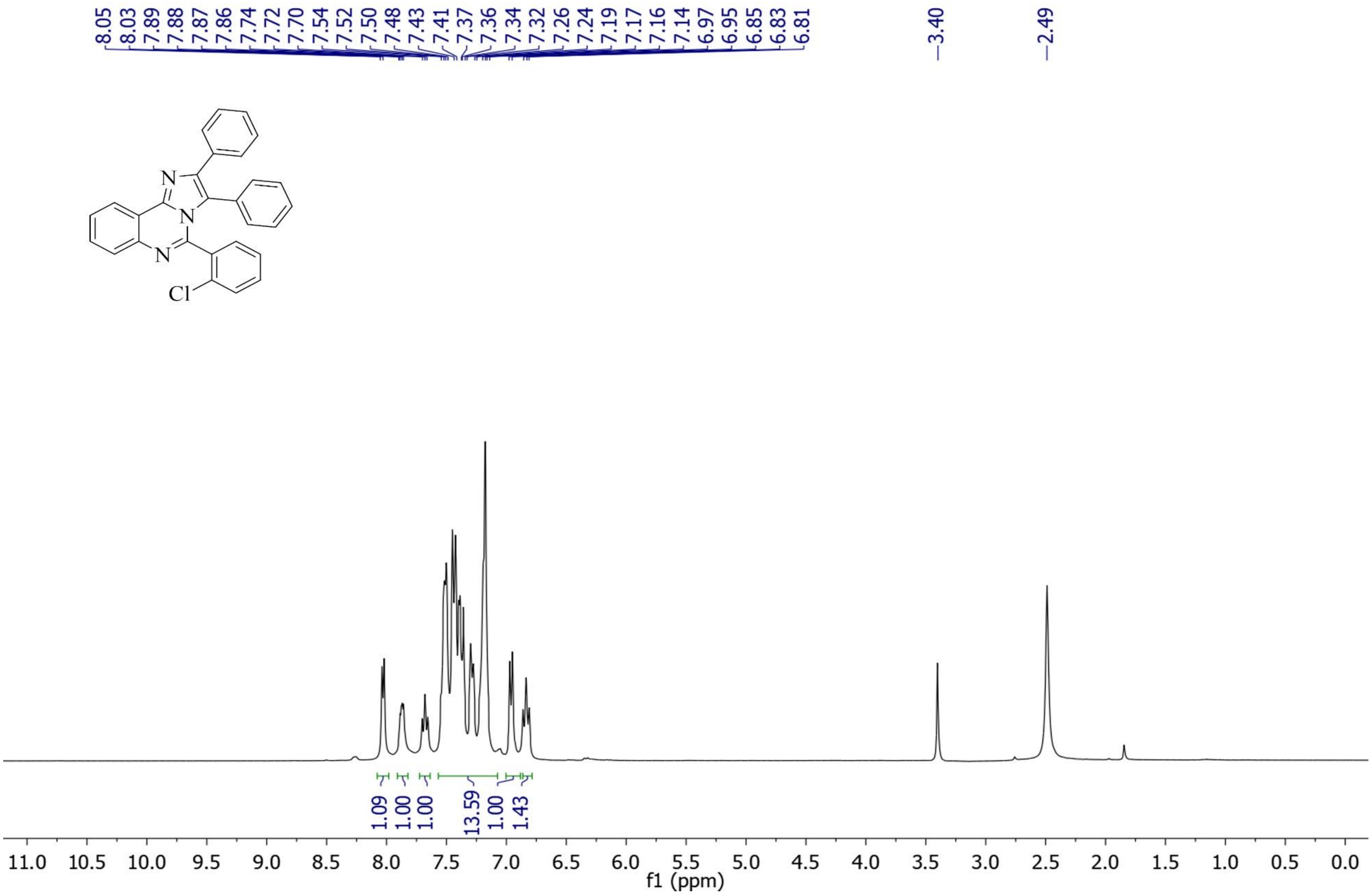

^{13}C NMR spectrum of 5-(2-chlorophenyl)-2,3-diphenylimidazo[1,2-*c*]quinazoline **11d**

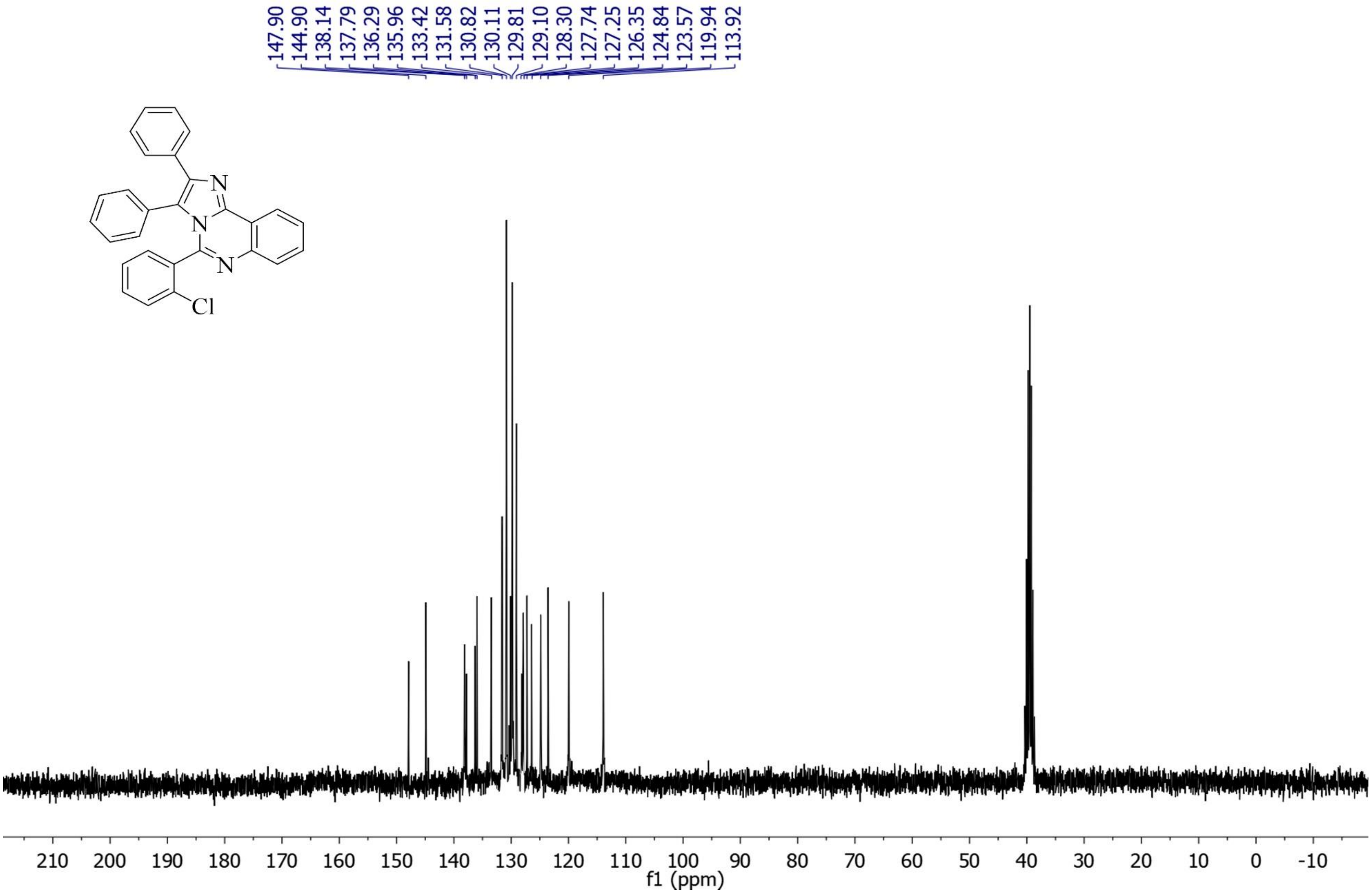

^{1}H NMR spectrum of 5-(4-methylphenyl)-2,3-diphenylimidazo[1,2-*c*]quinazoline **11e**

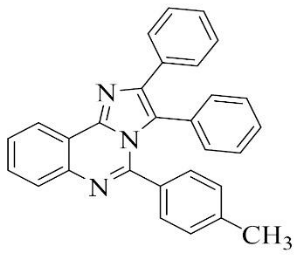

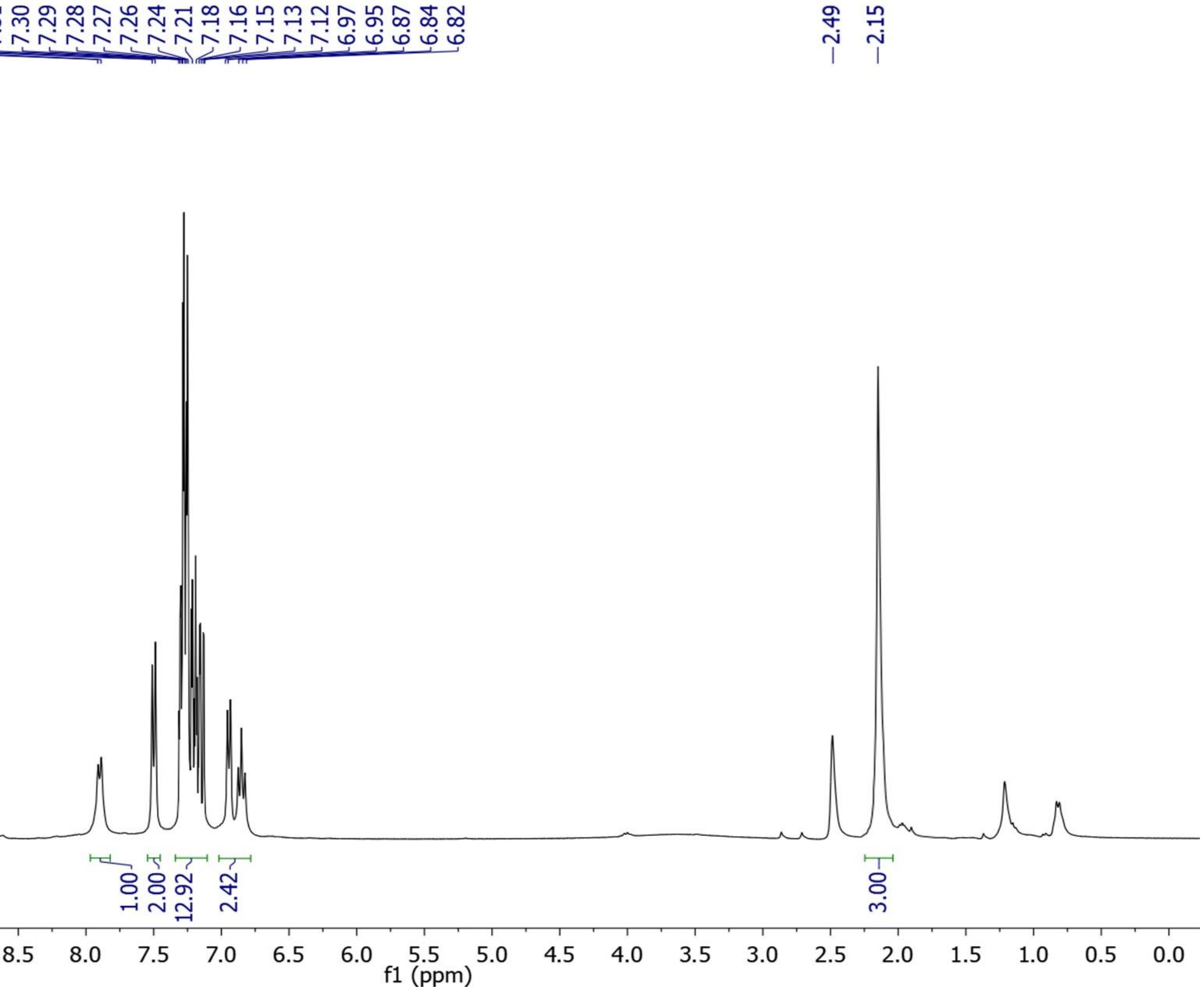

^{13}C NMR spectrum of 5-(4-methylphenyl)-2,3-diphenylimidazo[1,2-*c*]quinazoline **11e**

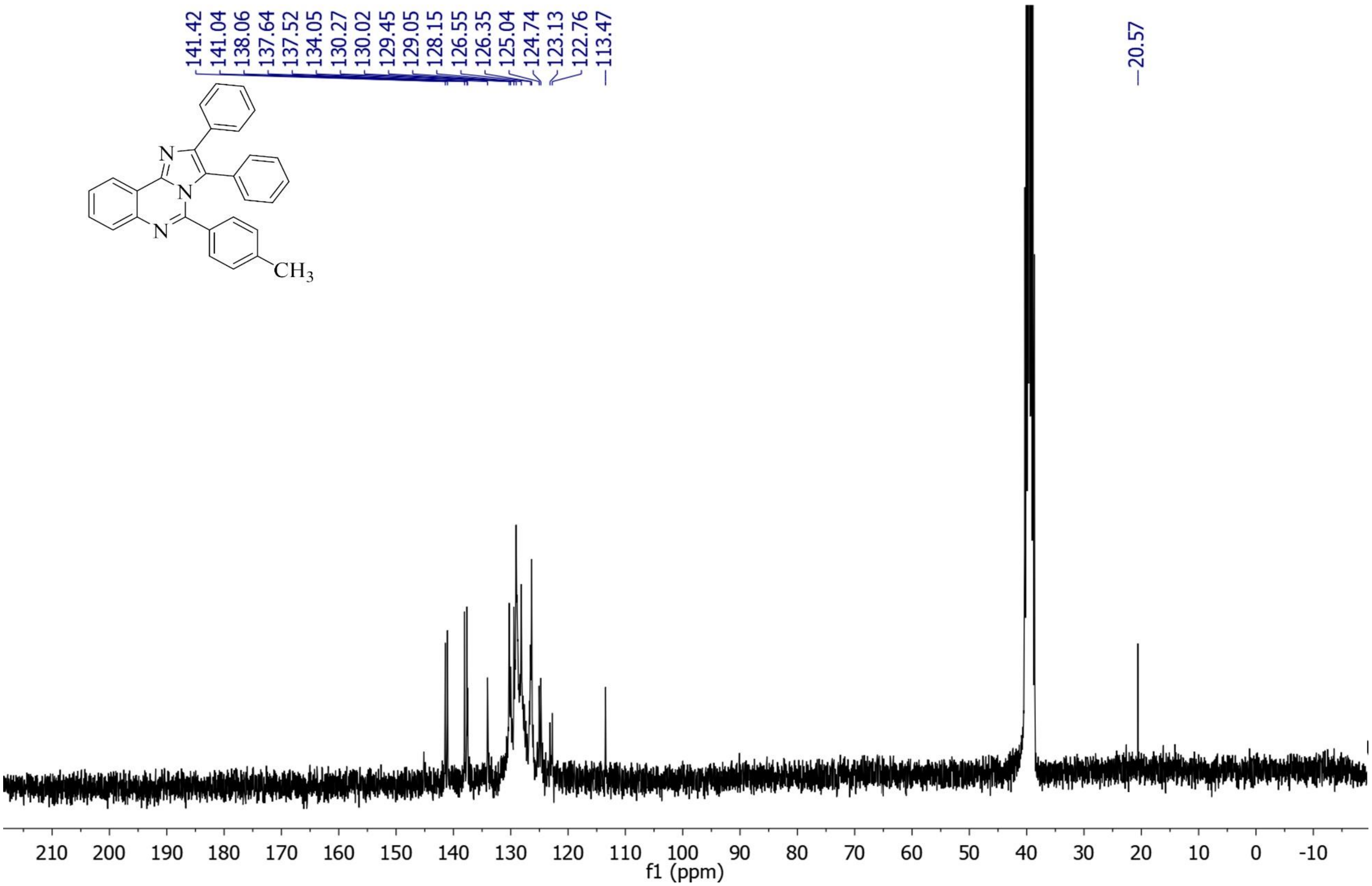

^{1}H NMR spectrum of 4-(2,3-diphenylimidazo[1,2-*c*]quinazolin-5-yl)-*N*,*N*-dimethylaniline **11f**

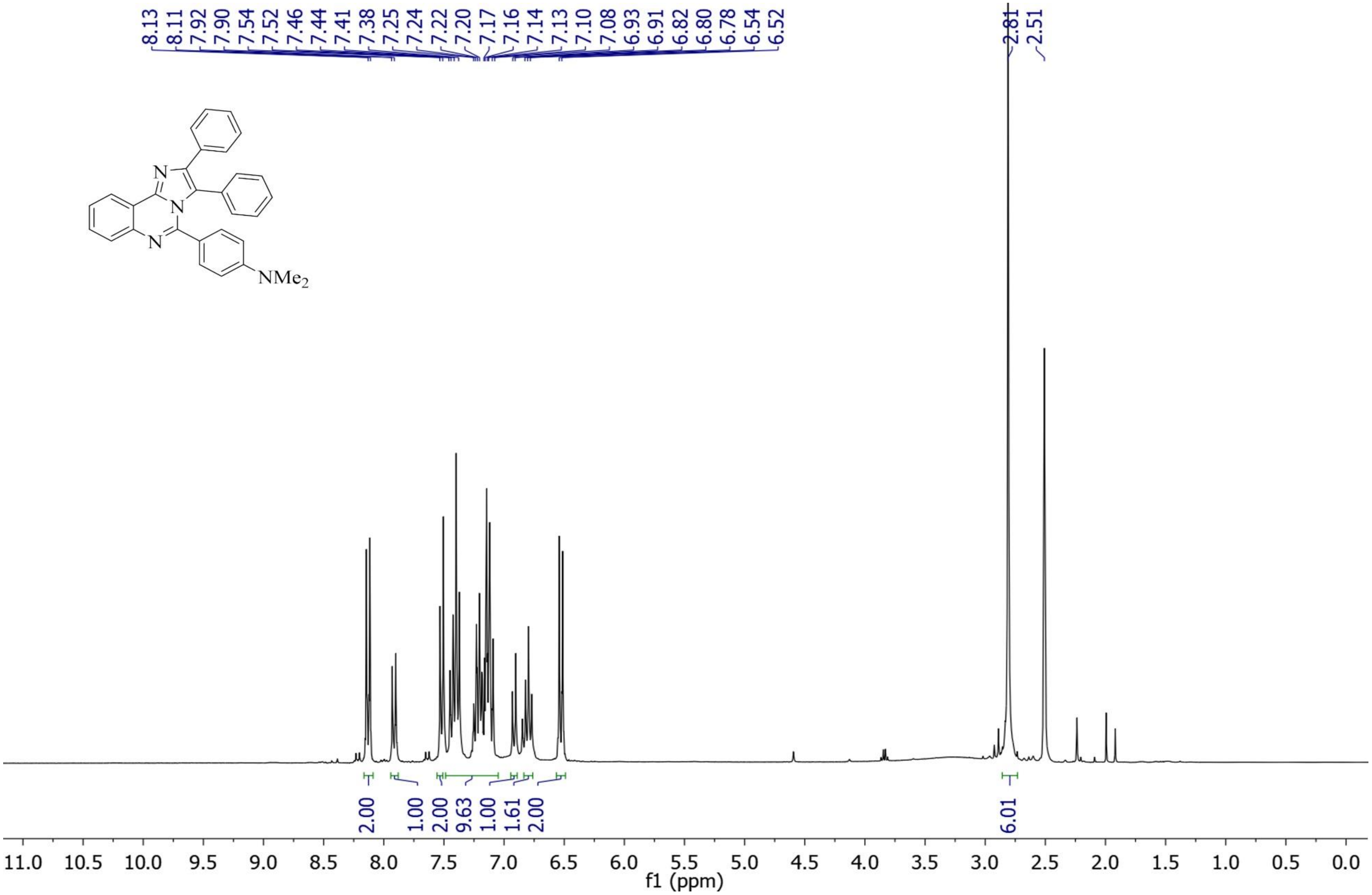

^{13}C NMR spectrum of 4-(2,3-diphenylimidazo[1,2-*c*]quinazolin-5-yl)-*N*,*N*-dimethylaniline **11f**

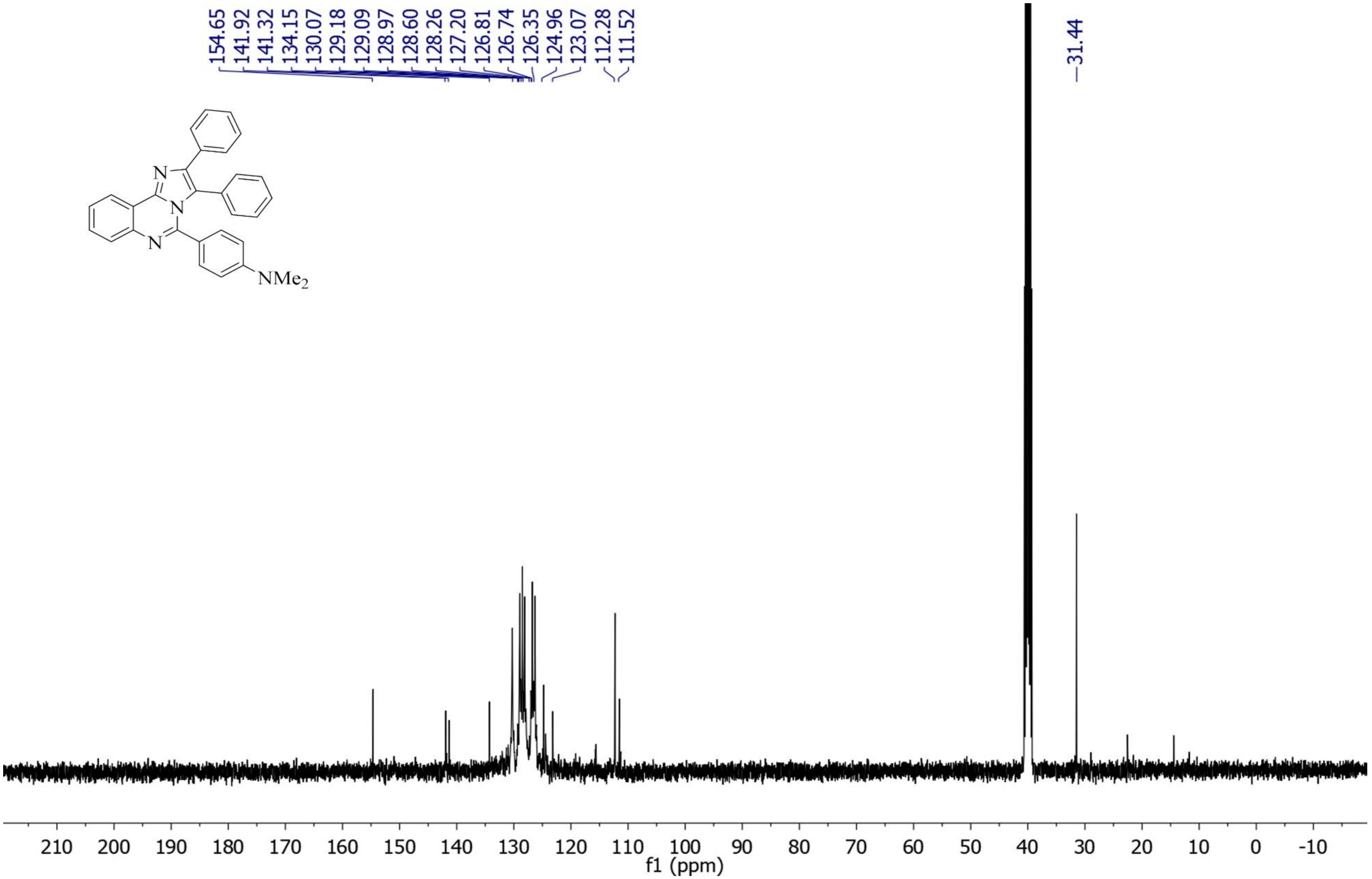

^{1}H NMR spectrum of 5-(4-methoxyphenyl)-2,3-diphenylimidazo[1,2-*c*]quinazoline **11g**

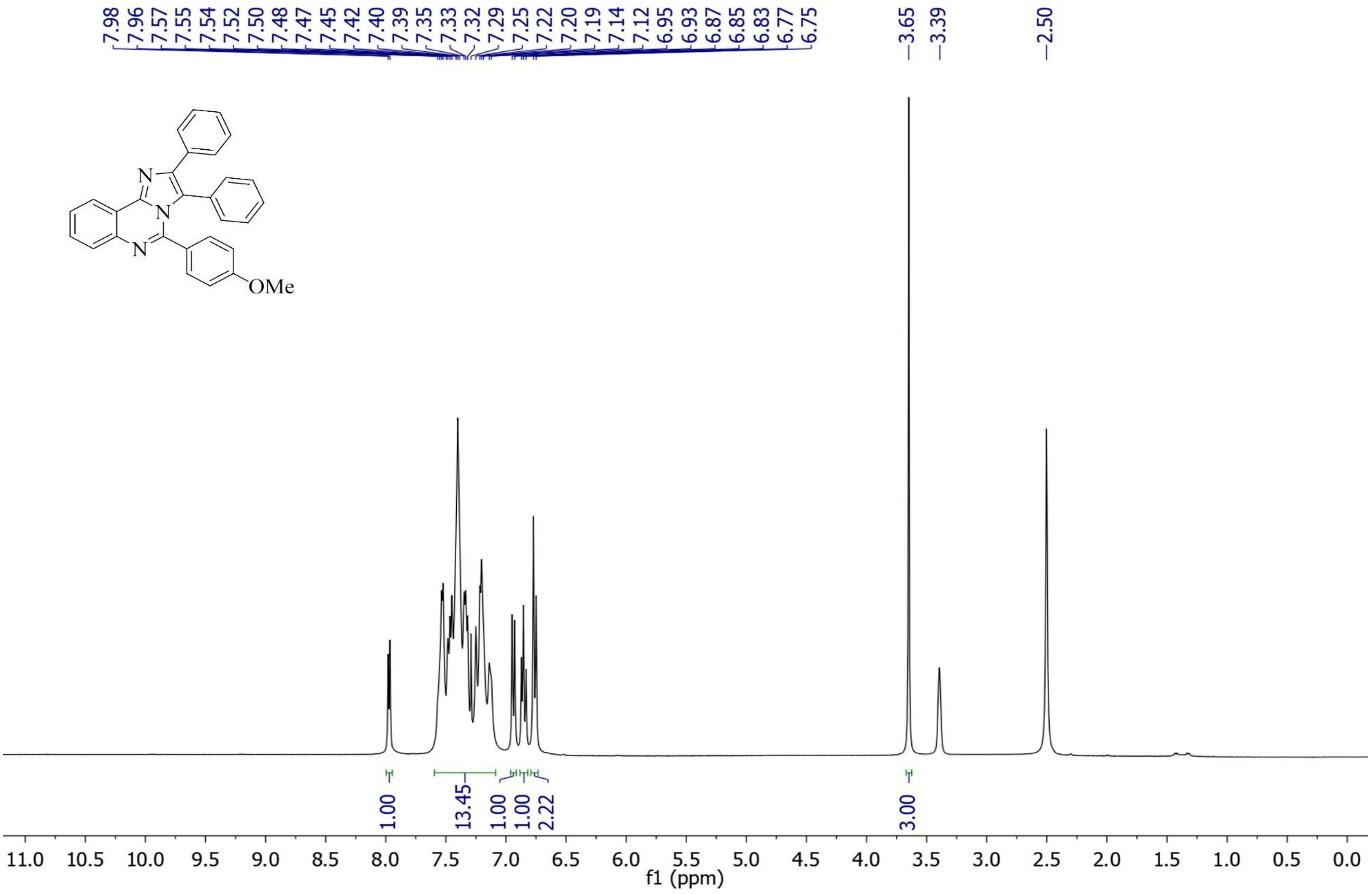

^{13}C NMR spectrum of 5-(4-methoxyphenyl)-2,3-diphenylimidazo[1,2-*c*]quinazoline **11g**

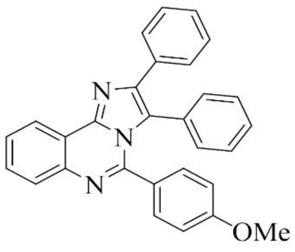

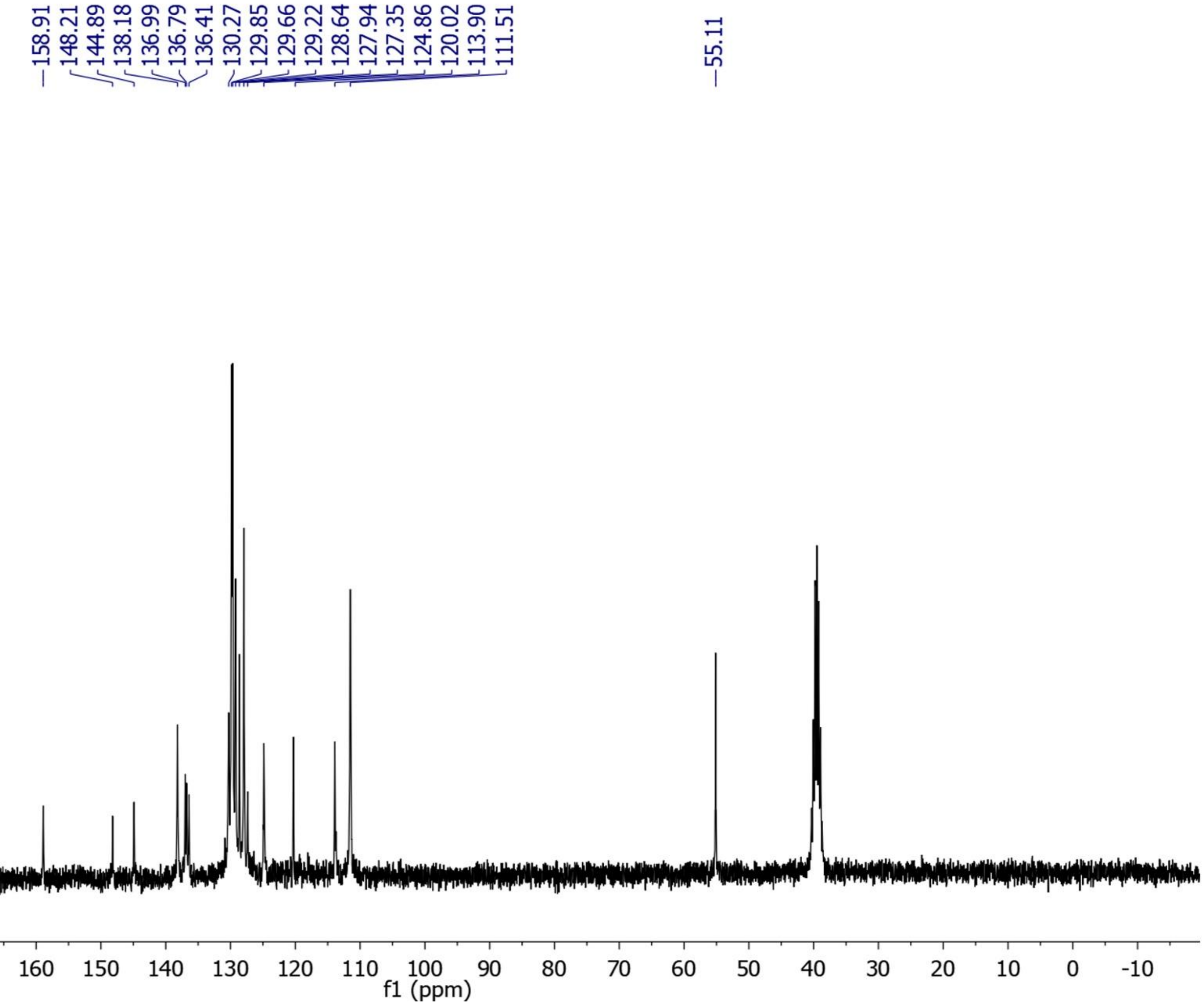

^{1}H NMR spectrum of 5-(3-methoxyphenyl)-2,3-diphenylimidazo[1,2-*c*]quinazoline **11h**

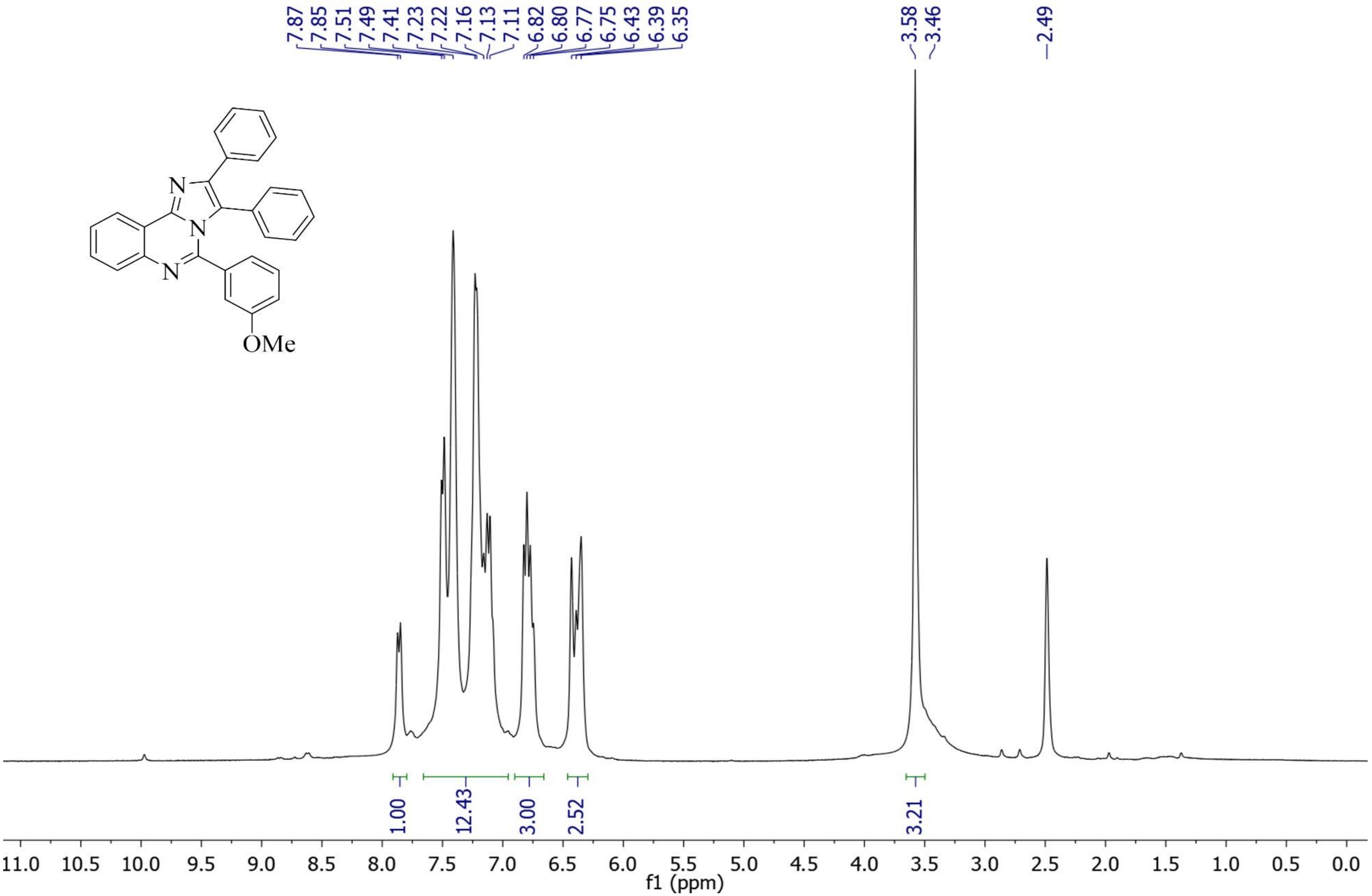

^{13}C NMR spectrum of 5-(3-methoxyphenyl)-2,3-diphenylimidazo[1,2-*c*]quinazoline **11h**

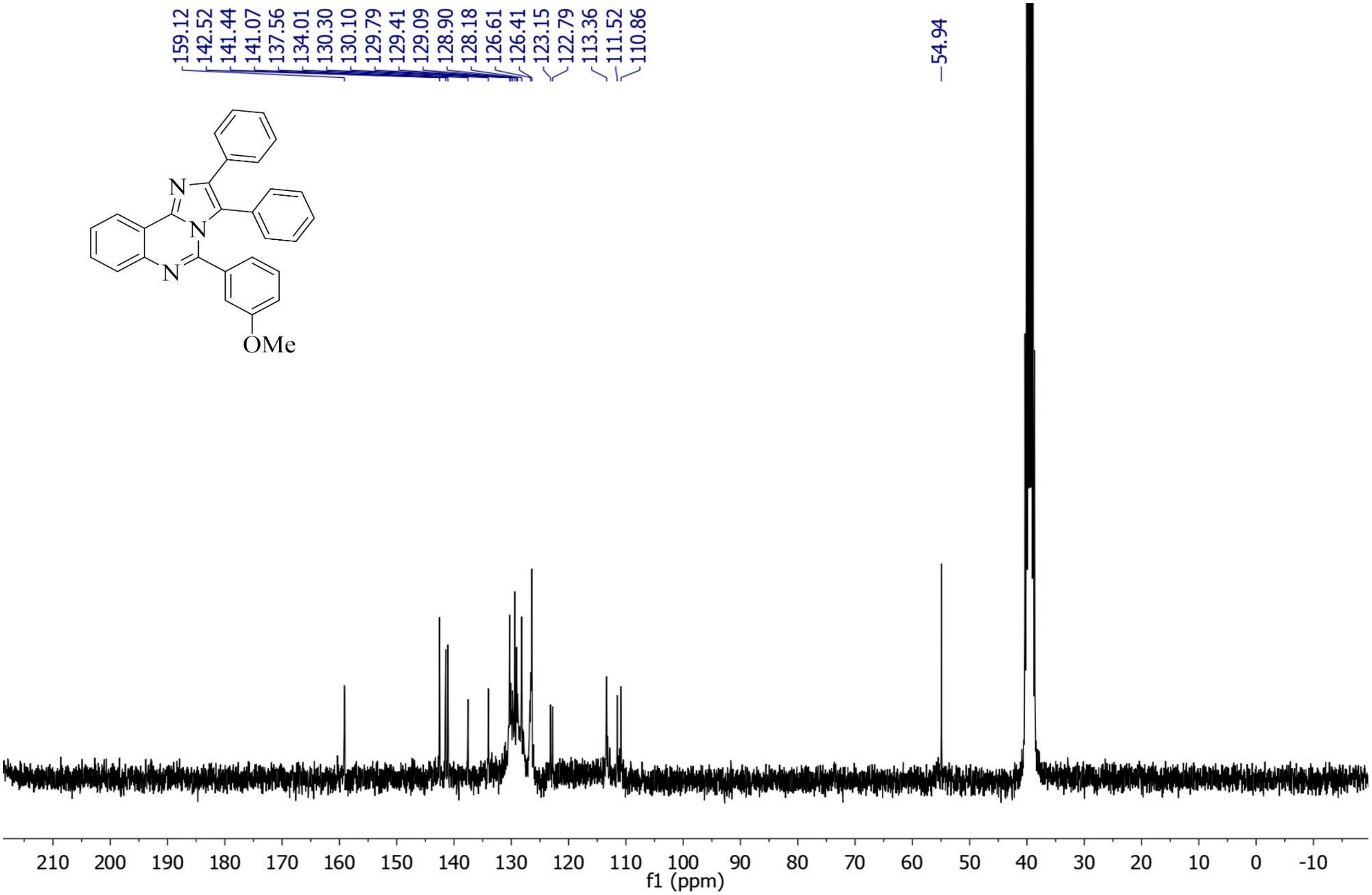

^{1}H NMR spectrum of 5-(2-methoxyphenyl)-2,3-diphenylimidazo[1,2-*c*]quinazoline **11i**

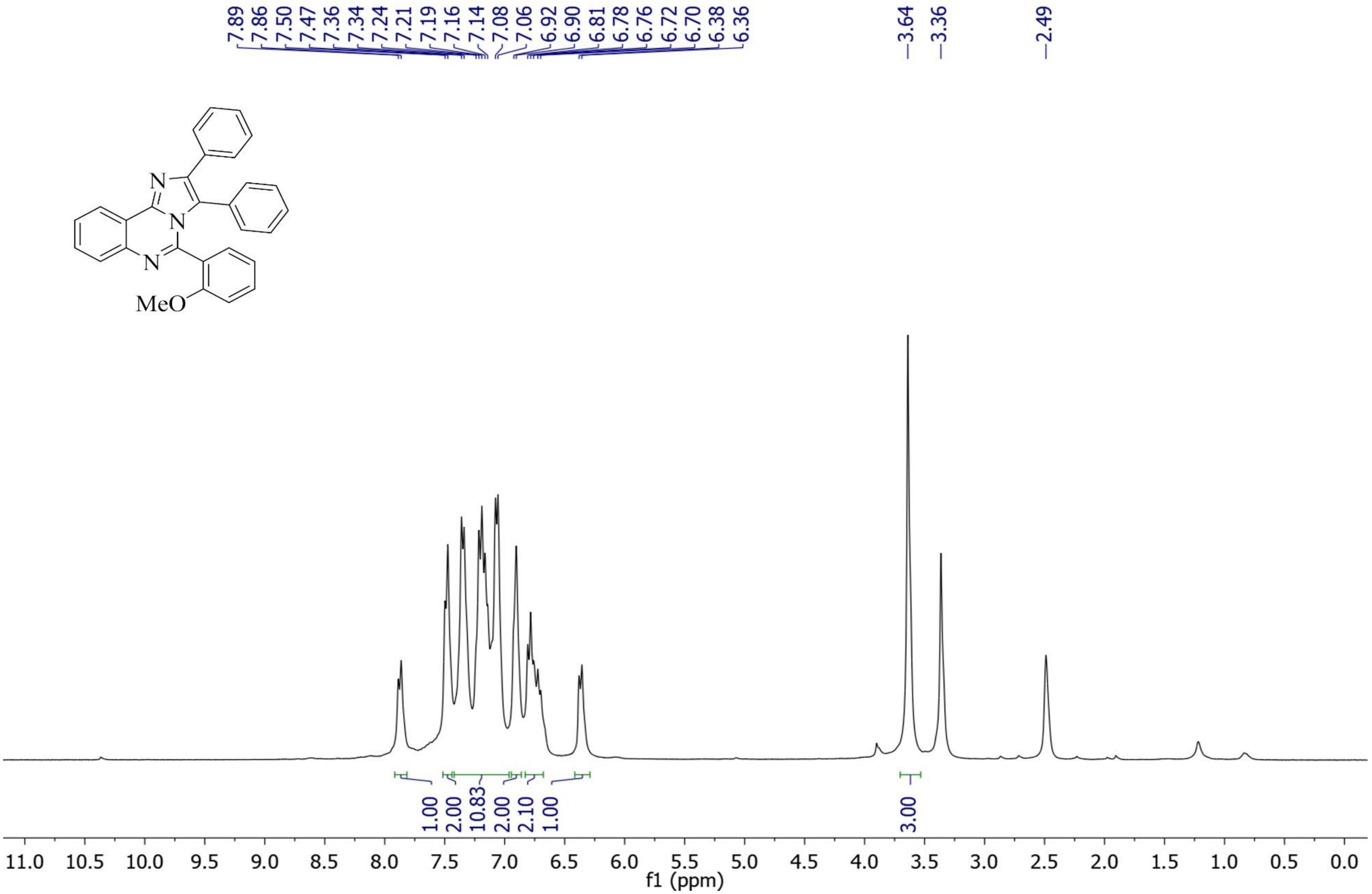

^{13}C NMR spectrum of 5-(2-methoxyphenyl)-2,3-diphenylimidazo[1,2-*c*]quinazoline **11i**

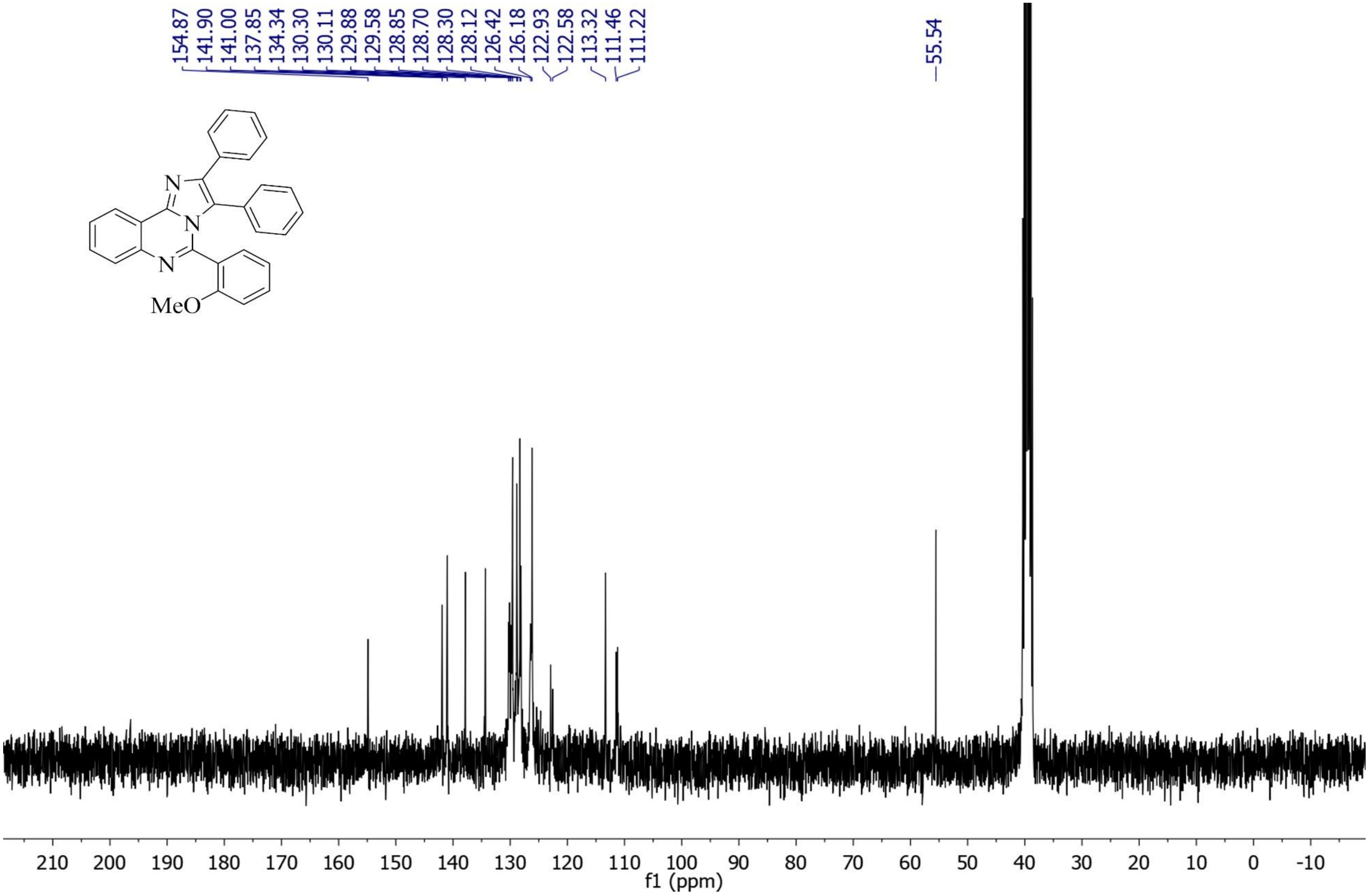

^{1}H NMR spectrum of 5-(2,3-dimethoxyphenyl)-2,3-diphenylimidazo[1,2-*c*]quinazoline **11j**

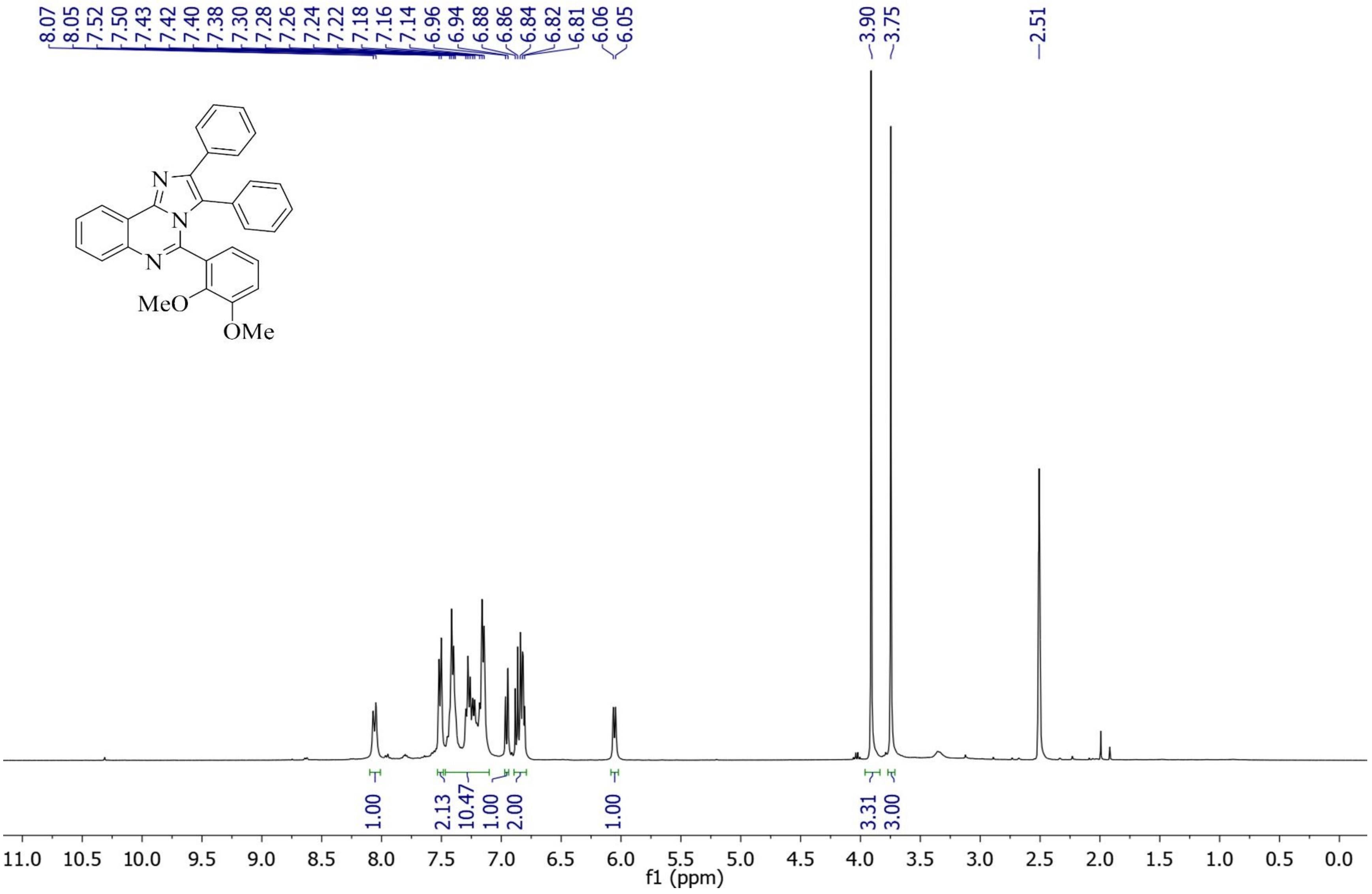

^{13}C NMR spectrum of 5-(2,3-dimethoxyphenyl)-2,3-diphenylimidazo[1,2-*c*]quinazoline **11j**

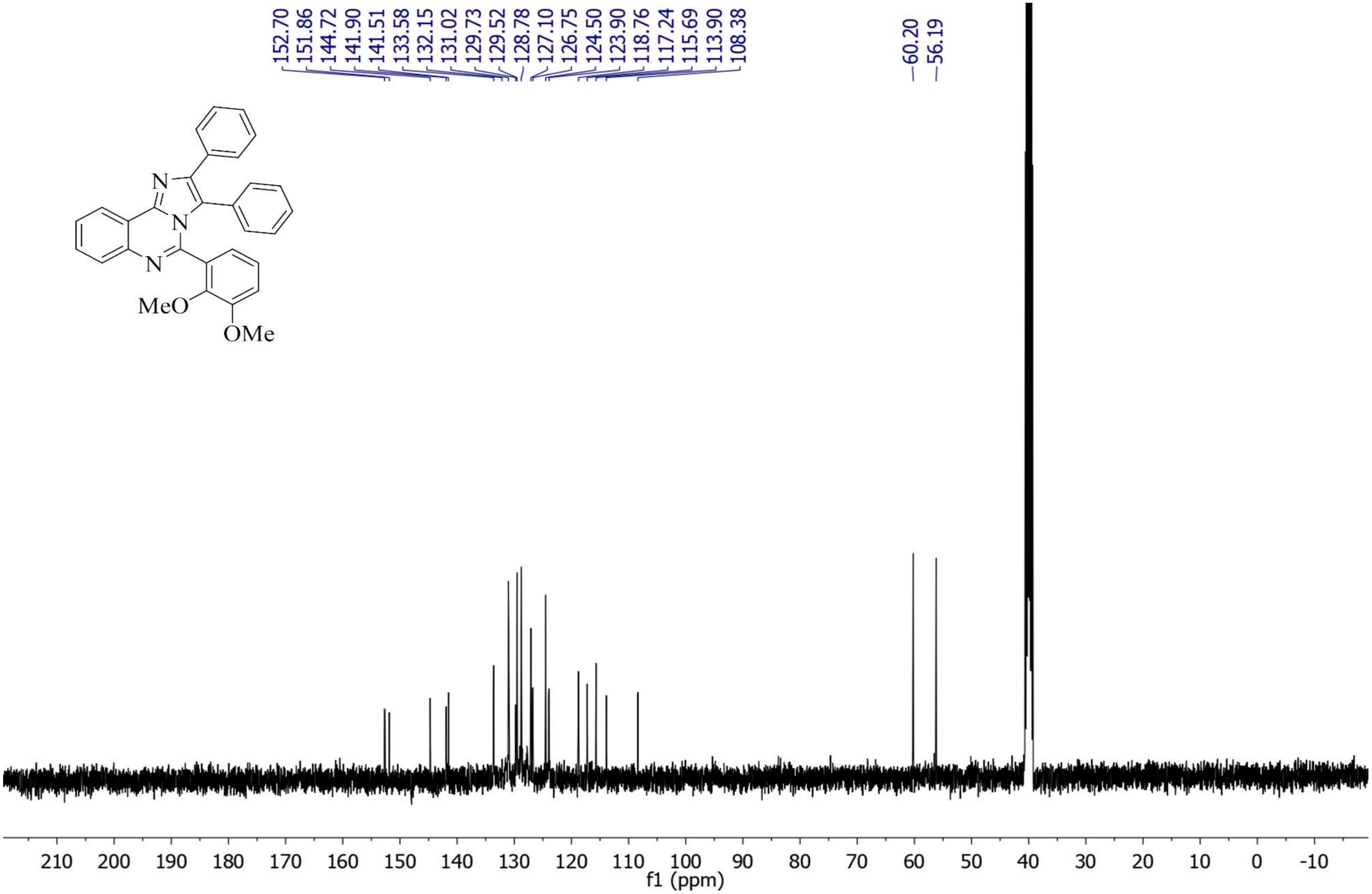

^{1}H NMR spectrum of 5-(2,4-dimethoxyphenyl)-2,3-diphenylimidazo[1,2-*c*]quinazoline **11k**

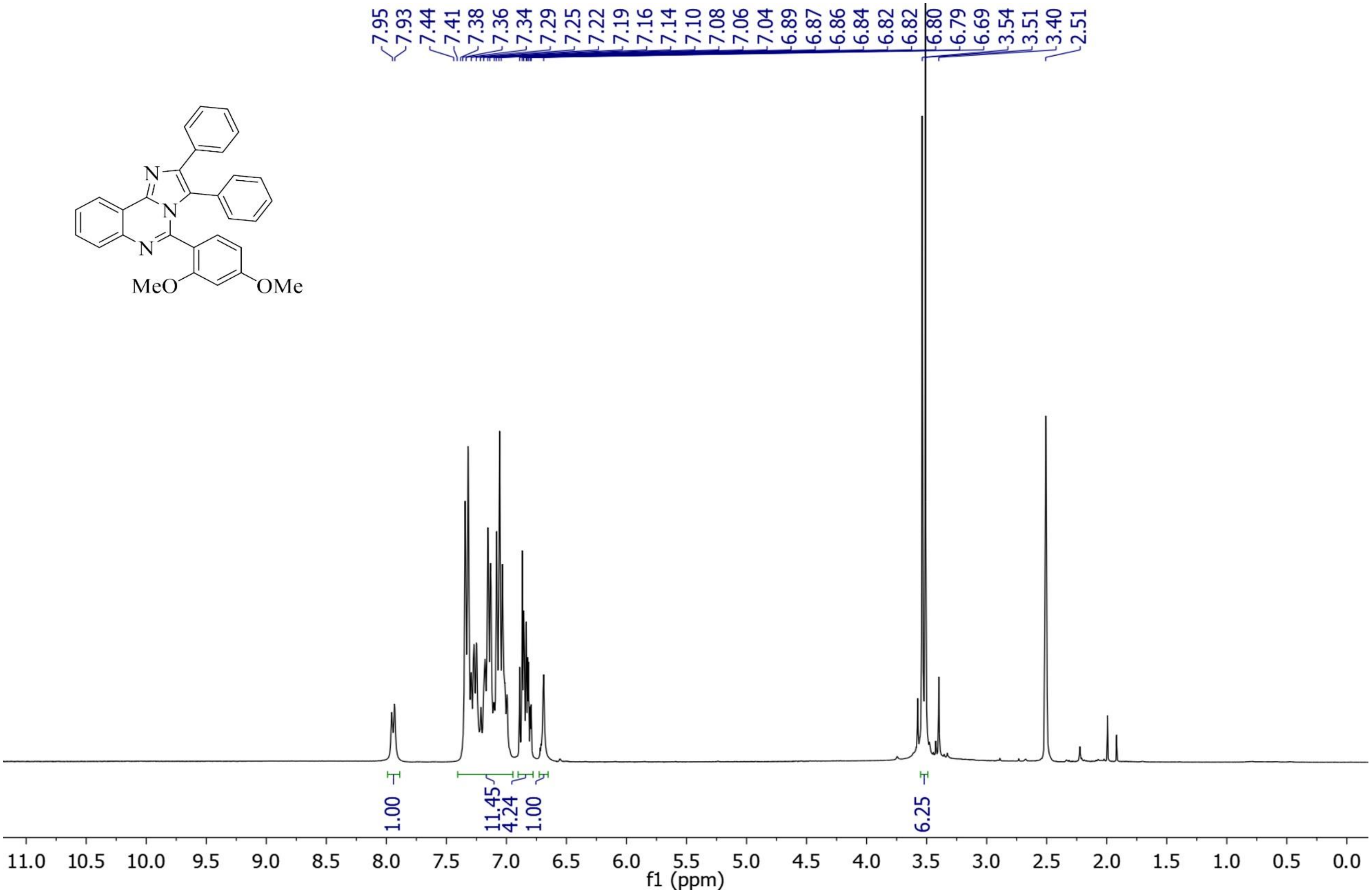

^{13}C NMR spectrum of 5-(2,4-dimethoxyphenyl)-2,3-diphenylimidazo[1,2-*c*]quinazoline **11k**

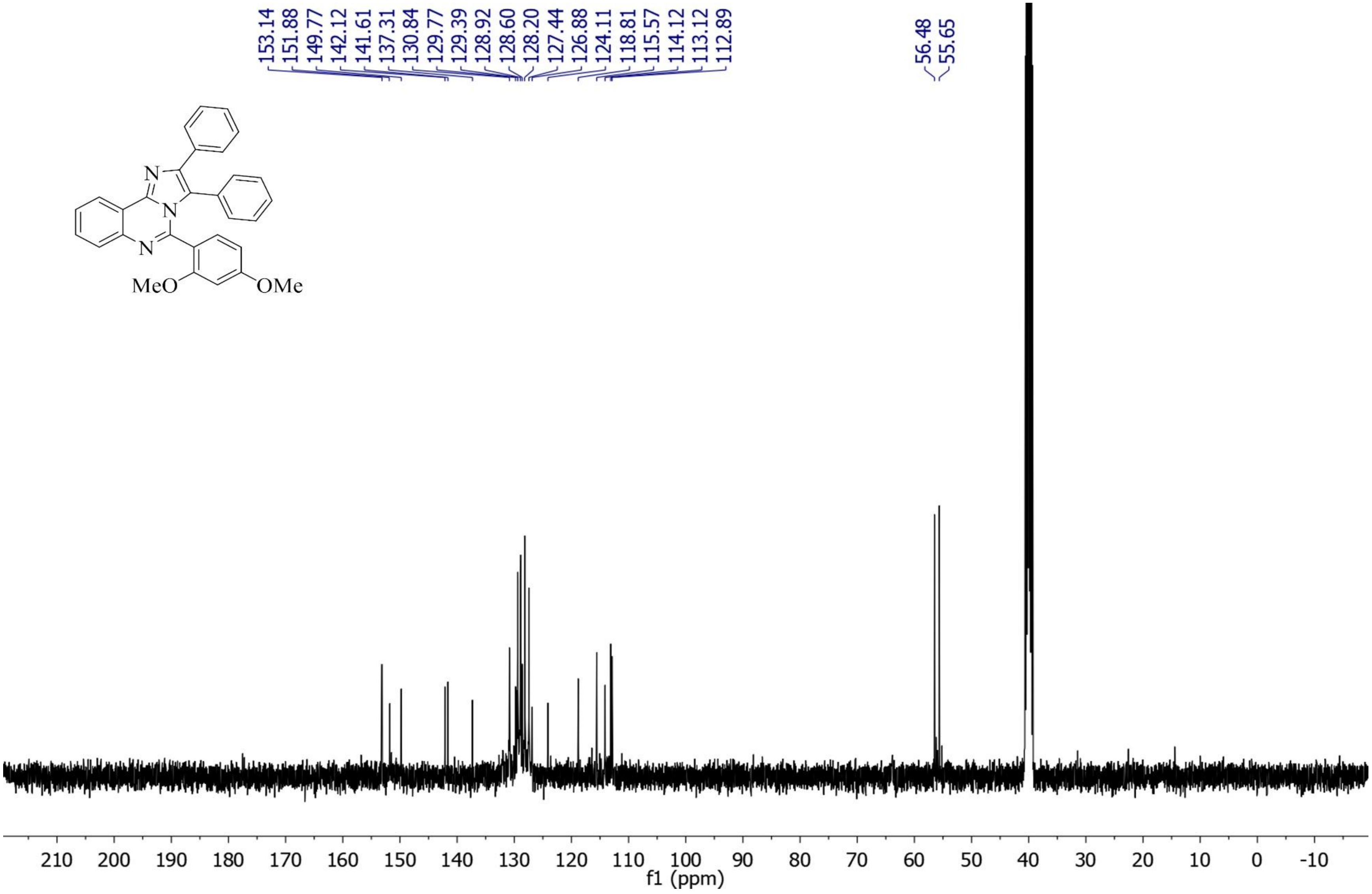

^{1}H NMR spectrum of 5-(2,5-dimethoxyphenyl)-2,3-diphenylimidazo[1,2-*c*]quinazoline **11l**

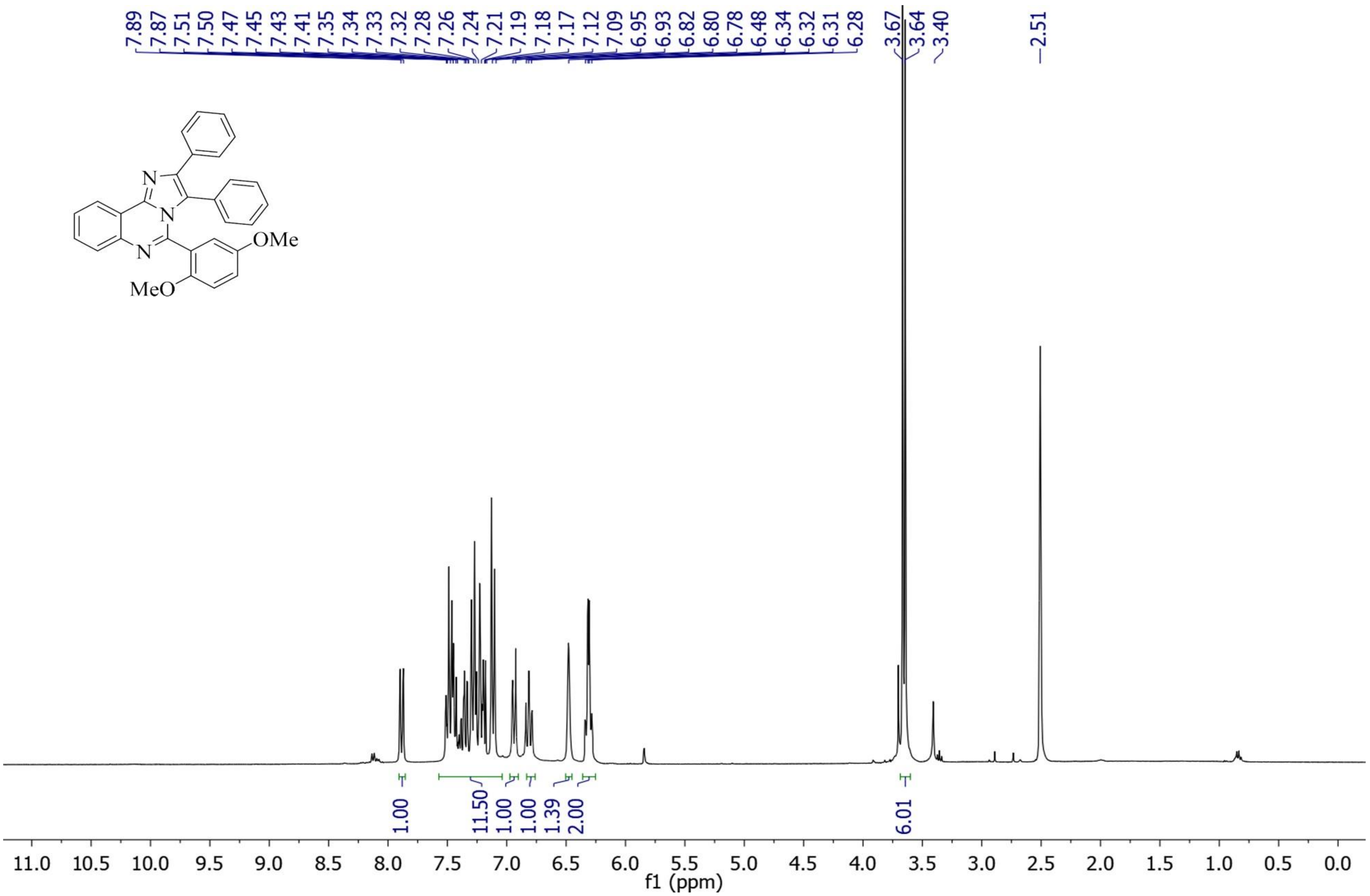

^{13}C NMR spectrum of 5-(2,5-dimethoxyphenyl)-2,3-diphenylimidazo[1,2-*c*]quinazoline **11l**

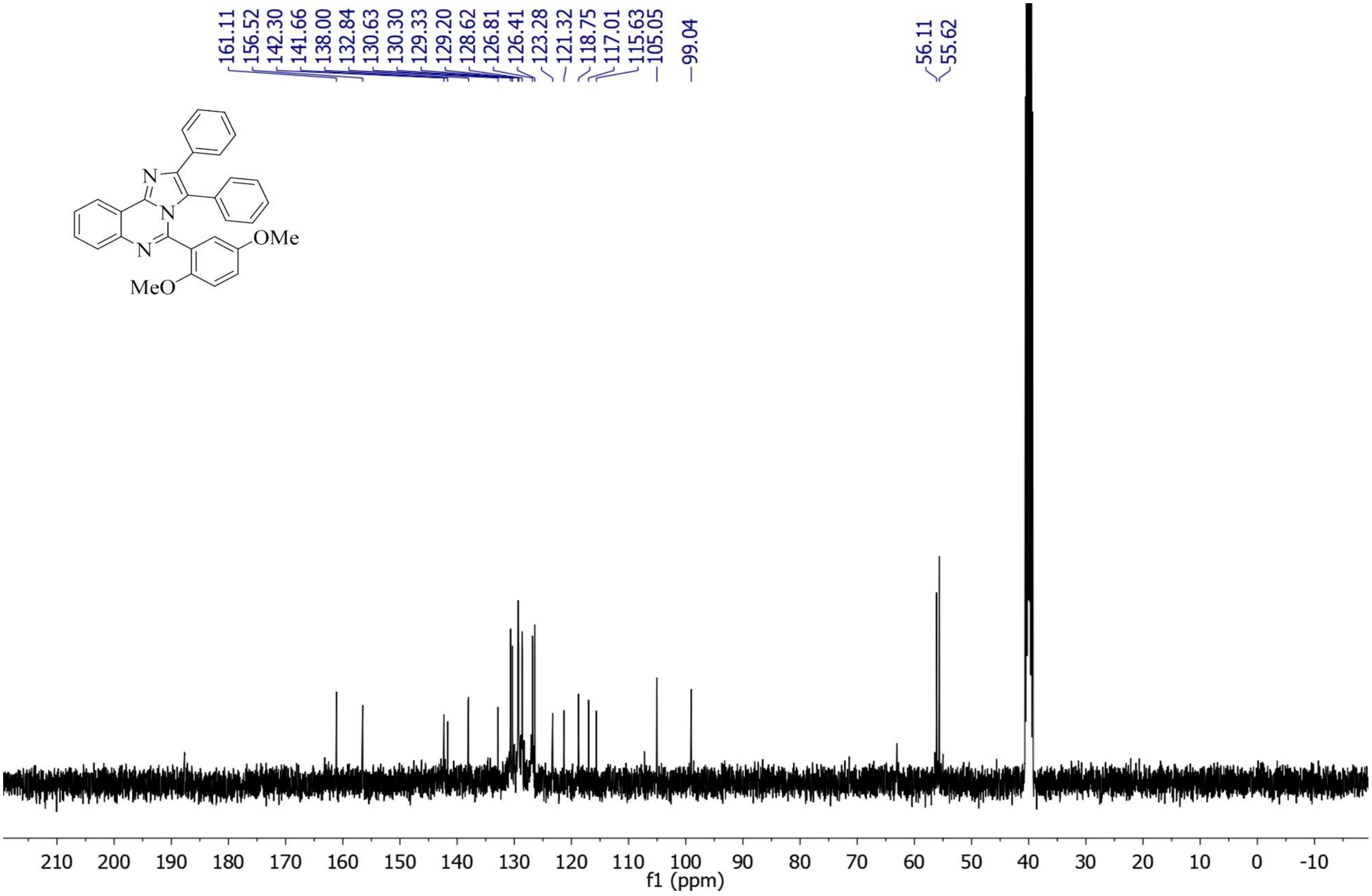

^{1}H NMR spectrum of 5-(3,4-dimethoxyphenyl)-2,3-diphenylimidazo[1,2-*c*]quinazoline **11m**

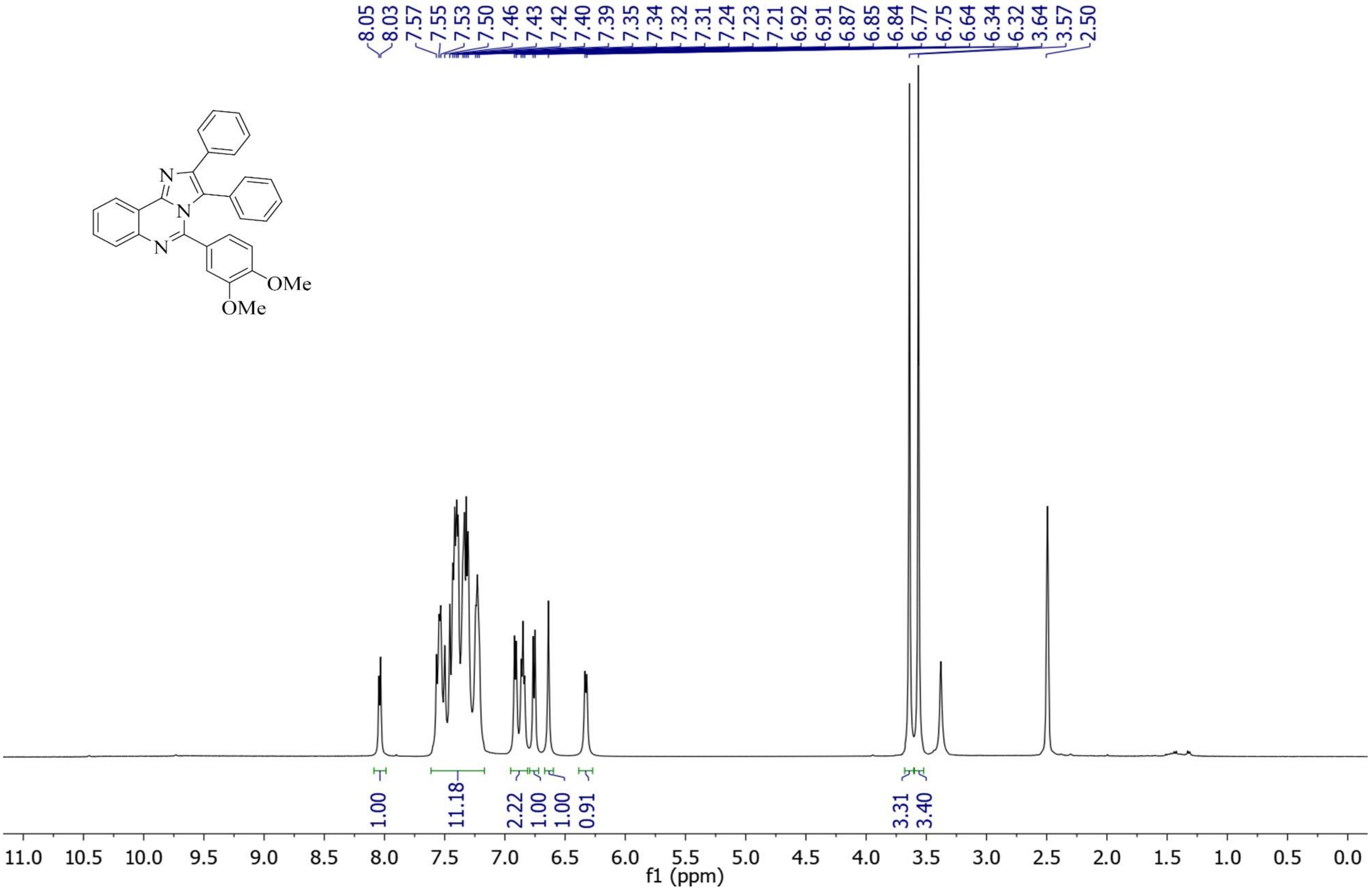

^{13}C NMR spectrum of 5-(3,4-dimethoxyphenyl)-2,3-diphenylimidazo[1,2-*c*]quinazoline **11m**

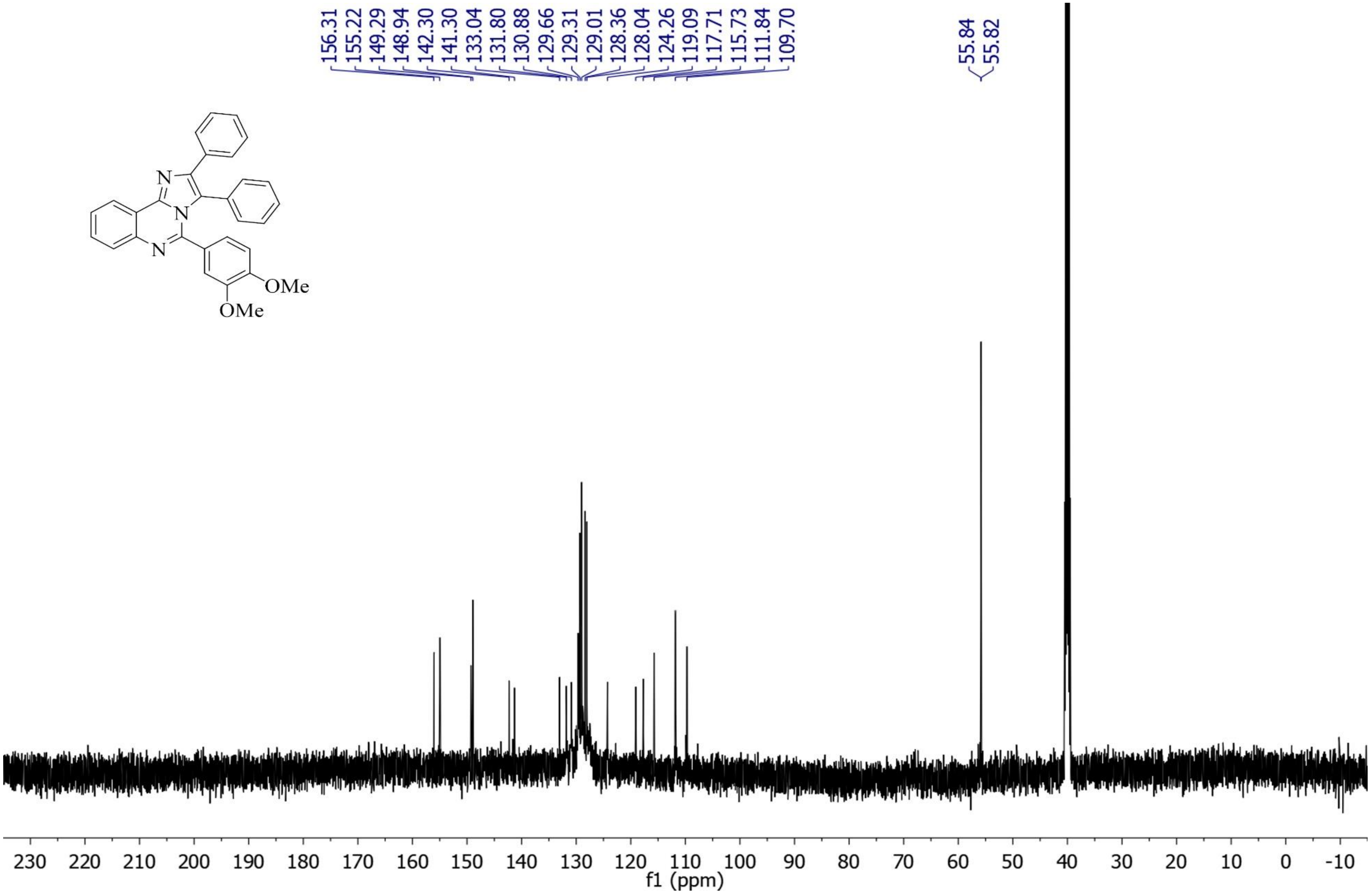

^{1}H NMR spectrum of 2,3-diphenyl-5-(3,4,5-trimethoxyphenyl)imidazo[1,2-*c*]quinazoline **11n**

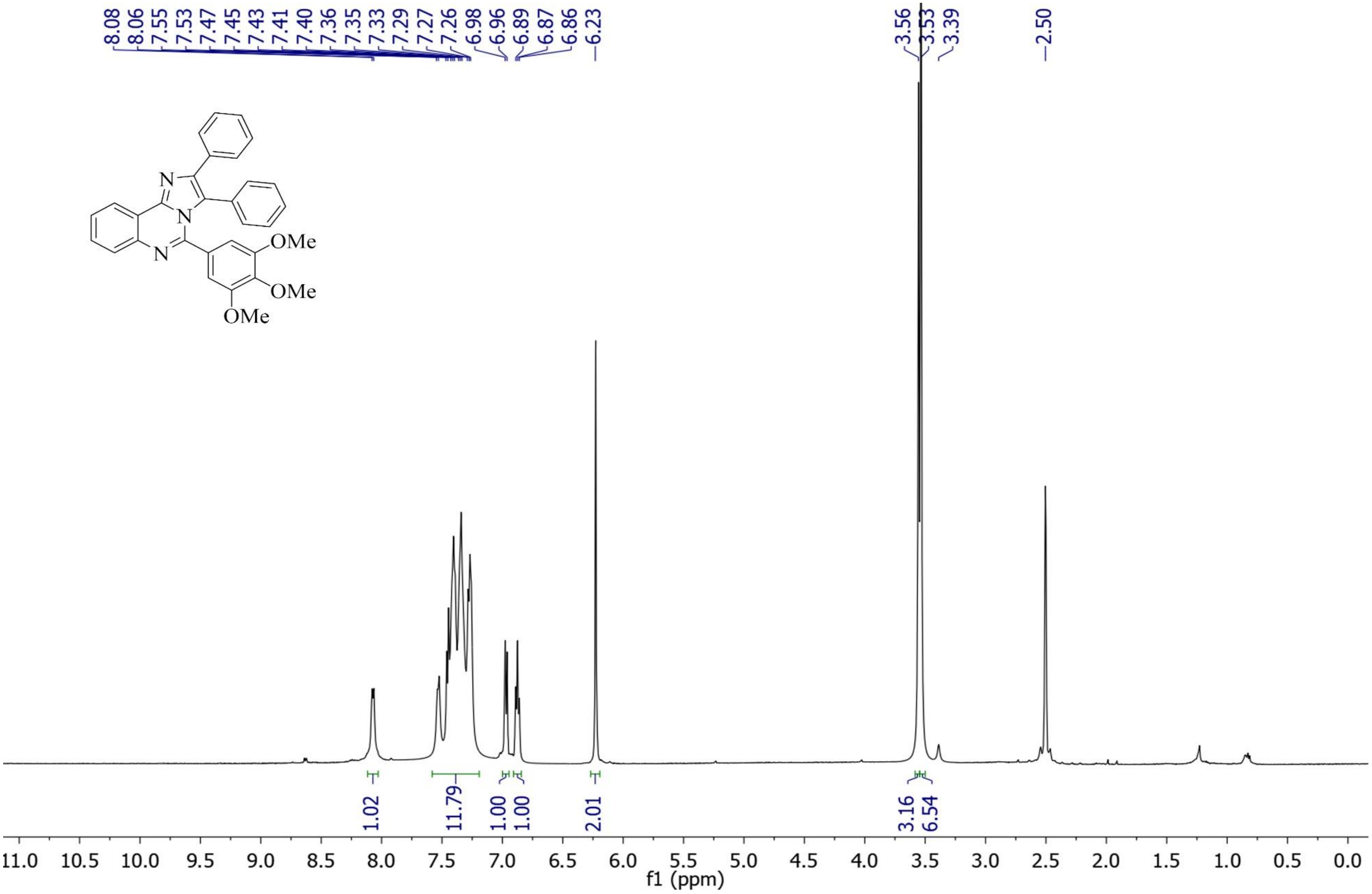

^{13}C NMR spectrum of 2,3-diphenyl-5-(3,4,5-trimethoxyphenyl)imidazo[1,2-*c*]quinazoline **11n**

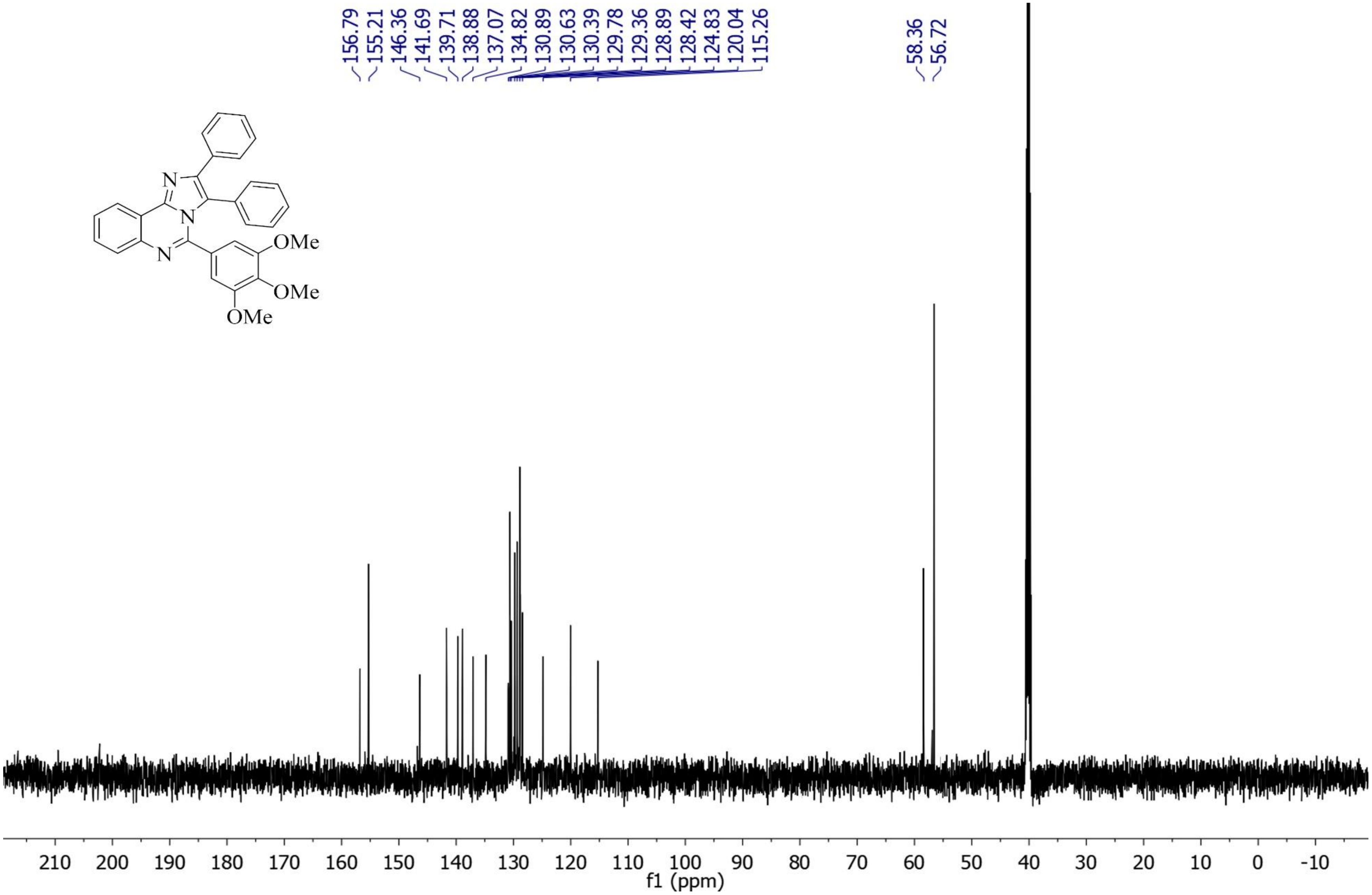

^{1}H NMR spectrum of 5-(3-bromo-4,5-dimethoxyphenyl)-2,3-diphenylimidazo[1,2-*c*]quinazoline **11o**

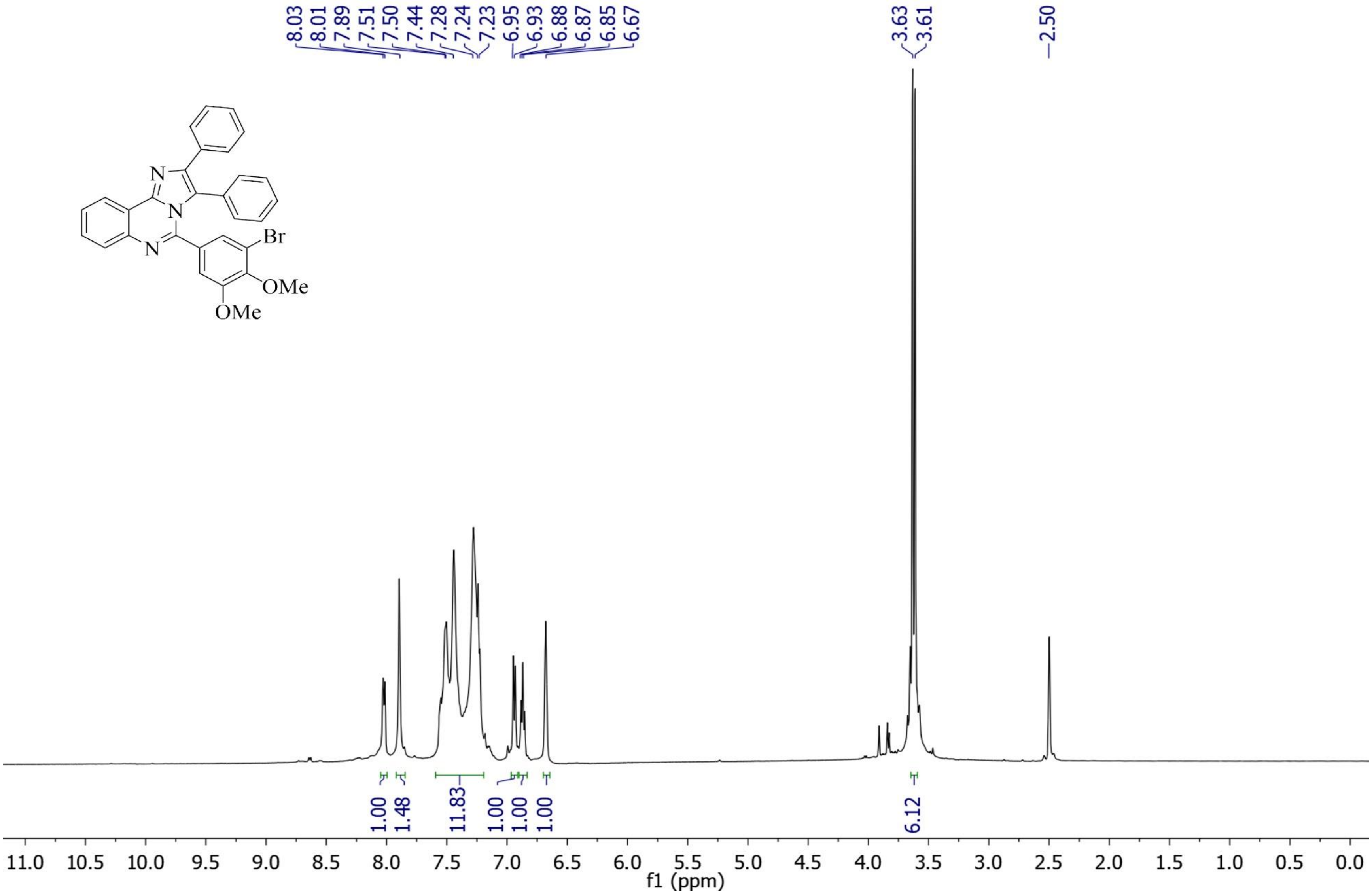

^{13}C NMR spectrum of 5-(3-bromo-4,5-dimethoxyphenyl)-2,3-diphenylimidazo[1,2-*c*]quinazoline **11o**

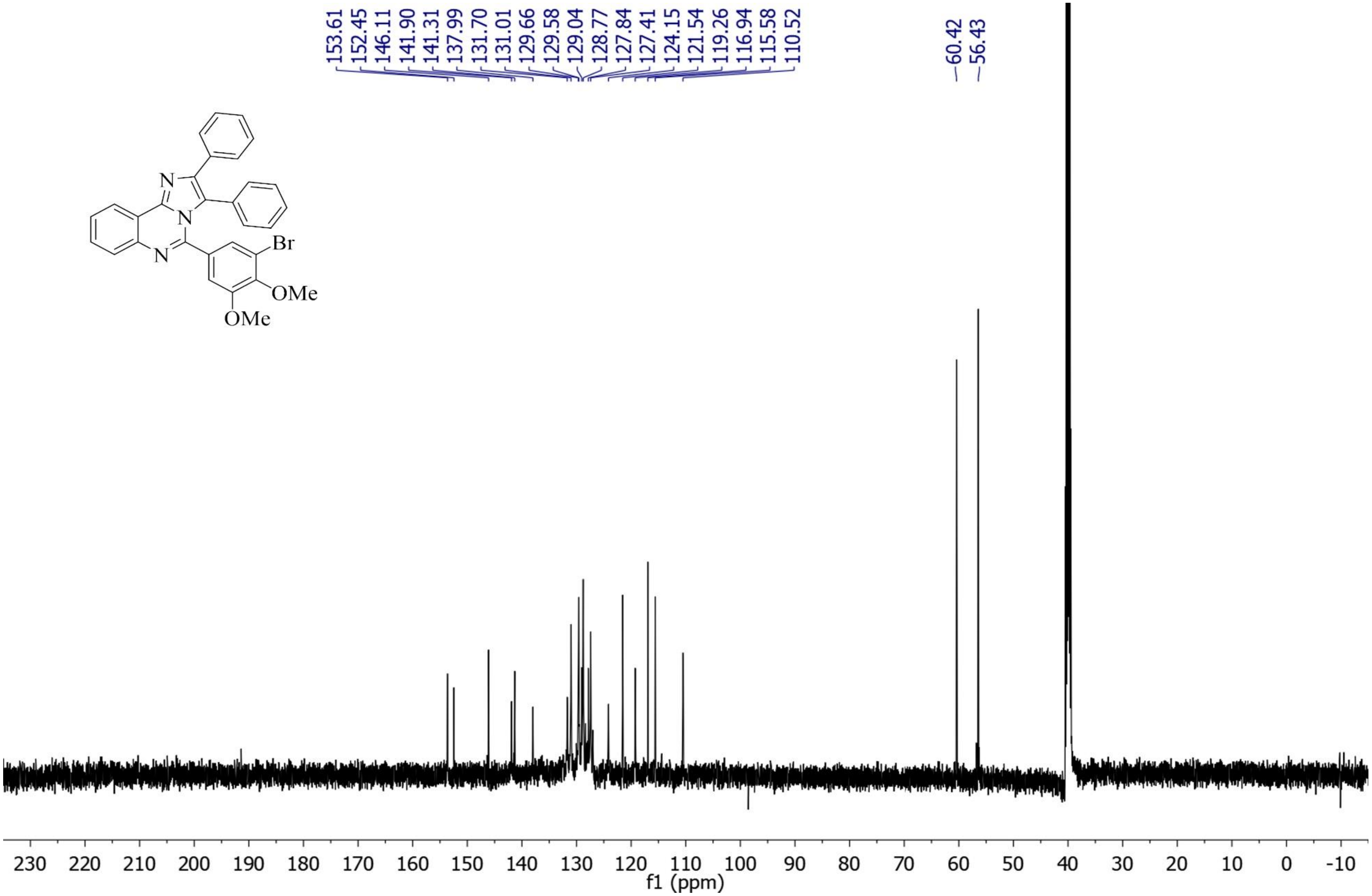

Supplement: Supplementary file 1 — Supplementary Information. [file 41598_2023_42549_MOESM1_ESM.pdf]
